# A Critical Review of High-Performance Microsupercapacitors for Next-Generation Wearable Electronics

**DOI:** 10.3390/mi17101162

**Published:** 2026-10-02

**Authors:** Ramkumar Vanaraj, Seong-Cheol Kim

**Affiliations:** 1 Department of Molecular Analytics, Saveetha School of Engineering, Saveetha Institute of Medical and Technical Sciences, Saveetha University, Thandalam, Chennai 602105, Tamil Nadu, India; 2 School of Chemical Engineering, Yeungnam University, Gyeongsan 38541, Republic of Korea

**Keywords:** microsupercapacitors, wearable electronics, solid-state electrolytes, flexible energy storage, stretchable supercapacitors

## Abstract

Microsupercapacitors (MSCs) have emerged as promising microscale energy-storage technologies for next-generation wearable, flexible, stretchable, and miniaturized electronics owing to their high-power density, rapid charge-discharge capability, excellent rate performance, and long cycle life. However, simultaneously achieving high energy density, mechanical flexibility, device reliability, scalable fabrication, safety, and sustainability remains challenging. This review critically examines recent advances in high-performance MSCs, covering fundamental charge-storage mechanisms, electrode materials, electrolytes, electrode–electrolyte interfaces, microfabrication strategies, and device architectures. Particular emphasis is placed on carbon-based materials, MXenes, transition-metal compounds, conducting polymers, and hybrid heterostructures designed to enhance charge-storage kinetics and energy density. Recent progress in planar, three-dimensional, flexible, stretchable, deformable, kirigami-inspired, liquid-metal-based, and self-healing MSCs is discussed in relation to their electrochemical and mechanical performance. The integration of MSCs with wearable sensors, smart textiles, triboelectric nanogenerators, wireless charging systems, and other emerging electronic platforms is also highlighted. Furthermore, critical challenges associated with energy–power trade-offs, mechanical durability, temperature stability, manufacturing scalability, safety, reproducibility, and environmental sustainability are evaluated. Finally, future perspectives toward multifunctional, reliable, sustainable, and autonomously integrated MSCs are proposed, providing guidance for translating laboratory-scale advances into practical energy-storage technologies for next-generation wearable electronics.

## 1. Introduction

The rapid evolution of microelectronics, microelectromechanical systems (MEMS), Internet of Things (IoT) platforms, biomedical microsystems, and wearable technologies has fundamentally changed the requirements for energy-storage devices [1,2,3]. Contemporary electronic systems are progressively becoming smaller, lighter, more flexible, and increasingly integrated with the human body, creating a demand for energy-storage technologies that can operate efficiently within highly constrained dimensions and under continuous mechanical deformation. In contrast to conventional electronic platforms, next-generation wearable systems require power sources that not only provide sufficient energy and power but also accommodate bending, folding, twisting, stretching, and repeated mechanical deformation without substantial degradation in electrochemical performance [4,5,6]. Consequently, the development of miniaturized, high-performance, mechanically compliant energy-storage devices has become an essential component of the transition toward autonomous and intelligent wearable electronics. The current scope of Micromachines explicitly encompasses micro- and nanoscale energy storage, flexible and stretchable electronics, wearable systems, microfabrication, and microsystem-level integration, making microsupercapacitors (MSCs) particularly relevant to the journal’s interdisciplinary focus. Wearable electronics encompass a rapidly expanding range of technologies, including physiological and biochemical sensors, electronic skin (e-skin), smart textiles, flexible displays, human-machine interfaces, wireless communication platforms, healthcare-monitoring devices, and low-power IoT nodes [7,8,9]. These systems increasingly require continuous operation in environments characterized by dynamic mechanical motion and intimate contact with curved or deformable surfaces. Accordingly, energy-storage components must satisfy requirements that extend beyond conventional electrochemical metrics. High energy density and power density must be accompanied by mechanical flexibility, structural integrity, long cycle life, low thickness, lightweight construction, operational safety, and compatibility with scalable fabrication processes. Conventional rigid batteries and supercapacitors can provide substantial energy or power; however, their form factors, rigidity, packaging requirements, and limited compatibility with microscale fabrication can impose significant restrictions on their integration into highly miniaturized wearable platforms. Flexible supercapacitors have therefore attracted considerable interest because of their rapid charge-discharge capability, high power density, long cycling stability, and potential mechanical adaptability [10,11,12]. Within this context, microsupercapacitors have emerged as a distinctive class of electrochemical energy-storage devices specifically designed for miniaturized and integrated electronic systems. MSCs retain the fundamental charge-storage principles of conventional supercapacitors while employing microscale electrode dimensions, patterned architectures, reduced interelectrode distances, and fabrication strategies compatible with microelectronics. Their small footprint and high-rate operation make them particularly attractive for powering microsensors, wireless systems, MEMS/NEMS devices, implantable electronics, and wearable platforms. At the microscale, shortened ion-transport pathways and carefully engineered electrode geometries can facilitate rapid electrochemical kinetics, while planar and interdigitated architectures can enable direct integration with electronic substrates. Recent research has consequently moved beyond simply reducing the dimensions of conventional supercapacitors toward the deliberate engineering of materials, interfaces, device architectures, and fabrication processes specifically for microscale energy storage [13,14,15].

The significance of MSCs for wearable electronics arises not merely from their small size but from the possibility of integrating energy storage directly into the architecture of a wearable system. Conventional energy-storage devices are frequently manufactured as discrete components and subsequently connected to electronic circuits. Such an approach can increase the overall footprint, introduce electrical and mechanical interconnections, and compromise the flexibility of the complete system. In contrast, MSCs can potentially be fabricated directly on or within suitable substrates, enabling compact and distributed energy-storage architectures. Planar, interdigitated, fiber-shaped, vertically aligned, three-dimensional, and multilayer configurations have consequently been explored to maximize electrochemical performance while maintaining dimensional compatibility with miniaturized electronics. The integration of MSCs into wearable systems therefore represents a transition from component-level energy storage toward architecture-driven and system-level energy management. The performance of an MSC is strongly governed by the synergistic relationship among electrode materials, electrolyte chemistry, electrode architecture, current collectors, substrate properties, and fabrication methodology [16,17,18]. Carbonaceous materials such as activated carbon, graphene, carbon nanotubes, porous carbons, and laser-induced graphene have attracted substantial attention because of their high electrical conductivity, tunable porosity, relatively low density, and suitability for electric double-layer charge storage. At the same time, two-dimensional materials, particularly MXenes, have introduced opportunities for combining high conductivity with surface redox activity and ion-accessible layered structures. Conducting polymers and transition-metal compounds can provide enhanced pseudocapacitance, whereas metal-organic frameworks (MOFs), covalent organic frameworks (COFs), and their derivatives offer opportunities for molecular-level control of porosity, composition, and electroactive sites. Nevertheless, the high theoretical performance of an individual material does not necessarily translate into superior device-level performance. Restacking of two-dimensional materials, structural collapse, poor mechanical integrity, low active-material utilization, sluggish ion transport within thick electrodes, and degradation during repeated deformation remain important barriers to practical implementation. Consequently, current research increasingly emphasizes hierarchical and hybrid electrode structures rather than isolated active materials. Combining highly conductive carbon frameworks with pseudocapacitive materials, constructing porous three-dimensional architectures, engineering heterointerfaces, introducing heteroatoms, and controlling pore-size distributions are among the strategies used to simultaneously improve electronic transport, ionic accessibility, and electrochemical activity. However, these strategies also introduce new trade-offs. Increasing the loading of active materials may improve areal capacitance but can increase ion-diffusion resistance; highly porous structures may promote electrolyte accessibility but reduce volumetric energy density; and mechanically robust polymeric components may improve flexibility while decreasing electrical conductivity. Therefore, the development of genuinely high-performance MSCs requires a balanced optimization of electrochemical, structural, mechanical, and manufacturing parameters rather than maximizing a single performance metric [19,20,21].

Electrolytes represent another critical component of MSC technology. Aqueous electrolytes offer high ionic conductivity and relatively low cost, whereas organic electrolytes and ionic liquids can provide wider electrochemical stability windows and potentially higher operating voltages [22,23,24]. Gel polymer, hydrogel, ionogel, and solid-state electrolytes are particularly attractive for flexible and wearable MSCs because they can simultaneously function as ion-conducting media and mechanically compliant components. Nevertheless, electrolyte selection directly influences operating voltage, ionic transport, self-discharge, safety, environmental stability, mechanical durability, and long-term device reliability. For wearable applications, additional considerations such as leakage resistance, dehydration, skin compatibility, biocompatibility, encapsulation, and operation under varying temperature and humidity become increasingly important. Thus, electrolyte engineering must be considered together with electrode and structural design rather than as an independent optimization variable. Microfabrication is equally important because the performance and practicality of an MSC depend strongly on the ability to precisely control electrode dimensions, spacing, thickness, and architecture. Photolithography, laser writing, laser-induced graphene formation, electrodeposition, printing, chemical vapor deposition, atomic layer deposition, screen printing, inkjet printing, transfer techniques, and additive manufacturing have all provided different routes toward MSC fabrication. These approaches differ substantially in terms of spatial resolution, processing temperature, material compatibility, throughput, cost, scalability, and suitability for flexible substrates [25,26,27]. While advanced lithographic techniques can provide highly precise microelectrode patterns, they may require multiple processing steps and specialized infrastructure. Printing and laser-based approaches offer attractive alternatives for rapid patterning and scalable manufacturing, particularly for flexible substrates. Therefore, an important unresolved question is not simply which fabrication method provides the highest laboratory-scale performance, but which method can reproducibly produce high-performance MSCs at technologically relevant scale while maintaining device uniformity and mechanical reliability. Device architecture is another decisive factor in determining MSC performance. In-plane interdigitated structures are particularly attractive because they provide short ion-diffusion distances and enable integration onto planar substrates. Sandwich-type architectures can achieve high electrode loading but may have greater thickness and longer ionic transport pathways. Three-dimensional and vertically aligned architectures offer opportunities for increasing active surface area and volumetric performance, whereas fiber-shaped and textile-integrated configurations are promising for conformal wearable systems. Similarly, asymmetric architectures can expand the operating voltage window by combining electrodes with different charge-storage mechanisms. These architectural innovations demonstrate that MSC performance cannot be interpreted solely from the intrinsic properties of the electrode material. Instead, electrochemical behavior emerges from the interaction between material chemistry and device geometry. An additional challenge is the appropriate evaluation and comparison of MSC performance. Conventional gravimetric capacitance and energy density are often insufficient to describe the practical value of microscale devices because the mass of active material may be extremely small and may represent only a fraction of the complete device. For wearable and integrated systems, areal and volumetric capacitance, energy density, power density, footprint, thickness, and mechanical stability can be more meaningful parameters. Nevertheless, inconsistencies in normalization procedures, electrode-area definitions, inclusion or exclusion of current collectors and substrates, electrolyte contributions, and device-level versus material-level measurements can complicate comparisons among reported MSCs. A critical evaluation of these parameters is therefore essential for identifying genuine technological progress and distinguishing intrinsic material performance from improvements arising primarily from device geometry or measurement methodology [28,29,30].

**Figure 1:**
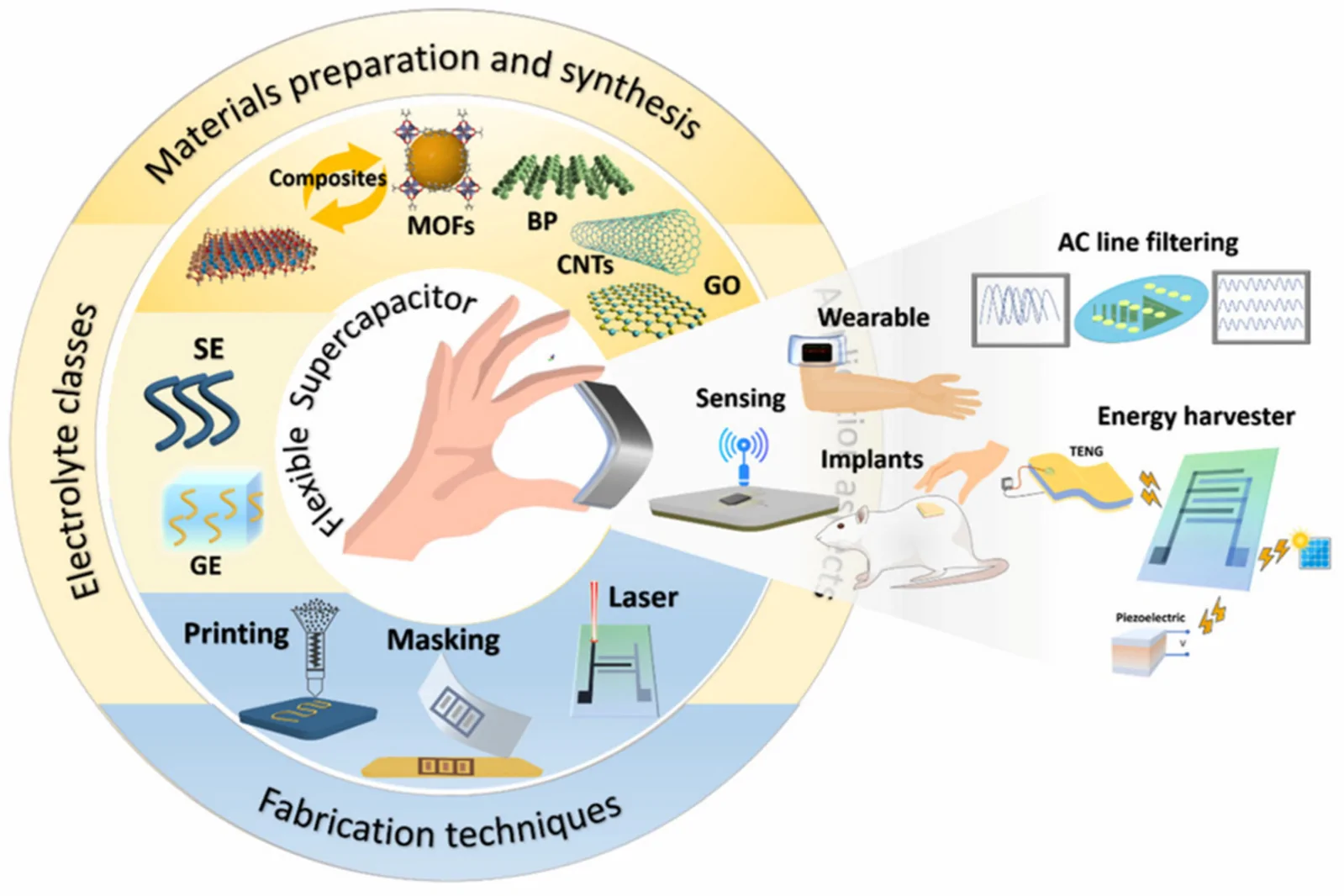
Evolution of microsupercapacitors from conventional electrochemical capacitors toward miniaturized, high-power energy-storage devices for flexible and wearable electronics. Reproduced with permission from Ref. [31]. Copyright (2024) Elsevier.

Mechanical durability represents another fundamental criterion for wearable MSCs. Unlike stationary energy-storage devices, wearable systems experience repeated bending, stretching, twisting, compression, and folding during normal operation. Mechanical deformation can alter electrical pathways, induce cracking or delamination, disrupt electrode–electrolyte interfaces, and accelerate electrolyte loss. Consequently, a high initial capacitance is insufficient to establish the suitability of an MSC for wearable applications. Long-term electrochemical retention under repeated mechanical deformation, stable electrode adhesion, resistance to environmental exposure, and reliable encapsulation must also be considered. Emerging concepts such as self-healing materials, stretchable serpentine architectures, kirigami and origami structures, textile-integrated electrodes, and intrinsically deformable polymeric components are therefore becoming increasingly important for achieving mechanically robust energy-storage systems. The ultimate objective of MSC research is increasingly shifting from isolated device fabrication toward integration with functional wearable systems. MSCs can serve as local power sources for sensors, electronic skins, wireless communication modules, biomedical monitoring platforms, and low-power IoT devices. They can also be integrated with energy harvesters, including photovoltaic, triboelectric, piezoelectric, and thermoelectric systems, to create self-powered or intermittently powered electronic platforms. Such integration introduces a new set of requirements related to charge-management strategies, power matching, electrical interconnection, packaging, system footprint, and operational reliability. Recent research in the field has consequently begun to emphasize MSC-powered sensing and integrated microsystems rather than evaluating energy-storage devices exclusively through electrochemical measurements [31,32,33]. Despite substantial progress, important gaps remain between laboratory-scale MSC demonstrations and practical wearable energy-storage technologies. Previous reviews have provided valuable foundations by discussing flexible MSC materials, fabrication methods, structural designs, and wearable applications. For example, a 2021 Micromachines review comprehensively addressed flexible MSCs, including printing, photolithography, screen printing, laser processing, materials engineering, electrolyte optimization, and integrated wearable systems. Other reviews have similarly examined flexible supercapacitor fabrication and structural configurations. However, the rapid development of advanced electrode chemistries, microfabrication technologies, three-dimensional architectures, solid-state electrolytes, mechanically adaptive structures, and system-level integration requires a renewed critical assessment. More importantly, recent progress highlights the need to distinguish laboratory demonstrations based on exceptional normalized metrics from device concepts that possess realistic pathways toward scalable fabrication, mechanical reliability, and practical wearable operation (Figure 1) [31]. This distinction is particularly important because the field is increasingly moving from material discovery toward device engineering, manufacturing, and system integration [34,35,36].

Accordingly, this review provides a critical and technology-oriented assessment of high-performance microsupercapacitors for next-generation wearable electronics. Rather than presenting a conventional catalog of electrode materials, the review establishes a materials-to-system framework encompassing electrode materials, charge-storage mechanisms, electrolytes, electrode–electrolyte interfaces, microfabrication technologies, device architectures, mechanical design, performance optimization, and wearable-system integration. Particular emphasis is placed on understanding the structure-property-performance relationships governing MSC behavior and on identifying the trade-offs among energy density, power density, mechanical flexibility, durability, device footprint, manufacturing scalability, and sustainability. Advanced carbon materials, MXenes, conducting polymers, transition-metal compounds, MOF/COF-derived materials, heterostructures, and hierarchical composites are critically compared alongside emerging electrolyte systems and fabrication technologies. Furthermore, this review evaluates how microscale architecture and manufacturing methodology influence practical device performance, with specific attention to planar and three-dimensional structures, flexible and stretchable configurations, printed and laser-fabricated devices, and integrated MSC arrays. The role of MSCs as functional energy-storage units in wearable sensors, electronic skin, smart textiles, healthcare-monitoring systems, IoT platforms, and self-powered microsystems is also examined. Beyond reporting record electrochemical values, emphasis is placed on standardized performance evaluation, mechanical reliability, long-term stability, packaging, safety, scalability, and system-level integration. Finally, the unresolved scientific and engineering challenges are critically analyzed to establish a forward-looking roadmap for MSC development. The central perspective of this review is that the future of MSCs will not be determined by the discovery of a single electrode material with record capacitance. Instead, meaningful technological advancement will depend on the coordinated optimization of materials chemistry, ion/electron transport, microfabrication, device architecture, mechanical engineering, electrolyte design, packaging, and system integration. The transition from high-performing laboratory prototypes to practical wearable power sources therefore requires a shift from material-centric optimization toward integrated device and system engineering. Through this perspective, the review aims to provide researchers and engineers with a comprehensive framework for designing high-performance MSCs that are not only electrochemically competitive but also mechanically resilient, manufacturable, integrable, and ultimately suitable for the next generation of intelligent wearable electronics. In this review, MSC technologies are critically evaluated using a materials-to-device-to-system framework. The assessment considers electrochemical performance, ion and electron transport, electrode–electrolyte interactions, device architecture, mechanical reliability, fabrication scalability, reproducibility, safety, and system integration. Particular emphasis is placed on identifying the trade-offs between energy and power capability, electrode loading and ion transport, mechanical flexibility and structural integrity, and device performance and manufacturing complexity. Thus, the reported performance values are considered together with their testing conditions, normalization basis, device configuration, and practical limitations rather than as isolated record values.

## 2. Fundamentals of Microsupercapacitors

**Figure 2:**
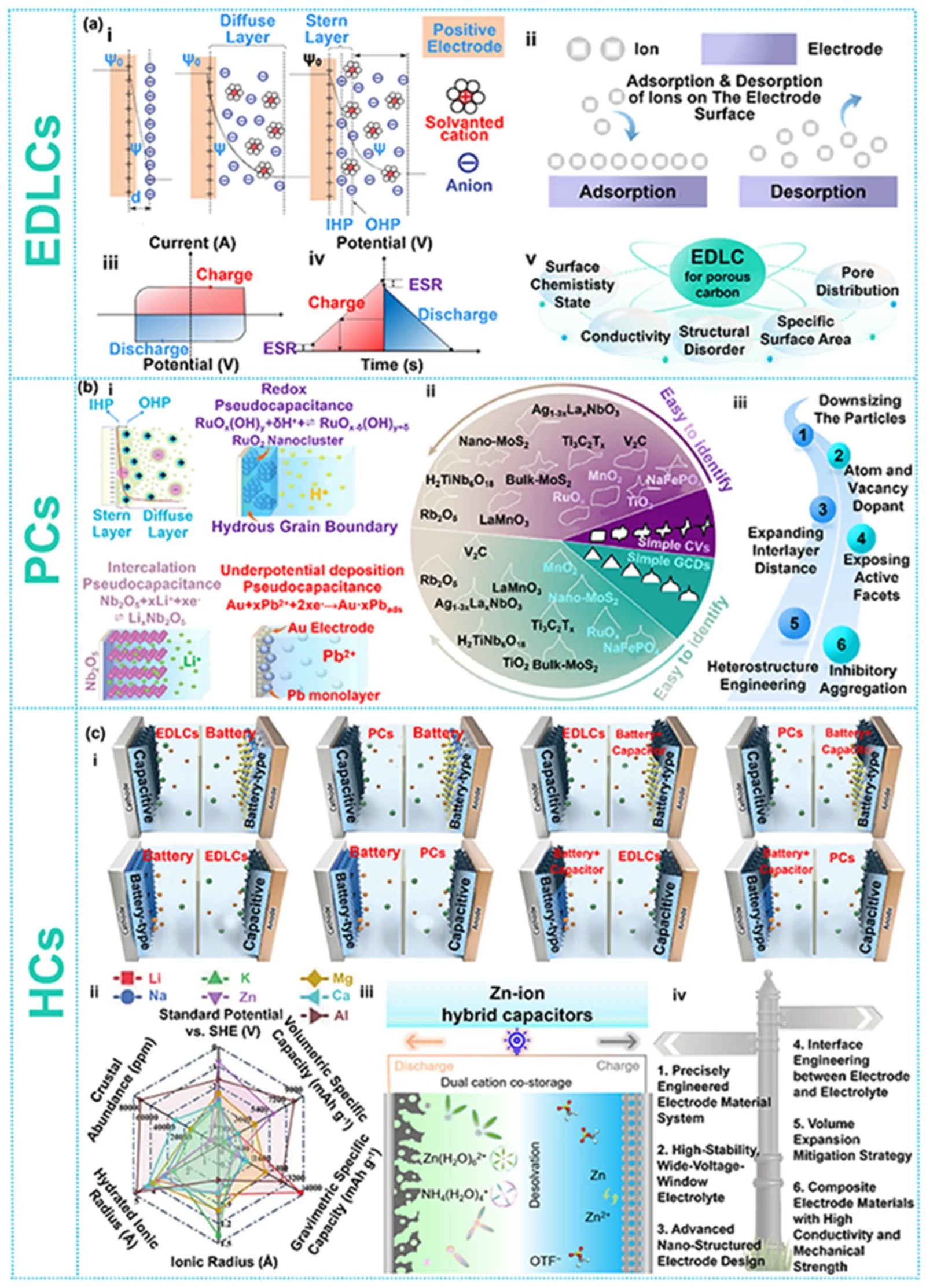
Fundamental charge-storage mechanisms in microsupercapacitors, including electric-double-layer capacitance (**a**) and pseudocapacitive charge storage (**b**), together HCs (**c**) with the corresponding ion and electron transport pathways. Reproduced with permission from Ref. [41]. Copyright (2026) Elsevier.

**Figure 3:**
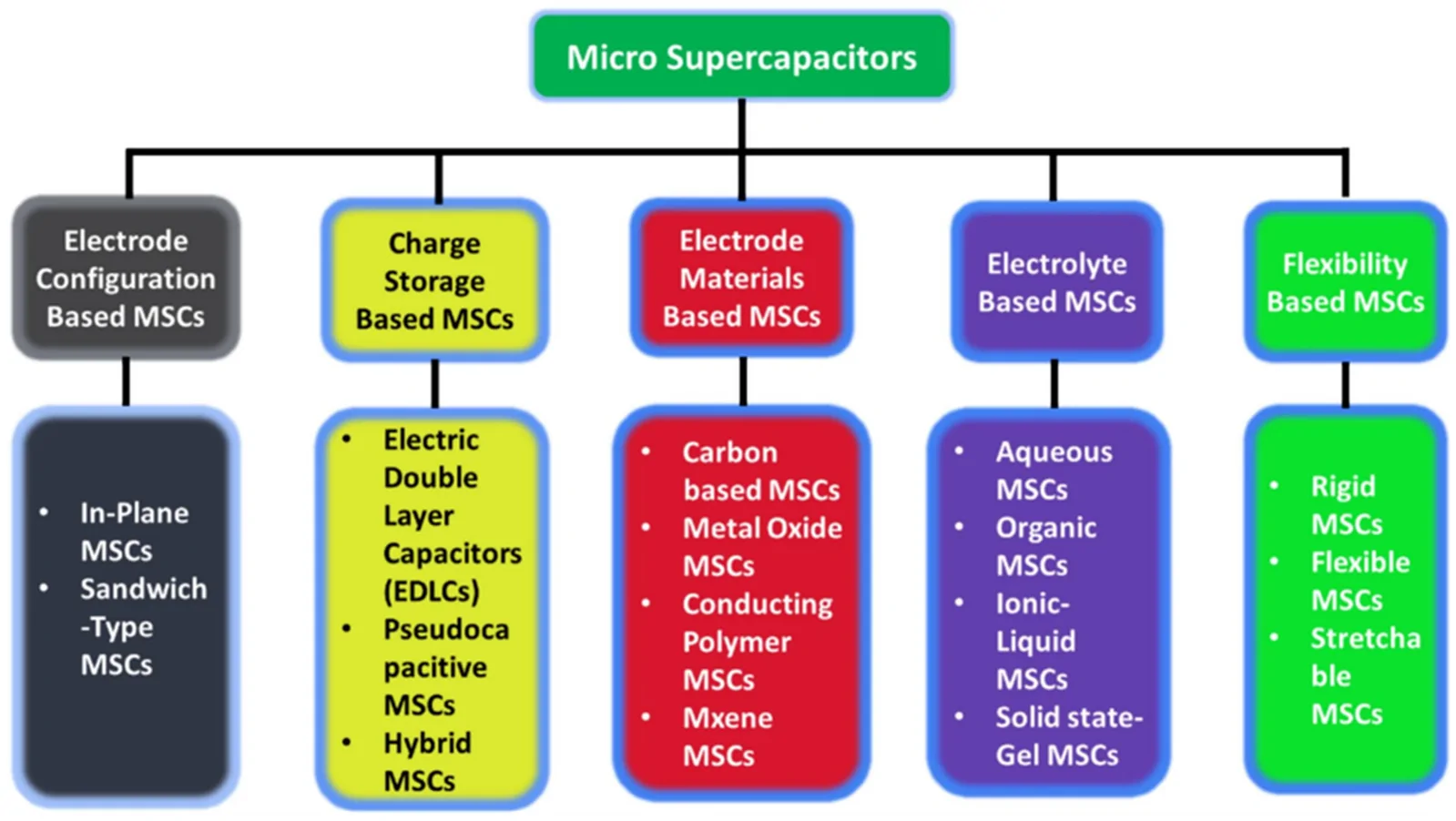
Types and classification of MSC based on structure and functional property. Reproduced with permission from Ref. [41]. Copyright (2026) Elsevier.

Microsupercapacitors (MSCs) are miniaturized electrochemical energy-storage devices developed to provide high-rate energy storage within the limited dimensions of microelectronic, wearable, flexible, and implantable systems [37,38,39]. Although MSCs originate from the same fundamental electrochemical principles as conventional supercapacitors, their operation and performance are strongly influenced by microscale geometry, electrode configuration, interfacial phenomena, ion transport, and fabrication processes (Figure 2a–c) [40,41]. Conventional supercapacitors generally employ relatively large electrodes separated by a porous separator and electrolyte, whereas MSCs commonly utilize micro-patterned electrodes with characteristic dimensions ranging from micrometers to millimeters. This dimensional reduction enables shorter ionic diffusion pathways, reduced device footprint, and direct integration with microsystems, but simultaneously introduces challenges associated with limited active-material volume, increased influence of current collectors and substrates, interfacial resistance, and fabrication-induced defects. Therefore, MSCs should not simply be regarded as geometrically downsized conventional supercapacitors; rather, they represent a distinct class of energy-storage devices in which electrochemical behavior is closely coupled with microstructural and architectural design. The fundamental operating principle of an MSC is based on reversible charge storage at the electrode–electrolyte interface [42,43,44]. Depending on the nature of the electrode material and the dominant charge-storage mechanism, MSCs can be broadly classified as electric double-layer capacitors (EDLCs), pseudocapacitors, and hybrid devices. In EDLC-type MSCs, charge is stored electrostatically through the formation of an electric double layer at the interface between the electrode and electrolyte (Figure 3) [41]. When an external potential is applied, electrons accumulate within the electrode, while oppositely charged electrolyte ions migrate toward the electrode surface to maintain electroneutrality. The resulting spatial separation of electronic and ionic charges produces capacitance without requiring significant chemical transformation of the electrode. At the interfacial level, the capacitance is influenced by the effective dielectric properties of the interfacial region, the accessible surface area, and the separation between electronic and ionic charge. Consequently, electrode materials possessing high surface area, interconnected porosity, good electrical conductivity, and favorable electrolyte accessibility are particularly attractive for EDLC-type MSCs [45,46,47].

Carbon-based materials have therefore played a central role in the development of MSCs. Activated carbon, graphene, reduced graphene oxide, carbon nanotubes, carbon nanofibers, porous carbon, carbon aerogels, and laser-induced graphene offer different combinations of conductivity, surface area, pore structure, flexibility, and chemical stability [48]. However, a high nominal surface area does not necessarily translate into proportionally high capacitance. Only the fraction of the surface that is accessible to electrolyte ions and electrically connected to the current collector contributes effectively to charge storage. This distinction becomes particularly important in MSC electrodes because the dimensions of pores, electrode thickness, and electrolyte composition can determine whether ions can rapidly penetrate and utilize the internal surface. Very small pores can provide substantial surface area, but ion accessibility may become restricted depending on the solvated ion size and electrolyte environment. Conversely, excessively large pores may facilitate ion transport but provide less surface area for charge accumulation. Therefore, the optimization of pore-size distribution rather than simply maximizing surface area is an important principle for high-performance MSC design. Pseudocapacitive MSCs employ electrochemically active materials capable of rapid and reversible Faradaic reactions [49,50]. In contrast to purely electrostatic EDLC storage, pseudocapacitance involves electron transfer accompanied by changes in the oxidation state or chemical environment of electroactive species. Conducting polymers such as polyaniline, polypyrrole, and poly(3,4-ethylenedioxythiophene), together with transition-metal oxides such as manganese dioxide, ruthenium oxide, nickel oxide, cobalt oxide, vanadium oxide, and molybdenum oxide, have been extensively investigated for this purpose. Pseudocapacitive materials can provide substantially higher capacitance because charge storage is not restricted exclusively to electrostatic ion adsorption. However, Faradaic processes may also induce structural changes, ion insertion, swelling, contraction, dissolution, and mechanical degradation during repeated cycling. These limitations are particularly relevant to wearable MSCs, where electrochemical cycling occurs simultaneously with mechanical deformation. Thus, the apparent advantage of high pseudocapacitance must be evaluated against the requirements for long-term structural and electrochemical stability. The distinction between EDLC and pseudocapacitive behavior is increasingly becoming less absolute because many advanced MSC electrodes exhibit mixed charge-storage mechanisms. Hybrid electrodes combine highly conductive electric-double-layer materials with Faradaically active components to exploit the complementary advantages of both mechanisms [51,52,53]. For example, porous carbon frameworks can provide rapid electron transport and structural support, while transition-metal compounds or conducting polymers introduce additional redox-active sites. Similarly, two-dimensional materials such as MXenes can simultaneously contribute surface-controlled charge storage, redox activity, high electrical conductivity, and ion-accessible layered structures. These hybrid systems are particularly attractive because they can potentially overcome the intrinsic limitations of individual material classes. Nevertheless, the successful construction of a hybrid electrode requires careful control over component distribution, interfacial contact, porosity, mass loading, and mechanical compatibility. Simply combining two high-performing materials does not necessarily produce a high-performing device; the interfaces between the components can become additional barriers to charge and ion transport.

The electrochemical response of MSCs is commonly evaluated through cyclic voltammetry (CV), galvanostatic charge–discharge (GCD), and electrochemical impedance spectroscopy (EIS) [54,55]. In CV measurements, the potential is continuously swept over a defined voltage range, and the resulting current response provides information regarding the reversibility and kinetics of charge storage. An ideal EDLC generally produces a nearly rectangular CV profile because the current associated with capacitive charging remains relatively constant during potential scanning. Pseudocapacitive materials, in contrast, often exhibit broad redox features resulting from Faradaic reactions. However, the shape of the CV curve can be strongly influenced by internal resistance and ion-transport limitations, particularly at high scan rates. An MSC exhibiting a nearly rectangular CV curve over a broad range of scan rates generally indicates rapid charge propagation and effective electrolyte accessibility, whereas severe distortion at increasing scan rates suggests increasing kinetic limitations. GCD measurements provide a complementary method for determining capacitance and evaluating charge-discharge reversibility. An ideal EDLC exhibits an approximately triangular and symmetric charge–discharge profile, whereas deviations from linearity can arise from pseudocapacitive reactions, internal resistance, ion diffusion, or complex electrode structures. For MSCs, particular attention should be paid to the instantaneous voltage drop at the beginning of discharge, commonly referred to as the IR drop. This feature reflects the combined resistance of the electrode, electrolyte, current collector, substrate, interfaces, and electrical connections. Because these resistive contributions can become significant at the microscale, minimizing them is essential for achieving high power density. EIS provides deeper insight into the kinetic and resistive characteristics of MSCs by probing the response of the device over a broad frequency range. The high-frequency intercept of a Nyquist plot is commonly associated with the equivalent series resistance, while the intermediate- and low-frequency regions provide information about charge-transfer processes, ion diffusion, and capacitive behavior. The equivalent series resistance arises from multiple sources, including ionic resistance of the electrolyte, electronic resistance of the electrode, resistance of the current collector, contact resistance, and interfacial resistance between different components. A low resistance is particularly important for high-power operation because resistive losses increase rapidly under high-current conditions. The characteristic response time of an MSC can be approximately described by the RC time constant. A smaller time constant generally indicates faster energy-storage response and is highly desirable for pulsed-power applications such as wireless sensors, wearable electronics, and microscale communication systems [56,57].

One of the defining characteristics of MSCs is their electrode architecture. Conventional supercapacitors frequently employ stacked electrodes separated by porous membranes, whereas planar MSCs often use interdigitated electrode configurations in which positive and negative electrodes are patterned laterally on the same substrate. The interdigitated geometry is particularly attractive for microelectronics because it eliminates the need for a conventional separator and reduces the characteristic distance over which ions must travel between the electrodes. The finger width, electrode length, electrode thickness, number of fingers, and interelectrode gap can therefore strongly influence device performance. Reducing the electrode gap can shorten ionic transport pathways and improve high-rate performance, but excessively narrow gaps increase fabrication complexity and can raise the risk of electrical short circuits or leakage. Likewise, increasing electrode thickness can increase active-material loading and areal capacitance, but excessively thick electrodes may develop concentration gradients, incomplete electrolyte penetration, increased ionic resistance, and mechanical instability. Consequently, electrode geometry must be optimized as an integrated electrochemical and manufacturing parameter rather than treated simply as a patterning variable. The relationship between characteristic diffusion length and ion-transport kinetics provides an important theoretical basis for the advantages of microscale architectures [58,59,60]. The porous networks contain interconnected micropores, mesopores, and macropores with different transport characteristics. Micropores can provide extensive electrochemically active surface area, mesopores can facilitate ion movement toward internal surfaces, and macropores can serve as electrolyte reservoirs and transport channels. Accordingly, hierarchical pore structures are increasingly considered desirable for simultaneously achieving high capacitance and rapid charge propagation. The operating voltage of an MSC is another fundamental determinant of its energy-storage capability. The energy stored in an ideal capacitor depends on both capacitance and the square of the operating voltage. This relationship explains why electrolyte engineering is critically important in the development of high-energy MSCs. Aqueous electrolytes generally provide high ionic conductivity, low cost, and favorable safety characteristics but typically possess a relatively limited electrochemical stability window. Organic electrolytes and ionic liquids can offer wider operating voltages, potentially increasing energy density, although they may introduce disadvantages related to ionic conductivity, cost, viscosity, flammability, or processing. For flexible and wearable MSCs, gel polymer electrolytes, hydrogels, ionogels, and solid-state electrolytes are particularly attractive because they can provide ionic conductivity while simultaneously improving mechanical integrity and reducing leakage. The electrolyte therefore functions not merely as an ion-conducting medium but as an integral component of the electrochemical and mechanical architecture.

The power capability of an MSC is closely related to its internal resistance and operating voltage [61,62,63]. Highly conductive electrode materials, intimate electrode-current collector contact, optimized electrode geometry, high-conductivity electrolytes, and short ion-transport pathways can collectively reduce resistive losses. Nevertheless, increasing the active-material loading may introduce additional diffusion resistance. This creates a fundamental trade-off in MSC engineering: thin and highly porous electrodes can provide excellent rate performance but limited energy storage, whereas thick electrodes can provide greater energy density but potentially poorer power capability. The objective is therefore to establish a balanced architecture in which sufficient active material is incorporated without creating substantial ionic or electronic transport limitations. The distinction between planar and three-dimensional architectures is particularly important in addressing this energy-power trade-off. Planar interdigitated MSCs offer excellent integration and short diffusion distances but may suffer from relatively low active-material loading per footprint. Three-dimensional electrodes, vertically aligned nanostructures, porous frameworks, micro-pillars, and multilayer architectures can increase the quantity of active material while maintaining relatively efficient ion transport. The development of three-dimensional architectures therefore represents an important strategy for improving volumetric and areal energy density without sacrificing the fundamental advantages of microscale transport. However, the fabrication of complex three-dimensional structures introduces additional challenges involving mechanical stability, uniform electrolyte penetration, pattern fidelity, current-collector integration, and scalable manufacturing. The substrate and current collector also play a disproportionately important role in MSCs compared with conventional bulk supercapacitors. Silicon, glass, metals, polyimide, polyethylene terephthalate, elastomers, paper, textiles, and other substrates have been explored depending on the intended application. For wearable electronics, substrates must possess adequate flexibility, dimensional stability, and compatibility with the active electrode and electrolyte. Current collectors must provide efficient electron transport while maintaining strong adhesion to the active material. Poor interfacial adhesion can increase resistance and cause delamination during repeated bending or stretching. In this respect, the current collector should not be regarded simply as an electrically conductive support; its morphology, conductivity, adhesion, surface chemistry, and mechanical compatibility can directly influence the long-term performance of the complete MSC [64,65,66]. Mechanical behavior becomes particularly important when MSCs are intended for wearable electronics. A wearable MSC must maintain electrochemical functionality while experiencing bending, folding, twisting, stretching, compression, or combinations of these deformation modes. Mechanical deformation can alter conductive pathways, generate cracks, cause delamination, disturb electrode–electrolyte interfaces, and accelerate electrolyte loss. Consequently, mechanical flexibility must be integrated into the original device design rather than introduced after electrochemical optimization. Flexible substrates, porous electrodes, elastomeric binders, serpentine conductors, wrinkled structures, kirigami and origami architectures, fiber-shaped devices, and self-healing materials have emerged as strategies for accommodating mechanical deformation. Importantly, the term “flexible MSC” should represent more than the ability of a device to survive a single bending event; meaningful assessment requires electrochemical characterization before and after repeated mechanical deformation and, ideally, simultaneous mechanical and electrochemical cycling. Another fundamental consideration is the distinction between intrinsic material performance and complete-device performance. Electrode materials are frequently evaluated using three-electrode configurations, where a small quantity of active material is deposited on a conductive substrate and characterized under optimized laboratory conditions. Such measurements are useful for understanding intrinsic electrochemical behavior but cannot be directly equated with the performance of a complete two-electrode MSC. In a practical device, the performance is influenced by the masses and volumes of both electrodes, electrolyte, current collectors, substrates, encapsulation layers, interconnections, and inactive components. This distinction is especially important because MSCs often contain extremely small quantities of active material. Consequently, a very high gravimetric capacitance may coexist with relatively modest areal or volumetric performance at the complete-device level. For wearable electronics, areal and volumetric metrics can therefore provide more meaningful indicators of practical performance than gravimetric values alone [67,68,69].

The fundamental electrochemical and architectural relationships described above establish the basis for understanding high-performance MSCs. An ideal device should simultaneously provide high electrochemically accessible surface area, efficient electronic conductivity, rapid ion transport, suitable operating voltage, low equivalent series resistance, high active-material utilization, and stable electrode–electrolyte interfaces [70,71,72]. At the same time, wearable applications impose additional requirements for flexibility, stretchability, fatigue resistance, environmental stability, safety, and scalable fabrication. These requirements are intrinsically interconnected. Increasing electrode loading can improve energy density but may compromise rate capability; introducing highly active pseudocapacitive materials can increase capacitance but potentially reduce cycling stability; increasing porosity can improve ion accessibility but decrease volumetric density; and incorporating flexible polymers can improve mechanical compliance while increasing resistance. Therefore, the design of high-performance MSCs is fundamentally a multivariable optimization problem rather than a single-material optimization problem. The fundamentals of MSC technology can be understood through the interconnected sequence of charge-storage mechanism, electrode chemistry, microstructure, ion and electron transport, device geometry, electrolyte properties, mechanical architecture, and integration strategy. The microscale dimensions of MSCs provide an intrinsic advantage in transport kinetics and enable direct integration with emerging electronic systems, but they also make device performance increasingly sensitive to interfaces, fabrication precision, active-material utilization, and parasitic components. As a result, the next generation of MSCs will require coordinated engineering across multiple length scales, from atomic-level surface chemistry and nanoscale porosity to microscale electrode patterning and macroscale wearable integration. This materials-to-device perspective provides the fundamental framework for critically assessing the advanced electrode materials, electrolytes, microfabrication technologies, architectures, and wearable applications discussed in the subsequent Sections of this review.

## 3. Performance Metrics and Evaluation Standards

The performance evaluation of microsupercapacitors (MSCs) requires a more rigorous framework than that commonly applied to conventional supercapacitors because the miniaturized dimensions, extremely low active-material mass, planar geometries, and strong dependence on fabrication parameters can substantially influence the measured electrochemical response [73,74,75]. In particular, the growing diversity of MSC architectures-including in-plane interdigitated, sandwich-type, vertical, three-dimensional, fiber-shaped, flexible, and stretchable configurations-has resulted in considerable variation in the way capacitance, energy density, power density, and cycling stability are reported. Although exceptionally high values are frequently highlighted to demonstrate progress, direct comparison between different studies can be misleading when different normalization methods, electrode areas, active-material masses, voltage windows, or measurement configurations are employed. Therefore, meaningful assessment of MSC technology requires simultaneous consideration of electrochemical performance, dimensional characteristics, mechanical durability, rate capability, operational stability, and device-level functionality. For wearable electronics in particular, the most meaningful performance indicator is not necessarily the highest gravimetric capacitance, but the extent to which an MSC can deliver useful energy and power within a constrained footprint while maintaining stable operation under repeated mechanical deformation.

### 3.1. Capacitance as a Fundamental Performance Parameter

Capacitance is the most frequently reported parameter for evaluating MSCs and represents the charge-storage capability of the device [76]. Capacitance may subsequently be normalized according to the mass of active material, electrode area, or device volume. Gravimetric capacitance, expressed in F g^−1^, is particularly useful for comparing the intrinsic charge-storage capability of different electrode materials. However, its relevance becomes more limited when the active material represents only a very small fraction of the complete MSC. Because wearable and integrated electronics are constrained primarily by available area and thickness, areal capacitance (F cm^−2^) and volumetric capacitance (F cm^−3^) are often more meaningful device-level metrics. A major concern in MSC literature is the inconsistent definition of the normalization basis. Some studies report capacitance based on the total mass of both electrodes, whereas others use only the active material on one electrode. Similarly, the geometric area may refer to the complete device area, active electrode area, interdigitated electrode area, or electrochemically accessible region. These differences can produce apparently large discrepancies even when the underlying devices have comparable performance. Therefore, the reporting methodology should clearly specify the normalization basis and distinguish between material-level capacitance and full-device capacitance. For asymmetric MSCs, the mass balancing between positive and negative electrodes is also critical because the charge stored by the two electrodes must satisfy (q_+ = q_−). Failure to properly balance electrode masses can result in underutilization of one electrode and misleading device performance.

### 3.2. Areal and Volumetric Capacitance

Areal capacitance is particularly important for microscale energy-storage systems because the physical footprint of the device is one of the primary constraints in wearable and on-chip applications. It can be calculated by normalizing the capacitance to the projected device area. Higher areal capacitance allows an MSC to store more charge without increasing its footprint and is therefore desirable for densely integrated electronics. However, areal capacitance should always be accompanied by information about electrode thickness and active-material loading. An ultrathin electrode may exhibit excellent rate performance and relatively high normalized capacitance but may contain insufficient active material to provide meaningful energy at the device level. Volumetric capacitance provides complementary information by considering the device volume. This parameter is particularly relevant for compact wearable electronics where both footprint and thickness are restricted. Three-dimensional electrode architectures can substantially improve volumetric performance by increasing the amount of electrochemically active material within a limited volume. Nevertheless, volumetric normalization can also become ambiguous when researchers use only the volume of the active material rather than the complete device volume. A consistent definition should therefore distinguish between active-material volumetric capacitance and full-device volumetric capacitance [77]. For meaningful comparison of MSCs, reported electrochemical performance should preferably be accompanied by areal and volumetric metrics in addition to gravimetric values. The normalization basis, active-material loading, electrode dimensions, operating voltage, and inclusion or exclusion of current collectors and substrates should also be specified. Where sufficient information is available, device-level comparisons based on areal capacitance, areal energy density, power density, and volumetric performance provide a more realistic assessment for wearable and on-chip applications. However, direct recalculation should be avoided when essential experimental parameters are not reported, as this may introduce additional uncertainty.

### 3.3. Energy Density

Energy density determines how much energy an MSC can store and is therefore one of the most important indicators of its practical usefulness. This relationship demonstrates that energy density depends not only on capacitance but also strongly on operating voltage. Because energy scales with the square of voltage, expanding the stable electrochemical voltage window can be highly effective for improving energy density. This explains the increasing interest in organic electrolytes, ionic liquids, gel electrolytes, and solid-state electrolytes with broader electrochemical stability windows. For wearable and microelectronic applications, areal and volumetric energy densities are particularly informative because they reflect the energy available within the constrained dimensions of the integrated device. However, comparisons must account for the normalization basis. Reporting an extremely high gravimetric energy density based solely on the mass of a nanostructured active material may not accurately represent the practical energy density of the complete MSC, particularly when substrate, current collector, electrolyte, encapsulation, and inactive structural components contribute substantially to the total device mass [78].

The comparison Table 1 demonstrates that MSC performance varies substantially with device architecture, operating voltage, active-material loading, electrolyte, testing current density, and normalization basis. Therefore, the reported values should not be interpreted as a direct ranking of device performance. Only parameters explicitly reported for the corresponding device configuration are included, while unavailable or non-comparable parameters are denoted as NR. The comparison in Table 1 also illustrates why reported MSC performance cannot be interpreted solely from a single electrochemical metric. Differences in voltage window, active-material loading, device geometry, electrolyte, normalization basis, and testing conditions can substantially influence the reported values. Therefore, areal and volumetric metrics are particularly useful for evaluating practical microscale devices, but they should be considered together with device thickness, loading, cycling stability, and mechanical reliability.

### 3.4. Power Density

Power density represents the rate at which stored energy can be delivered. One of the principal advantages of MSCs over many other energy-storage technologies is their ability to provide rapid charge and discharge. High capacitance alone does not guarantee high power performance. An MSC with a highly capacitive electrode but poor electronic conductivity, inefficient current collection, or slow ionic transport may exhibit substantial resistance and therefore limited power output. The energy density (E_d_) and power density (P_d_) of the device were calculated using the following relations (1) and (2),

E_d_ = ½ CV^2^, (1)

P_d_ = E/∆t, (2)

where C represents the device capacitance, V is the operating voltage window, and Δt is the discharge time. For wearable devices, areal power density can be particularly useful because it indicates the amount of power available from a defined device footprint. However, the reported value should be accompanied by the discharge current, voltage range, and normalization method to allow meaningful comparison [79].

**Table 1:** Representative electrochemical and mechanical performance parameters reported for microsupercapacitor electrodes and devices.

**S. No.**	**MSC Electrode/Architecture**	**Device Configuration**	Voltage Window (V)	Mass Loading (mg cm^−2^)	Device Thickness	Areal Capacitance (mF cm^−2^)	Volumetric Capacitance (F cm^−3^)	Areal Energy Density (μWh cm^−2^)	Areal Power Density (μW cm^−2^)	Mechanical Testing	Cycling Stability	Ref.
1	Laser-patterned Ti_3_C_2_T_x_ MXene	Planar, metal-free solid-state MSC	NR	3.0	NR	138	NR	6.85	NR	NR	95% after 10,000 cycles	[52]
2	Graphene/EGaIn	Deformable planar MSC	NR	NR	NR	1.336	NR	NR	NR	20% stretching/folding, 1000 cycles	~90% after 1000 deformation cycles	[71]
3	Fe_2_O_3_/graphene/Ag	3D-printed solid-state MSC	1.3	NR	NR	412.3	NR	65.4	1070	90.2% retention after 500 bending cycles	89% after 5000 cycles	[80]
4	Porous TiN	Planar interdigitated MSC	1.8	NR	NR	NR	NR	0.46	703.12	NR	88.2% after 10,000 cycles	[81]
5	CDs/c-CNTs/Ti_3_C_2_	Flexible solid-state planar MSC	NR	NR	NR	1162.6	NR	11.1	NR	NR	102.1% after 8000 cycles	[82]
6	Ni–Mo–S/Ti_3_C_2_T_x_ on laser-scribed graphene	Flexible planar MSC	NR	NR	NR	208	NR	65.10	4212	Flexible under bending/twisting; quantitative deformation condition NR	94.6% after 10,000 cycles	[83]

NR = Not Reported.

### 3.5. Rate Capability and High-Rate Performance

Rate capability describes the ability of an MSC to maintain its charge-storage characteristics as the charge-discharge rate increases. This is particularly important for wearable electronics because many microsystems operate intermittently and require rapid delivery of power. Rate performance can be evaluated using CV at progressively increasing scan rates or GCD at increasing current densities. An MSC with excellent rate capability should maintain a large fraction of its capacitance at high current densities or scan rates. This behavior is strongly influenced by electrode thickness, pore-size distribution, electrical conductivity, electrolyte ionic conductivity, and interelectrode distance. Planar MSCs can possess a significant rate advantage because the short lateral diffusion distance reduces ion-transport limitations. However, excessively high scan rates may cause the electrochemical response to become dominated by the outer surface of the electrode, resulting in incomplete utilization of deeper active material. Consequently, rate capability should not be interpreted as an isolated numerical value. It should be evaluated together with the electrode loading and device architecture. Reporting capacitance retention over a broad range of current densities provides a more meaningful representation of high-rate behavior than reporting performance at only one exceptionally high scan rate [80].

### 3.6. Equivalent Series Resistance and Internal Resistance

Equivalent series resistance is a critical parameter governing both power capability and energy efficiency. ESR arises from multiple contributions, including electrode resistance, electrolyte resistance, current-collector resistance, contact resistance, substrate resistance, and interfacial resistance [84]. It can be estimated from the instantaneous voltage drop in a GCD curve or determined more systematically through electrochemical impedance spectroscopy. For an MSC, low ESR is especially important because the resistance of microscale current collectors and interconnections can become significant relative to the total device resistance. Increasing the electrical conductivity of the electrode, improving contact between active material and current collector, reducing electrolyte resistance, and optimizing electrode geometry are therefore essential strategies for reducing ESR. A small time constant indicates rapid charging and discharging and is particularly desirable for high-frequency or pulsed-power applications.

### 3.7. Cycling Stability and Capacitance Retention

Long-term cycling stability is one of the major advantages of supercapacitors compared with many battery technologies [81,84]. MSCs are generally expected to retain a high proportion of their initial capacitance after thousands or tens of thousands of charge-discharge cycles. However, cycling stability should be evaluated under realistic current densities and voltage windows. Although high retention is desirable, the number of cycles alone does not establish practical durability. The degradation mechanism should also be considered. Conducting polymers may undergo swelling and contraction during repeated redox processes, while metal oxides may experience structural changes or dissolution. Carbon-based electrodes generally exhibit excellent cycling stability, but mechanical delamination, electrolyte evaporation, or substrate degradation can still cause device-level failure. For wearable MSCs, electrochemical cycling should ideally be coupled with mechanical cycling, because the device may experience thousands of bending or stretching events during actual use. Reporting electrochemical stability before and after repeated mechanical deformation therefore provides substantially more useful information than conventional cycling data alone.

### 3.8. Self-Discharge and Energy Retention

Self-discharge describes the spontaneous loss of stored charge when an MSC is disconnected from an external circuit. Although supercapacitors can generally deliver high power, their self-discharge can limit long-term energy retention. Self-discharge may originate from leakage currents, parasitic Faradaic reactions, electrolyte impurities, electrode asymmetry, and redistribution of ions within porous electrodes. For wearable sensors that may operate intermittently, excessive self-discharge can reduce the availability of stored energy between operating cycles. Therefore, voltage retention over time should be reported under controlled open-circuit conditions. The measurement duration, initial voltage, electrolyte composition, temperature, and device architecture should be clearly specified [82].

### 3.9. Coulombic Efficiency and Energy Efficiency

Coulombic efficiency provides information about the reversibility of charge storage and can be estimated from the ratio of discharge time or charge passed during discharge to that during charging under comparable conditions. High coulombic efficiency indicates highly reversible charge-storage behavior. Energy efficiency is also important because energy losses can arise from ESR and irreversible electrochemical reactions. In practical wearable systems, repeated energy conversion losses can significantly influence overall system efficiency. Therefore, high capacitance and high coulombic efficiency should ideally be accompanied by low resistance and high energy efficiency.

### 3.10. Mechanical Performance for Wearable MSCs

Mechanical stability has become a defining performance criterion for wearable MSCs. Depending on the intended application, devices may be evaluated under bending, folding, twisting, stretching, compression, or repeated mechanical cycling. A useful mechanical evaluation should report the deformation mode, bending radius or tensile strain, number of deformation cycles, and electrochemical performance before, during, and after deformation. For flexible MSCs, capacitance retention after repeated bending is commonly used as an indicator of structural stability. Stretchable MSCs require more stringent characterization because the electrodes and conductive pathways must accommodate substantial tensile strain. Advanced designs employ serpentine conductors, wrinkled electrodes, elastomeric substrates, porous networks, and self-healing materials to maintain electrical continuity. Importantly, a device should not be described as “flexible” merely because it can be bent once without catastrophic failure. For wearable applications, flexibility should be evaluated in terms of reversible mechanical deformation and long-term electrochemical stability under repeated deformation.

### 3.11. Ragone Analysis

The Ragone plot provides a convenient graphical representation of the trade-off between energy density and power density. For MSCs, it can reveal whether improvements in energy storage are achieved at the expense of power capability. However, Ragone comparisons are meaningful only when the same normalization basis is used. A rigorous Ragone analysis should distinguish among gravimetric, areal, and volumetric values and clearly identify whether the data correspond to electrode-level or complete-device measurements. In the context of wearable electronics, an additional dimension-mechanical durability-would be highly valuable. Future reporting could therefore consider three-dimensional performance maps incorporating energy density, power density, and mechanical durability, thereby providing a more realistic representation of wearable MSC performance.

### 3.12. Toward Standardized Evaluation of MSCs

The rapid expansion of MSC research has created a need for more consistent reporting standards. At minimum, researchers should clearly report electrode dimensions, active-material loading, device area, device volume, substrate, current collector, electrolyte composition, voltage window, testing configuration, scan rate or current density, and normalization method. Full-device values should be distinguished explicitly from three-electrode material-level measurements. A particularly important recommendation is that record performance should not be evaluated independently of active-material loading and device architecture. A device containing an ultralow mass of active material may exhibit an exceptional gravimetric value while storing very little absolute energy. Conversely, a thicker electrode may provide substantially higher areal energy but suffer from poorer rate capability. Therefore, the most meaningful evaluation considers multiple complementary metrics rather than identifying a single “best” MSC. Ultimately, the performance of an MSC for next-generation wearable electronics should be assessed through a multidimensional framework encompassing capacitance, energy density, power density, ESR, rate capability, cycling stability, self-discharge, mechanical durability, safety, scalability, and system-level functionality. Such a framework moves the field beyond the pursuit of isolated record values and toward realistic assessment of technological readiness. In particular, future studies should increasingly emphasize full-device measurements, standardized normalization, high active-material loading, mechanical cycling, long-term environmental stability, and operation within integrated wearable systems. Establishing consistent evaluation practices will be essential for identifying genuinely transformative MSC technologies and for accelerating their transition from laboratory-scale demonstrations to reliable, scalable, and commercially relevant wearable energy-storage platforms.

## 4. Advanced Electrode Materials for High-Performance MSCs

The electrode is the principal electrochemically active component of a microsupercapacitor (MSC) and largely determines its capacitance, rate capability, operating voltage, energy density, power density, cycling stability, and mechanical reliability [85,86,87]. Unlike conventional supercapacitors, in which relatively large quantities of active material can be incorporated into porous electrodes, MSCs operate under stringent geometric and mass constraints. Consequently, electrode materials for MSCs must simultaneously provide high electrochemically accessible surface area, rapid electron and ion transport, high active-material utilization, low electrical resistance, structural integrity at small dimensions, and compatibility with microfabrication processes. For wearable electronics, these requirements become even more demanding because the electrode must additionally tolerate repeated bending, folding, stretching, twisting, and environmental exposure. Thus, the development of MSC electrodes has evolved from the simple pursuit of high specific capacitance toward the rational design of multifunctional architectures in which composition, crystal structure, surface chemistry, porosity, conductivity, interfacial interactions, and mechanical properties are simultaneously controlled. From an electrochemical perspective, MSC electrode materials can be broadly divided into electric-double-layer-capacitive materials, pseudocapacitive materials, and hybrid or composite systems. Carbonaceous materials predominantly store charge through electrostatic ion adsorption, whereas transition-metal compounds and conducting polymers rely substantially on reversible Faradaic reactions. Two-dimensional materials such as graphene and MXenes can exhibit mixed surface-controlled and redox contributions depending on their composition and surface termination. The distinction among these categories is useful for understanding charge-storage mechanisms, but practical MSC electrodes increasingly combine multiple classes of materials to overcome their individual limitations. The central challenge is therefore not to identify a universally superior electrode material but to engineer a structure in which electronic conductivity, ionic accessibility, electrochemical activity, and mechanical integrity are mutually optimized [88,89,90].

### 4.1. Carbon-Based Electrode Materials

Carbon materials remain the foundation of MSC technology because of their high electrical conductivity, chemical stability, relatively low density, tunable porosity, and compatibility with diverse fabrication techniques. Their charge-storage mechanism is predominantly based on electric double-layer formation, although surface functional groups and heteroatom dopants can introduce additional pseudocapacitive contributions. The electrochemical performance of carbon electrodes is therefore determined not simply by total surface area but by the interplay among specific surface area, pore-size distribution, pore connectivity, electrical conductivity, surface chemistry, and electrolyte accessibility. Activated carbon is one of the most extensively studied carbon materials because its porous structure can be generated through physical or chemical activation of carbonaceous precursors. Micropores provide extensive surface area for ion adsorption, whereas mesopores facilitate ion transport into the electrode. However, excessive microporosity can become disadvantageous when the pore dimensions approach or fall below the effective size of solvated electrolyte ions. In such cases, ion desolvation and steric constraints may limit accessibility to nominally high-surface-area regions. Therefore, the optimization of activated-carbon MSCs increasingly focuses on hierarchical pore structures rather than maximum BET surface area alone. The incorporation of mesopores and macroporous channels can shorten ion-transport pathways and improve rate capability while retaining sufficient microporous surface area for charge storage [91].

Graphene represents another important carbon platform because its two-dimensional structure provides high electrical conductivity, large theoretical surface area, and excellent mechanical properties. Graphene-based MSCs can be fabricated through vacuum filtration, transfer processes, chemical vapor deposition, printing, electrophoretic deposition, and laser-based approaches. However, the strong van der Waals interactions between graphene sheets frequently lead to restacking, which reduces the accessible surface area and restricts electrolyte penetration. Consequently, structural strategies such as spacer incorporation, three-dimensional assembly, chemical functionalization, and hybridization with nanoparticles or polymers have been developed to maintain interlayer separation. The challenge is to preserve graphene’s high conductivity while preventing dense packing that compromises ionic transport. Reduced graphene oxide provides additional opportunities because oxygen-containing groups present in graphene oxide facilitate dispersion, solution processing, and interaction with other materials. Reduction partially restores electrical conductivity while retaining a degree of structural disorder and residual oxygen functionality. These surface functionalities can contribute modest pseudocapacitive behavior and provide nucleation sites for metal oxides, conducting polymers, and other electroactive phases. However, excessive reduction may decrease surface polarity and electrolyte wettability, whereas insufficient reduction can produce relatively high electronic resistance. Therefore, the reduction degree represents an important parameter for controlling the balance between conductivity, surface chemistry, and electrochemical accessibility [92].

Carbon nanotubes provide a complementary architecture characterized by excellent electrical conductivity, mechanical strength, and one-dimensional charge-transport pathways. Single-walled and multi-walled carbon nanotubes can form interconnected networks that improve electrical percolation within MSC electrodes. Their tubular morphology also provides mesoporous channels and can serve as a scaffold for pseudocapacitive materials. Nevertheless, nanotube bundles can limit electrolyte accessibility, and their relatively low intrinsic volumetric capacitance can restrict energy density when used alone. Hybridization with graphene, MXenes, conducting polymers, metal oxides, or other nanostructured materials can therefore increase electrochemical activity while maintaining the conductive network.

Carbon nanofibers and electrospun carbon fibers are particularly attractive for flexible MSCs because they can form mechanically robust, interconnected networks. Their one-dimensional morphology facilitates electron transport and can provide a continuous framework for deposition of active materials. Carbon nanofiber mats can also be integrated into textiles and fiber-shaped energy-storage devices. However, as with carbon nanotubes, the relatively limited intrinsic capacitance of graphitic carbon necessitates surface functionalization or incorporation of pseudocapacitive components when high energy density is required.

Laser-induced graphene (LIG) has emerged as a particularly important carbon platform for MSC fabrication because it combines material synthesis and microscale patterning in a relatively direct process. Laser irradiation of suitable carbon-containing polymeric substrates can generate porous graphene-like carbon structures with interconnected conductivity pathways. The ability to define interdigitated electrode patterns through localized laser processing makes LIG attractive for flexible and wearable MSCs. Its porous structure can provide high electrolyte accessibility, while the absence of conventional photolithography steps can simplify device fabrication. Nevertheless, LIG performance depends strongly on precursor chemistry, laser parameters, irradiation atmosphere, resulting carbon structure, and substrate properties. Further optimization is therefore required to achieve reproducible electrode properties across large areas.

Biomass-derived carbons represent an increasingly important direction for sustainable MSC electrodes. Agricultural residues, cellulose, lignin, chitosan, alginate, and other renewable precursors can be converted into porous carbon materials through carbonization and activation. These materials offer the possibility of combining low-cost feedstocks with tunable porosity and heteroatom-containing surface chemistry. Nitrogen, oxygen, sulfur, and phosphorus functionalities introduced either intrinsically or through post-treatment can modify wettability, electronic structure, and pseudocapacitive behavior. However, substantial variability in precursor composition and carbonization conditions can lead to batch-to-batch differences in porosity and conductivity. Therefore, the sustainability advantage of biomass-derived carbons must ultimately be evaluated together with reproducibility, processing energy, chemical activation requirements, and complete life-cycle considerations [93].

### 4.2. MXene-Based Electrode Materials

Two-dimensional transition-metal carbides, nitrides, and carbonitrides, collectively known as MXenes, have become prominent electrode materials for high-performance MSCs [94]. Their combination of metallic conductivity, layered morphology, hydrophilic surfaces, and tunable surface terminations provides a distinctive platform for rapid charge storage. Ti_3_C_2_T_x_ is the most extensively investigated MXene, where (T_x) represents surface terminations such as -O, -OH, and -F generated during synthesis and subsequent processing. These functional groups strongly influence interlayer spacing, hydrophilicity, ion adsorption, redox activity, and electronic structure. The layered architecture of MXenes facilitates rapid ion transport, particularly when the interlayer spacing is engineered appropriately. However, MXene sheets tend to restack during drying because of strong interlayer interactions, reducing the accessibility of internal surfaces. This problem is particularly serious in thick MSC electrodes where densely packed layers can develop substantial ion-transport resistance. Strategies to mitigate restacking include introducing carbon nanotubes, graphene, conductive polymers, nanoparticles, molecular spacers, and porous frameworks between MXene layers. Such structural modification can create open ion-transport channels while preserving the high electronic conductivity of MXenes. The surface chemistry of MXenes also provides opportunities for pseudocapacitive charge storage. Protonation, ion adsorption, and surface redox reactions can contribute to capacitance depending on the electrolyte and MXene composition. However, MXene oxidation remains a major challenge, particularly under aqueous and oxygen-rich environments. Surface oxidation can alter conductivity, structure, and electrochemical stability. For wearable applications, where devices may encounter humidity, temperature fluctuations, and mechanical stress, controlling MXene oxidation and developing effective encapsulation strategies will be essential. Beyond Ti_3_C_2_T_x_, the broader MXene family provides an extensive compositional space for tuning electronic and electrochemical properties. Nevertheless, differences in synthesis chemistry, surface termination, flake size, defect concentration, and oxidation state complicate direct comparison among reported materials. Consequently, future MSC studies should place greater emphasis on correlating MXene structure and surface chemistry with actual full-device performance rather than relying solely on nominal material composition.

### 4.3. Conducting Polymers

Conducting polymers provide an important route toward high-capacitance MSC electrodes because they combine electronic conductivity, mechanical compliance, and pseudocapacitive charge storage. Polyaniline, polypyrrole, and PEDOT-based materials have been particularly widely studied. Their charge-storage mechanism involves reversible oxidation and reduction accompanied by ionic compensation from the electrolyte. Because these reactions can involve a substantial number of electroactive sites, conducting polymers can achieve high capacitance relative to purely EDLC-based carbon electrodes. Polyaniline is attractive because of its relatively high theoretical capacitance, tunable oxidation states, and facile electrochemical synthesis. However, repeated protonation and deprotonation or ion insertion/extraction can induce significant volume changes, resulting in mechanical stress, cracking, and loss of electrical connectivity during long-term cycling. Polypyrrole exhibits similar advantages and limitations, with its electrochemical stability strongly dependent on synthesis conditions and electrolyte chemistry. PEDOT and PEDOT:PSS offer attractive electrical conductivity and mechanical flexibility and are particularly compatible with printed and flexible electronics. A common strategy for improving the cycling stability of conducting polymers is to confine them within a mechanically robust conductive scaffold. Carbon nanotubes, graphene, porous carbon, and MXenes can act as conductive backbones that accommodate polymer expansion while maintaining electrical pathways. The resulting composites can simultaneously provide EDLC and pseudocapacitive storage. However, excessive polymer deposition may block pores and hinder ion transport. Therefore, conformal and nanoscale polymer deposition is generally more advantageous than uncontrolled bulk polymer growth [95].

### 4.4. Transition–Metal Oxides

Transition-metal oxides provide rich redox chemistry and have therefore been widely investigated as pseudocapacitive electrode materials. Manganese dioxide is particularly attractive because of its relatively low cost, abundance, environmental compatibility compared with several noble-metal oxides, and multiple structural polymorphs. Its capacitance originates from surface redox processes and ion interactions with the oxide structure. However, its relatively low intrinsic electrical conductivity can limit rate capability. Integrating MnO_2_ with graphene, carbon nanotubes, porous carbon, or MXenes can improve electronic transport and increase the utilization of active sites. Ruthenium oxide is widely regarded as one of the benchmark pseudocapacitive materials because of its high theoretical capacitance, excellent electrical conductivity, and reversible redox behavior. Nevertheless, its high cost and limited abundance substantially restrict large-scale applications. Consequently, RuO_2_ is more valuable as a performance benchmark than as a likely dominant commercial material for wearable MSCs. Nickel oxide, cobalt oxide, nickel-cobalt oxides, vanadium oxides, and molybdenum oxides offer alternative redox chemistries and can exhibit high theoretical charge-storage capacities. Their practical performance, however, depends strongly on crystallinity, particle size, defect density, oxidation state, electrolyte compatibility, and electrode architecture. Nanostructuring can reduce diffusion distances and expose more active sites, but excessive nanoscale subdivision may increase interfacial instability or complicate scalable manufacturing. Thus, the challenge is to achieve sufficient nanoscale electrochemical activity without sacrificing structural integrity or manufacturability [83].

### 4.5. Transition-Metal Sulfides, Nitrides, Phosphides and Related Compounds

Beyond oxides, transition-metal sulfides, nitrides, phosphides, and selenides provide expanded opportunities for pseudocapacitive charge storage because their electronic structures and bonding environments can facilitate rapid redox processes. Materials such as MoS_2_, WS_2_, NiCo_2_S_4_, CoS_x_, Ni_3_S_2_, metal nitrides, and phosphides have been investigated because of their relatively high electrical conductivity and abundant redox-active sites. Two-dimensional sulfides can provide high surface-to-volume ratios and tunable interlayer spacing, while mixed-metal sulfides can introduce synergistic redox behavior and improved conductivity. Nevertheless, chemical stability and structural integrity remain important considerations. Some sulfide- and phosphide-based materials can undergo surface reconstruction or oxidation under electrochemical conditions, altering their active phase during cycling. Such transformations should not necessarily be regarded solely as degradation; in some systems, electrochemically reconstructed surfaces may become the actual active phase. Understanding these dynamic transformations is therefore essential for rational MSC design [96].

### 4.6. Metal–Organic Frameworks and Covalent Organic Frameworks

Metal–organic frameworks (MOFs) and covalent organic frameworks (COFs) offer an alternative approach based on molecularly controlled porosity and chemical functionality [97]. MOFs consist of metal nodes coordinated with organic ligands, generating highly ordered porous structures. Their tunable pore size, large internal surface area, and compositional flexibility make them attractive for electrochemical applications. However, many pristine MOFs possess relatively low electronic conductivity, limiting their direct use as high-power MSC electrodes. A particularly effective strategy is to convert MOFs into porous carbon, metal oxide, metal sulfide, or metal-carbon composites through controlled thermal treatment. MOF-derived materials can retain aspects of the precursor’s porous architecture while acquiring improved electrical conductivity and electrochemical activity. The ability to control metal distribution at the nanoscale can also promote uniform formation of electroactive sites. COFs provide complementary opportunities because their organic building blocks and covalent linkages allow systematic control of pore size, chemical functionality, and redox-active groups. Redox-active COFs can contribute pseudocapacitance while maintaining ordered ion-transport channels. However, electrical conductivity, chemical stability, synthesis complexity, and integration into microscale devices remain challenges. Their future importance may therefore depend on the development of conductive COFs and scalable synthesis methods compatible with microfabrication.

### 4.7. Two-Dimensional Materials Beyond Graphene and MXenes

The growing interest in two-dimensional materials has expanded the material landscape of MSC electrodes [98]. Transition-metal dichalcogenides, black phosphorus, layered metal oxides, and related materials offer tunable electronic structures, high surface areas, and potentially abundant active sites. MoS_2_ and WS_2_, for example, possess layered structures that can facilitate ion adsorption and surface redox processes. However, their relatively low intrinsic electrical conductivity compared with graphene and MXenes can restrict rate performance. Hybridization with conductive carbon frameworks or metallic phases can mitigate this limitation. Black phosphorus provides interesting electronic and structural properties but suffers from environmental instability, particularly oxidation under ambient conditions. Such instability presents a major challenge for practical wearable applications. The broader lesson from these materials is that exceptional intrinsic electrochemical characteristics must be balanced against chemical stability, processability, and device-level durability.

### 4.8. Metal–Organic and Polymer-Based Hybrid Electrodes

Hybridization has become one of the most powerful strategies for improving MSC performance because no single material simultaneously maximizes all desired characteristics. Carbon materials provide conductivity and structural stability, pseudocapacitive materials provide high charge-storage capacity, and polymers provide mechanical flexibility and interfacial compatibility. The design of hybrid electrodes therefore aims to establish complementary functions within a single architecture. For example, a porous carbon scaffold can serve as an electron-conducting framework, while MnO_2_ or a conducting polymer is deposited as a thin conformal layer. In such a configuration, the carbon prevents electrical isolation of the active phase, while the nanoscale thickness of the pseudocapacitive component minimizes ion-diffusion distances. Similarly, MXene/polymer composites can combine high electronic conductivity with flexible redox-active networks. The key requirement is to control the thickness and spatial distribution of each component. Excessive deposition can block pores and increase resistance, whereas insufficient loading may not provide meaningful enhancement in capacitance. Interfacial engineering is particularly important in these systems. Strong chemical bonding or favorable electronic coupling between components can facilitate charge transfer and reduce contact resistance. Heterointerfaces may also alter local electronic structures and generate additional electroactive sites. However, claims of “synergistic effects” should be supported by appropriate electrochemical, spectroscopic, and structural evidence rather than inferred solely from improved capacitance [99].

### 4.9. Hierarchical and Three-Dimensional Electrode Materials

The transition from two-dimensional films to hierarchical three-dimensional electrodes represents an important development in MSC technology. The primary objective is to increase active-material loading while maintaining efficient ion and electron transport. Three-dimensional porous carbon scaffolds, vertically aligned nanostructures, interconnected nanowire networks, and microstructured current collectors can provide large electrochemically accessible volumes within a small footprint. A well-designed hierarchical electrode should establish multiple transport pathways. Macroporous channels facilitate bulk electrolyte movement, mesopores support intermediate-scale ion diffusion, and micropores provide high surface area for charge storage. At the same time, a continuous electronic network should connect the electroactive domains to the current collector. Such architectures can alleviate the classical trade-off between high mass loading and poor rate capability. However, increasing structural complexity does not automatically improve performance. Three-dimensional electrodes may contain inaccessible regions, mechanically weak junctions, or poorly connected active phases. Furthermore, complex structures can be difficult to reproduce using scalable fabrication techniques. Therefore, the success of 3D MSC electrodes depends on whether their structural advantages can be maintained at practical manufacturing scales [100].

### 4.10. Heteroatom Doping and Defect Engineering

Heteroatom doping provides a molecular-level strategy for modifying carbon and other electrode materials. Nitrogen, oxygen, sulfur, phosphorus, boron, and combinations thereof can alter electronic density, surface polarity, wettability, defect concentration, and redox activity. Nitrogen-doped carbon, for example, can exhibit enhanced electrical conductivity and improved electrolyte affinity, while certain nitrogen configurations may contribute additional pseudocapacitive storage. Defect engineering provides another route for modifying the electronic and electrochemical properties of two-dimensional materials and carbon frameworks. Vacancies, edge sites, dislocations, and other defects can act as active sites for ion adsorption or redox reactions. However, excessive defect density may compromise structural stability or electrical conductivity. Therefore, defect concentration must be carefully optimized rather than maximized [101].

### 4.11. Electrode Materials for Flexible and Wearable MSCs

The requirements of wearable electronics fundamentally alter the criteria for electrode selection. High capacitance must be accompanied by mechanical compliance and strong interfacial adhesion. Traditional brittle inorganic films may fracture during repeated bending, whereas elastomer-supported nanocomposite networks can maintain electrical continuity under deformation. Conducting polymers, carbon nanotube networks, graphene frameworks, MXene-based composites, porous carbon fabrics, and fiber-shaped electrodes are particularly attractive because they can accommodate mechanical strain through distributed deformation. For stretchable MSCs, intrinsic material flexibility is not always sufficient. The overall electrode architecture must also accommodate strain. Wrinkled structures, porous networks, serpentine patterns, and kirigami-inspired geometries can convert macroscopic deformation into localized structural movement, thereby reducing strain on brittle active materials. This illustrates a fundamental principle of wearable MSC design: mechanical performance is a property of the electrode architecture as much as of the electrode material itself [102].

### 4.12. Critical Comparison of Electrode-Material Strategies

Despite remarkable advances, no existing electrode-material class simultaneously provides maximum capacitance, energy density, power density, cycling stability, flexibility, environmental stability, and manufacturing scalability [103,104,105]. Carbon materials remain attractive because of their stability and conductivity but are often limited by moderate capacitance. Conducting polymers and transition-metal compounds can deliver higher pseudocapacitance but may suffer from structural degradation and limited cycling stability. MXenes provide an attractive combination of conductivity, layered structure, and surface activity but face challenges associated with restacking and oxidation. MOF/COF-derived materials offer highly tunable architectures but require further development in conductivity and scalable processing. Hybrid materials can address several limitations simultaneously but introduce additional interfaces and synthesis complexity. The most promising strategy is therefore the development of hierarchically integrated composite electrodes in which each component performs a specific function. A conductive framework can provide electron transport and mechanical integrity; a pseudocapacitive phase can increase charge storage; a porous architecture can facilitate ion transport; and a flexible substrate can accommodate mechanical deformation. Such systems should be designed based on structure-property relationships rather than simply combining materials with individually high reported capacitance values. An important future direction is consequently the transition from “high-specific-capacitance materials” toward high-performance full-device electrodes with realistic active-material loading. For MSCs intended for wearable electronics, areal and volumetric capacitance, energy density, mechanical durability, and fabrication compatibility are likely to become increasingly important indicators. The electrode materials that ultimately achieve technological relevance will therefore be those capable of maintaining high electrochemical utilization at practical loading levels while surviving repeated deformation and being compatible with scalable microfabrication. In summary, electrode-material development has progressed from conventional porous carbons toward a diverse landscape encompassing two-dimensional materials, conducting polymers, transition-metal compounds, MOFs, COFs, biomass-derived carbons, heterostructures, and hierarchical nanocomposites. The fundamental challenge now lies in integrating these materials into architectures that simultaneously optimize ionic transport, electronic conductivity, electrochemical activity, mechanical resilience, and manufacturability. Future advances are expected to arise increasingly from interface engineering, multiscale structural design, heteroatom and defect control, high-loading porous architectures, sustainable material synthesis, and data-driven materials optimization, rather than from the isolated discovery of new compositions. Such developments will provide the materials foundation for the microfabrication strategies and device architectures required to translate high-performance MSCs into reliable energy-storage components for next-generation wearable electronics. The different electrode classes exhibit distinct advantages and limitations for MSC applications. Carbon-based electrodes generally provide good electrical conductivity, chemical stability, and rate capability, but their capacitance may be limited when charge storage relies primarily on the electrical double layer. MXenes offer high conductivity and pseudocapacitive charge storage, although oxidation, restacking, and environmental stability can restrict long-term performance. Conducting polymers can provide high pseudocapacitance and mechanical compatibility but may suffer from structural degradation during repeated cycling. Transition-metal oxides, sulfides, and selenides offer high redox activity but commonly face limitations associated with electrical conductivity, volume changes, and cycling stability. MOF- and COF-derived materials provide tunable porosity and compositional flexibility, although synthesis complexity, structural stability, and large-scale processing remain important considerations. Consequently, hybrid electrodes are increasingly investigated to balance conductivity, redox activity, ion accessibility, and mechanical durability rather than maximizing a single property.

### 4.13. Emerging Research on MSCs

Chen et al. developed a planar flexible microsupercapacitor (MSC) designed to simultaneously address the challenges of energy density, mechanical flexibility, cycling stability, and temperature tolerance in wearable energy-storage applications [51]. The device employs a self-assembled flower-like 1T-phase MoS_2_/reduced graphene oxide (rGO) hybrid as the electroactive electrode, in which the conductive rGO framework facilitates electron transport while the highly redox-active 1T-MoS_2_ component provides additional pseudocapacitive charge storage. This hybrid architecture is particularly advantageous because the two-dimensional MoS_2_/rGO configuration can provide abundant electrochemically accessible interfaces while mitigating the conductivity limitations commonly associated with transition-metal dichalcogenides. The authors further introduced a polyacrylamide (PAM)/trehalose/LiCl hydrogel electrolyte, where the interaction between PAM and trehalose contributes to the mechanical flexibility and environmental stability of the electrolyte. The resulting MSC exhibited a volumetric energy density of 15.0 mWh cm^−3^ and retained 88.21% of its initial capacitance after 8000 charge–discharge cycles at 20 °C, demonstrating a favorable combination of energy-storage capability and electrochemical durability.

**Figure 4:**
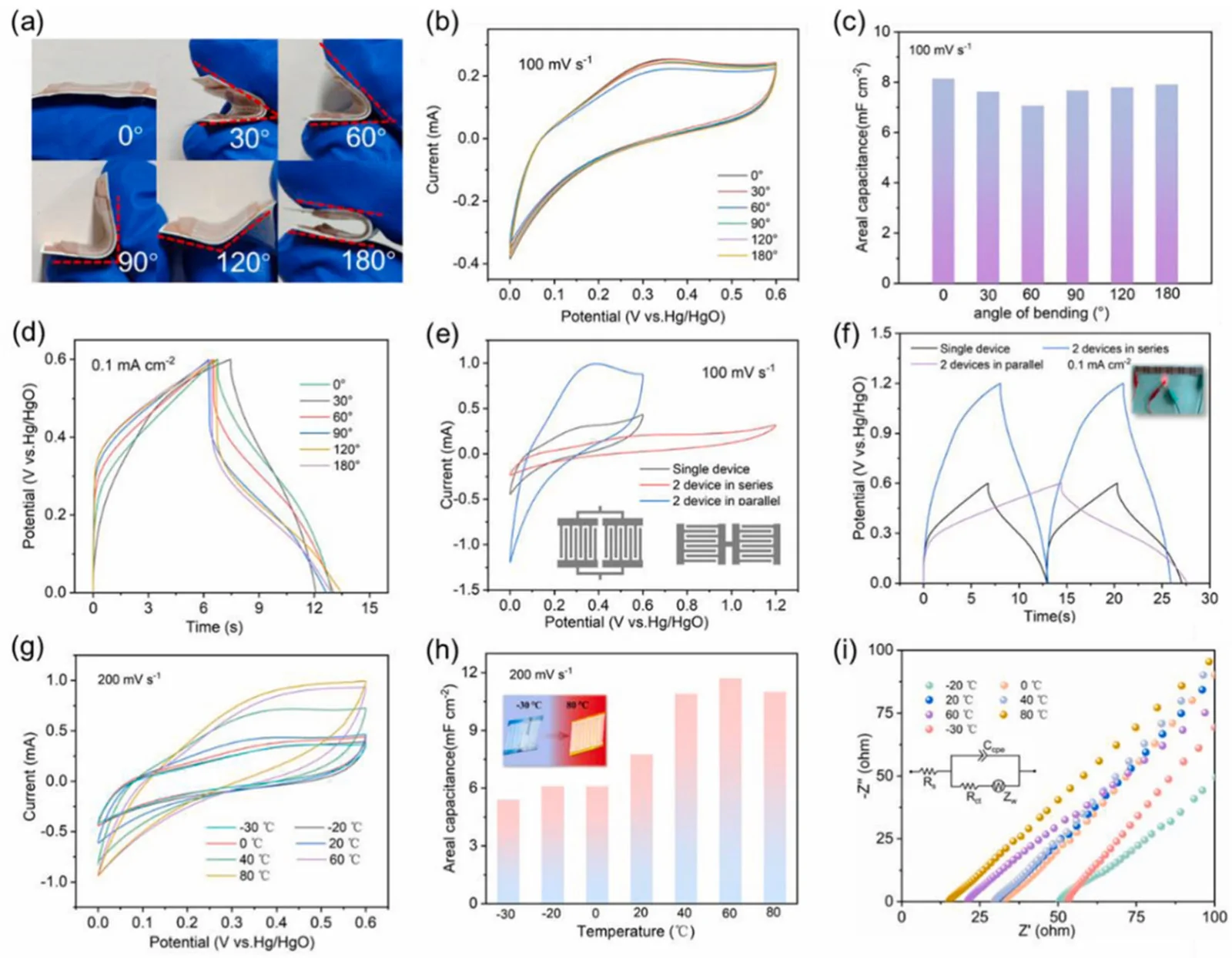
Flexibility, integration, and temperature-dependent performance of MSCs: (**a**) photographs at different bending angles; (**b**) CV curves; (**c**) GCD curves; and (**d**) areal capacitance under bending. (**e**) CV and (**f**) GCD profiles of single and series/parallel-connected devices. (**g**) CV curves, (**h**) areal capacitance, and (**i**) EIS spectra at different temperatures. Reproduced with permission from Ref. [51]. Copyright (2026) Elsevier.

Importantly, the PAM/trehalose/LiCl electrolyte enabled stable operation over a broad temperature range from −30 to 80 °C, addressing an important limitation of conventional hydrogel electrolytes that can suffer from freezing or dehydration under extreme conditions. The device also demonstrated excellent flexibility, highlighting its suitability for mechanically conformable electronics. Furthermore, the use of inkjet printing enabled convenient fabrication and electrical interconnection of multiple MSC units in series or parallel, providing a practical route toward increasing the output voltage and power of integrated microscale energy-storage systems. Overall, this work demonstrates that the combination of a conductive pseudocapacitive 1T-MoS_2_/rGO heterostructure with a mechanically and thermally robust hydrogel electrolyte provides an effective strategy for developing flexible MSCs capable of operating under diverse environmental conditions (Figure 4a–i). The study is particularly relevant to next-generation wearable electronics, where high volumetric energy density, mechanical compliance, temperature resilience, and scalable device integration must be achieved simultaneously.

**Figure 5:**
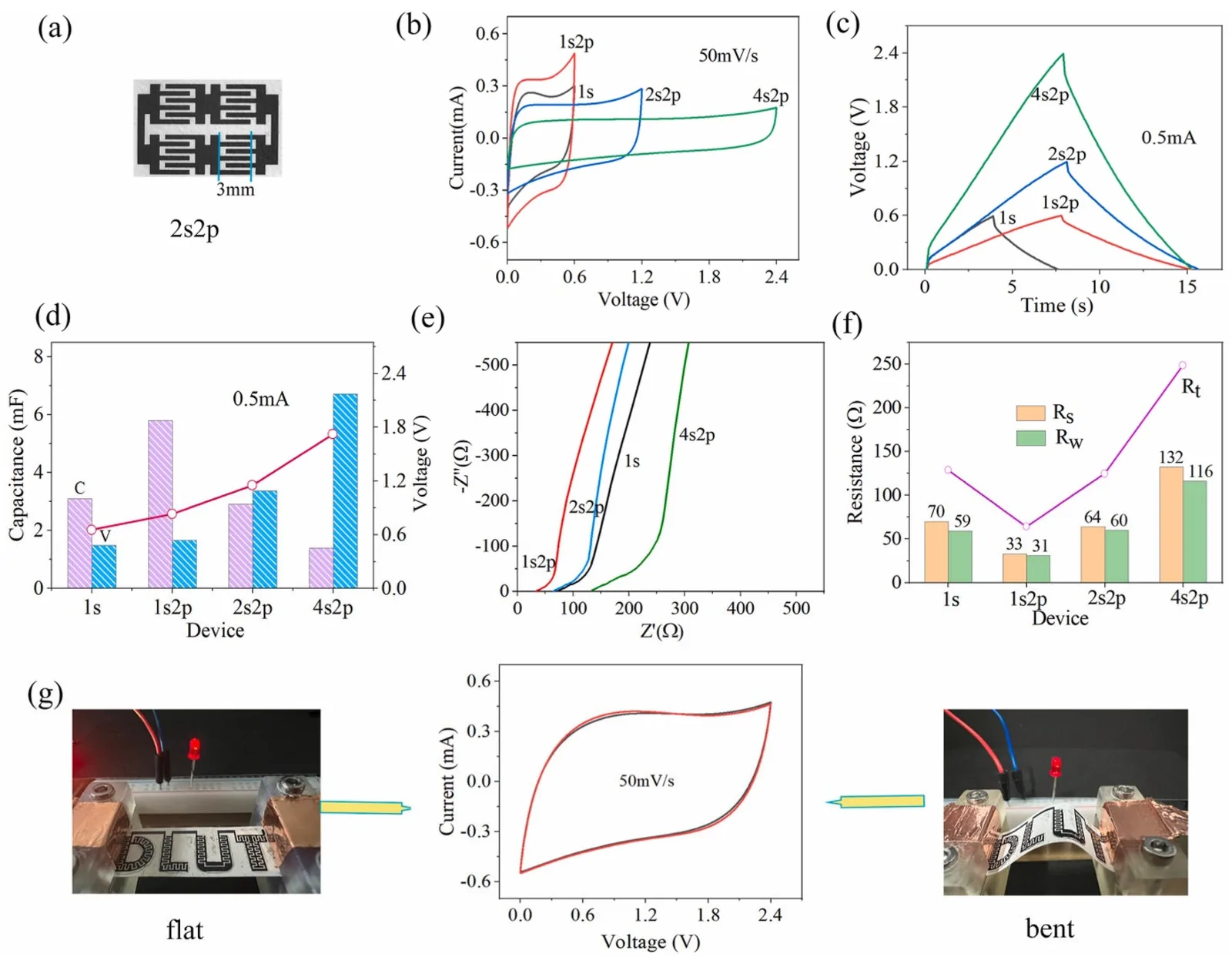
Device performance of integrated MXene-MSCs with different cell configurations (1s, 1s2p, 2s2p, and 4s2p): (**a**) digital photograph of a representative 2s2p integrated electrode; (**b**) CV curves at 50 mV/s; (**c**) GCD profiles at 0.5 mA; (**d**) capacitance and voltage variation; (**e**) EIS plots and (**f**) the corresponding resistances, and (**g**) a four-cell, letter-shaped “DLUT” MXene device powering an LED under flat and bent conditions, with nearly unchanged CV profiles. Reproduced with permission from Ref. [52]. Copyright (2025) Elsevier.

He et al. developed a high-performance, metal-free planar microsupercapacitor (MSC) using a water-cooling-assisted laser ablation (WALA) strategy to fabricate high-quality Ti_3_C_2_T_x_ MXene microelectrodes [52]. The study addresses an important limitation of conventional laser patterning of MXene films, namely, the localized thermal damage generated during laser processing, which can deteriorate electrode quality and simultaneously damage the underlying substrate. By performing laser ablation on a wet MXene membrane, evaporation of water effectively dissipates the heat generated during laser irradiation, enabling well-defined electrode patterns while substantially suppressing thermal degradation. This approach is particularly attractive for MSC fabrication because it is mask-free, facile, and compatible with customized electrode geometries and different substrates. The resulting MXene electrodes exhibited high electrical conductivity and were subsequently integrated with a poly(vinyl alcohol)/H_2_SO_4_ hydrogel electrolyte to construct metal-free solid-state planar MSCs. Importantly, the authors systematically investigated the influence of MXene areal mass loading on charge-storage performance, demonstrating that optimizing active-material loading is essential for balancing electrochemical accessibility and practical energy-storage capability. At an optimized MXene loading of 3 mg cm^−2^, the MSC achieved an areal specific capacitance of 138 mF cm^−2^ and an areal energy density of 6.85 μWh cm^−2^. The study also demonstrated that the laser-patterning strategy could generate diverse electrode configurations, shapes, and integrated architectures, highlighting its potential for scalable on-chip energy-storage manufacturing. The absence of metal current collectors further simplifies the device structure and contributes to a lightweight, compact configuration. From a broader perspective, this work demonstrates that the performance of MXene-based MSCs is governed not only by the intrinsic electrochemical properties of the two-dimensional electrode but also by processing-induced structural quality, electrode mass loading, and device architecture (Figure 5a–g). The WALA approach therefore provides an effective route for overcoming the thermal limitations of conventional laser fabrication while retaining the advantages of direct, mask-free patterning. This study is particularly relevant to next-generation wearable and flexible electronics because scalable laser processing can facilitate the fabrication of customized microscale energy-storage architectures without the complexity of conventional lithographic processing. More importantly, the work highlights the importance of integrating materials chemistry with manufacturing engineering to achieve practically relevant MSC performance.

## 5. Electrolytes and Electrode–Electrolyte Interfaces

Electrolytes are fundamental components of MSCs because they provide the mobile ionic species required for charge compensation, electric-double-layer formation, and, in many cases, reversible Faradaic reactions. Although electrode materials generally receive greater attention in MSC research, the electrolyte is equally important because it directly determines the operating voltage window, ionic conductivity, rate capability, energy density, self-discharge behavior, safety, environmental stability, and compatibility with flexible or wearable platforms [96]. At the microscale, the role of the electrolyte becomes even more pronounced because the electrode dimensions, narrow interelectrode spacing, confined geometries, and large electrode–electrolyte interfacial area can substantially alter ion transport and interfacial electrochemistry. Consequently, the design of high-performance MSCs requires simultaneous optimization of the electrode, electrolyte, and their interface rather than treating these components independently. In particular, the electrode–electrolyte interface governs the accessibility of electrochemically active sites, ion adsorption, desolvation, charge transfer, and the development of interfacial electric fields. Understanding these processes is therefore essential for designing MSCs capable of delivering high energy and power while maintaining long-term stability under mechanical and environmental stress. In a conventional MSC, the electrolyte serves primarily as an ion-conducting medium between the positive and negative electrodes. When an external voltage is applied, electrons move through the external circuit while ions migrate through the electrolyte toward oppositely charged electrode surfaces. In an EDLC, this ionic redistribution results in the formation of an electric double layer, whereas in pseudocapacitive systems the electrolyte additionally participates in charge-compensating Faradaic reactions. The ideal electrolyte should therefore exhibit high ionic conductivity, broad electrochemical stability, low viscosity, good electrode wettability, chemical compatibility with the electrode and current collector, low volatility, and sufficient thermal and mechanical stability [56,61]. However, these properties are rarely maximized simultaneously. For example, aqueous electrolytes typically provide excellent ionic conductivity and low resistance but a relatively narrow electrochemical stability window, whereas organic electrolytes and ionic liquids can provide wider operating voltages but may exhibit higher viscosity or lower ionic conductivity. This fundamental trade-off has driven extensive development of aqueous, organic, ionic-liquid, polymer-gel, hydrogel, ionogel, and solid-state electrolyte systems.

Aqueous electrolytes are among the most widely investigated electrolyte systems because of their high ionic conductivity, low cost, facile processing, and generally favorable safety characteristics. Common aqueous electrolytes include sulfuric acid, potassium hydroxide, sodium sulfate, potassium sulfate, lithium salts, and other inorganic or organic salts. Acidic and alkaline electrolytes can provide rapid ion transport and high electrochemical activity, particularly for certain pseudocapacitive materials. However, their practical voltage window is constrained by water decomposition, with hydrogen and oxygen evolution occurring when the electrode potentials exceed the thermodynamic stability limits of water. Because the energy stored in a capacitor depends on the square of the operating voltage, this limitation can significantly restrict the energy density of aqueous MSCs. Neutral aqueous electrolytes can offer improved compatibility and reduced corrosivity but may exhibit lower ionic conductivity than strongly acidic or alkaline media. Therefore, electrolyte selection must be matched carefully to the electrochemical stability and surface chemistry of the electrode.

Organic electrolytes provide an alternative route toward higher operating voltages. These systems generally consist of an organic solvent containing a dissolved salt, allowing a substantially wider electrochemical stability window than many aqueous electrolytes. The organic electrolytes frequently exhibit lower ionic conductivity and higher viscosity than aqueous systems, which can increase equivalent series resistance and reduce high-rate performance. In addition, the volatility and flammability of some organic solvents raise safety concerns, particularly for wearable devices operating close to the human body. Consequently, the selection of organic electrolytes for wearable MSCs requires consideration of both electrochemical performance and practical safety.

Ionic liquids have attracted considerable attention as next-generation electrolytes because they consist entirely or predominantly of ions and can exhibit negligible vapor pressure, relatively high thermal stability, and wide electrochemical stability windows. Their ability to support high operating voltages makes them attractive for increasing MSC energy density. Furthermore, ionic liquids can potentially improve the safety of flexible devices by reducing the risk of solvent evaporation and leakage. However, high viscosity can limit ion mobility, especially within nanoscale pores and confined MSC architectures. The relatively high cost of some ionic liquids and the sensitivity of their electrochemical behavior to ion size, electrode surface chemistry, water contamination, and temperature also remain important considerations. The development of low-viscosity ionic liquids and carefully engineered ion combinations may therefore be important for future high-performance MSCs. One of the most significant developments in MSC technology is the emergence of gel and solid-state electrolytes. Unlike conventional liquid electrolytes, these systems immobilize the electrolyte within a polymeric, inorganic, or hybrid matrix. Polymer electrolytes can be prepared using materials such as poly(vinyl alcohol), poly(acrylic acid), polyacrylamide, polyethylene oxide, and related polymer networks containing aqueous or nonaqueous ionic species. The resulting electrolyte can simultaneously provide ionic conductivity and structural integrity, making it particularly attractive for flexible and wearable MSCs. Because the electrolyte is physically confined within the polymer matrix, leakage is reduced and the device can be integrated into nonplanar architectures. However, polymer confinement can reduce ionic mobility, particularly when the polymer network is highly crosslinked or when the electrolyte concentration is not optimized.

Hydrogels represent a particularly important class of electrolyte for wearable MSCs because they combine high water content, ionic conductivity, flexibility, and mechanical compliance. A hydrogel electrolyte consists of a three-dimensional polymer network containing a substantial amount of water and dissolved ions. The polymer network prevents macroscopic leakage while allowing ion migration through the hydrated structure. Hydrogels can also be engineered to provide self-healing, stretchability, adhesion, and antifreezing properties. These characteristics are particularly attractive for wearable and skin-conformal energy-storage devices. Nevertheless, evaporation and freezing of water remain significant limitations under changing environmental conditions. Incorporation of hygroscopic components, antifreeze agents, ionic liquids, or highly crosslinked polymer networks can improve environmental stability, although such modifications may simultaneously alter ionic conductivity and interfacial behavior. Ionogels provide another promising strategy in which ionic liquids are immobilized within polymeric or inorganic matrices. They combine the wide electrochemical stability window and low volatility of ionic liquids with the mechanical integrity of solid-like materials. This combination can be highly advantageous for flexible MSCs requiring high operating voltages. However, ionogel performance depends strongly on the interaction between the ionic liquid and the host network. Strong confinement can restrict ion mobility, while insufficient mechanical integrity may lead to deformation-induced failure. Consequently, optimizing the network structure and ionic-liquid loading is essential.

The electrode–electrolyte interface represents the region where electronic and ionic charge carriers interact and where the majority of charge-storage processes occur [33]. Its properties strongly influence the capacitance, rate capability, and stability of MSCs. In EDLC electrodes, electrolyte ions accumulate near the electrode surface without undergoing substantial chemical reactions. The structure of the interfacial charge layer depends on electrolyte concentration, ion size, solvent structure, surface charge density, and electrode pore geometry [73]. At nanoscale dimensions, the classical description of the electric double layer can become insufficient because ion confinement, dielectric effects, solvent reorganization, and ion-ion correlations become increasingly important. This is particularly relevant for highly porous carbon, MXene, and other nanostructured electrodes used in MSCs. Ion size and pore size must therefore be carefully matched. In nanoporous electrodes, ions may need to partially or completely shed their solvation shells before entering narrow pores. This desolvation process can influence both capacitance and rate capability. Counterintuitively, some studies have shown that subnanometer pores can produce enhanced charge storage because confined ions may form highly dense charge layers. However, the exact behavior depends strongly on the electrolyte and electrode chemistry. Therefore, the conventional assumption that larger pores always provide better ion transport and smaller pores always provide greater capacitance is overly simplistic. High-performance MSC electrodes require a coordinated optimization of pore architecture and electrolyte ion characteristics. Surface chemistry is another critical determinant of the electrode–electrolyte interface. Functional groups such as hydroxyl, carboxyl, carbonyl, sulfonic, amine, and other heteroatom-containing species can alter electrode wettability, ion adsorption, local electric fields, and pseudocapacitive behavior [47]. Improved wettability can facilitate electrolyte penetration into porous structures and increase the fraction of electrochemically accessible surface area. However, excessive surface functionalization may reduce electronic conductivity or introduce irreversible Faradaic reactions. Therefore, surface chemistry must be optimized according to the specific electrode–electrolyte combination. In pseudocapacitive systems, the electrode–electrolyte interface becomes even more complex because ions and solvent molecules can participate directly in Faradaic charge-storage processes. Proton insertion, cation adsorption, anion incorporation, and surface redox reactions may contribute to the measured capacitance. The electrolyte can therefore determine which redox states are accessible and how rapidly charge compensation occurs. For transition-metal oxides and conducting polymers, electrolyte composition can influence reaction kinetics, structural stability, and long-term cycling. Consequently, pseudocapacitive performance cannot be evaluated independently of the electrolyte environment. The interfacial resistance between the electrode and electrolyte is another important parameter. Poor electrolyte wetting, blocked pores, weak interfacial contact, or unfavorable surface chemistry can increase ionic transport resistance and reduce active-material utilization [16]. This problem can become particularly severe in thick MSC electrodes, where ions must penetrate through the complete electrode thickness. The resulting concentration gradients can lead to nonuniform charge storage, with the outer regions of the electrode contributing more effectively than the interior. Strategies such as hierarchical porosity, hydrophilic surface modification, nanoscale electrode thickness, and improved electrolyte infiltration can alleviate these limitations. Electrolyte stability also directly influences self-discharge and long-term cycling. Parasitic redox reactions, impurities, dissolved oxygen, electrode corrosion, and ion redistribution can contribute to voltage decay during open-circuit storage. In flexible and wearable devices, water evaporation and electrolyte migration may further accelerate performance degradation. Therefore, encapsulation becomes an important component of the electrolyte strategy. Effective encapsulation must prevent solvent loss and environmental contamination while preserving flexibility and maintaining low interfacial resistance. For wearable MSCs, encapsulation materials should additionally be mechanically compliant and chemically inert toward the electrolyte [66].

The electrode–electrolyte interface is also strongly affected by mechanical deformation. Bending, stretching, or compression can modify electrode porosity, contact area, electrolyte distribution, and ion-transport pathways. In hydrogel and polymer electrolytes, deformation may change polymer-network density and therefore ionic mobility. Similarly, cracking or delamination of an electrode can expose new surfaces while simultaneously disrupting electronic pathways. The design of flexible MSCs should therefore consider the electrode and electrolyte as a coupled mechanical system. Interfacial adhesion between electrode and electrolyte can be especially important because poor adhesion can result in local delamination and loss of electrochemical contact during repeated deformation [11,19]. For next-generation wearable MSCs, multifunctional electrolytes are increasingly attractive. Instead of functioning solely as ion conductors, advanced electrolytes can simultaneously provide flexibility, stretchability, self-healing, adhesion, antifreezing behavior, thermal stability, and even additional electrochemical functionality. Polymer-network engineering and supramolecular interactions can introduce reversible physical crosslinks that enable self-healing after mechanical damage. Double-network and dynamically crosslinked hydrogels can provide improved mechanical strength while maintaining ionic conductivity. Antifreezing formulations can preserve flexibility and ionic transport at subzero temperatures, while hygroscopic components can suppress water evaporation. These developments demonstrate that electrolyte design is moving toward multifunctional interfacial engineering rather than conventional electrolyte selection. An important future direction is the development of electrolyte systems specifically matched to advanced electrode architectures. For example, MXene electrodes require electrolytes capable of efficiently penetrating layered structures without accelerating oxidation, while nanoporous carbon requires appropriate ion sizes and solvation characteristics for efficient pore utilization. Conducting-polymer electrodes may require electrolytes that support reversible ion compensation while minimizing polymer degradation. Three-dimensional electrodes require electrolytes with sufficiently high ionic conductivity and low viscosity to ensure homogeneous penetration throughout the electrode. Consequently, the optimal electrolyte is not universal but must be designed in conjunction with the electrode material and architecture. Overall, electrolyte engineering has become one of the most important strategies for advancing MSC performance beyond the limitations imposed by electrode materials alone. The electrolyte determines the accessible voltage window, ionic transport kinetics, interfacial charge-storage mechanism, environmental stability, and mechanical behavior of the device. Meanwhile, the electrode–electrolyte interface controls how effectively ions interact with electrochemically active sites and how rapidly charge can be transferred and compensated. Future high-performance MSCs will therefore require electrode–electrolyte co-design, in which ion size, solvation structure, pore dimensions, surface chemistry, conductivity, voltage stability, mechanical properties, and environmental resistance are optimized simultaneously. Such integrated interfacial engineering will be particularly critical for wearable MSCs, where high energy and power densities must coexist with flexibility, safety, long-term durability, and reliable operation under real-world mechanical and environmental conditions [38]. At the microscale, electrode–electrolyte behavior can deviate from that observed in conventional bulk supercapacitors because electrode spacing and electrolyte volume become comparable to characteristic transport lengths. Narrow interelectrode gaps in planar MSCs can shorten ion-diffusion pathways but may also increase parasitic leakage and local electric-field interactions between adjacent electrodes. Fringe-field effects can modify the effective field distribution near electrode edges, while confined electrolyte volumes may experience local ion depletion during rapid charging and discharging. These effects can increase interfacial resistance and reduce the effective utilization of electrochemically active regions. Therefore, electrode spacing, finger width, electrolyte thickness, and local electrolyte availability should be considered together when optimizing planar MSC geometry.

## 6. Microfabrication and Manufacturing Strategies

The development of MSCs for next-generation wearable electronics depends not only on the discovery of advanced electrode and electrolyte materials but also on the ability to translate these materials into precisely defined, reproducible, mechanically robust, and scalable device architectures [6,21]. Microfabrication determines the dimensions, geometry, electrode spacing, active-material distribution, interfacial contact, and integration characteristics of an MSC, and consequently has a direct influence on ion transport, electrical resistance, energy density, power density, and mechanical durability. In contrast to conventional supercapacitors, where electrode fabrication generally involves coating or pressing relatively large-area materials onto current collectors, MSC manufacturing requires spatially controlled patterning of electrodes at micro- to millimeter scales. The fabrication strategy must therefore simultaneously satisfy electrochemical, geometrical, mechanical, and manufacturing requirements. This challenge becomes particularly significant for wearable MSCs, where conventional semiconductor fabrication approaches must often be adapted to flexible, stretchable, lightweight, and temperature-sensitive substrates. Accordingly, modern MSC manufacturing increasingly involves a combination of lithographic, printing, laser-based, deposition, assembly, and additive-manufacturing techniques, with the choice of method strongly dependent on electrode chemistry, substrate properties, required resolution, production scale, and intended application. It is important to distinguish process-level scalability from demonstrated manufacturing scalability. A fabrication method may be compatible with high-throughput or large-area processing without having demonstrated reproducible multi-device manufacturing. Practical scalability additionally requires control over manufacturing yield, device-to-device variation, pattern fidelity, thickness uniformity, registration accuracy, batch reproducibility, and performance distribution across large-area substrates [18]. Therefore, printing, laser processing, electrodeposition, and related approaches should be described as potentially scalable unless these manufacturing characteristics have been experimentally demonstrated.

Photolithography remains one of the most established approaches for producing precisely defined MSC architectures because of its high spatial resolution and compatibility with semiconductor manufacturing infrastructure. In a conventional photolithographic process, a photoresist is deposited onto a substrate, selectively exposed to ultraviolet radiation through a patterned mask, and subsequently developed to generate the desired microstructure. Conductive materials or active electrode layers can then be deposited and selectively retained through subsequent processing steps [36]. The ability to control electrode width, interdigitated finger geometry, interelectrode spacing, and multilayer structures with high precision makes photolithography particularly valuable for on-chip MSCs and MEMS-compatible systems. However, conventional photolithography can involve multiple processing steps, specialized equipment, vacuum processing, and photoresist-related chemicals, which can increase manufacturing complexity and cost. More importantly, many flexible polymeric substrates cannot tolerate the temperatures, solvents, or radiation exposure associated with conventional semiconductor processing. Consequently, although photolithography remains highly relevant for high-resolution MSC fabrication, its direct translation to large-area wearable devices is often limited. Soft lithography provides an alternative approach that can reproduce microscale patterns using elastomeric molds. In this strategy, a patterned master is first generated and subsequently used to transfer the desired architecture onto another substrate through molding, stamping, or related processes. Microcontact printing is particularly attractive for MSC fabrication because conductive inks, polymers, nanoparticles, or functional materials can be transferred selectively to defined regions. The method can operate under relatively mild conditions and is therefore compatible with flexible substrates. Soft lithography can also produce complex patterns over comparatively large areas while requiring less sophisticated equipment than conventional photolithography. Nevertheless, the fidelity of the transferred structure depends strongly on mold quality, surface energy, ink rheology, transfer pressure, and substrate characteristics. Repeated use of elastomeric molds may also result in dimensional variation, limiting the reproducibility required for large-scale manufacturing [29].

Screen printing has emerged as an attractive manufacturing technique for flexible and wearable MSCs because of its simplicity, low cost, relatively high throughput, and compatibility with a wide range of substrates. In screen printing, an ink containing conductive or electroactive materials is forced through a patterned mesh onto a substrate. The composition and rheological properties of the ink strongly influence pattern quality and electrode thickness. Carbon materials, conducting polymers, metal nanoparticles, MXenes, and composite formulations can be incorporated into printable inks. Because screen printing can deposit relatively thick material layers in a single step, it is particularly advantageous for increasing areal capacitance. However, the achievable feature size is generally larger than that obtainable by high-resolution lithography, and the roughness and thickness uniformity of printed electrodes can vary with printing conditions. Nevertheless, for wearable MSCs where large-area integration and manufacturing cost may be more important than submicrometer resolution, screen printing offers considerable practical potential. Inkjet printing provides another digitally controlled route for MSC fabrication. Unlike screen printing, inkjet printing deposits material through precisely controlled droplets without requiring a physical stencil. This enables maskless fabrication, rapid prototyping, pattern customization, and efficient use of functional materials. Conductive graphene, carbon nanotubes, MXenes, metal nanoparticles, conducting polymers, and hybrid inks have been explored for printed MSC electrodes. The digital nature of inkjet printing makes it particularly attractive for personalized wearable electronics and rapid optimization of electrode geometries. However, successful printing requires careful control of viscosity, surface tension, particle size, concentration, and solvent evaporation. Poorly optimized inks can produce nozzle clogging, satellite droplets, coffee-ring effects, nonuniform deposition, and weak electrical connectivity. Therefore, the development of stable, high-solid-content, environmentally compatible inks remains an important requirement for scalable inkjet-printed MSCs.

Gravure, flexographic, and aerosol-jet printing provide additional opportunities for high-throughput manufacturing. Gravure and flexographic techniques can achieve relatively high production speeds and are compatible with roll-to-roll processing, making them attractive for large-area flexible electronics. Aerosol-jet printing can provide finer feature sizes than conventional printing approaches and can deposit materials onto nonplanar surfaces, increasing its potential for complex wearable architectures. However, the cost and complexity of specialized printing systems must be considered alongside their resolution and throughput advantages. The most appropriate manufacturing route will ultimately depend on whether the target MSC is intended for laboratory-scale integrated microsystems, flexible consumer electronics, or high-volume wearable applications. Laser-based fabrication has attracted substantial interest because it can simultaneously pattern and modify electrode materials with high spatial selectivity. Laser-induced graphene is a particularly prominent example in which a carbon-rich precursor is locally converted into conductive porous carbon through laser irradiation. This approach enables direct fabrication of interdigitated electrode patterns without requiring conventional masks or multiple lithographic steps. Laser processing can also be used for selective reduction, ablation, drilling, cutting, surface activation, and microstructuring. The local nature of laser energy delivery makes the technique compatible with flexible substrates that may not tolerate extensive thermal processing. Furthermore, the ability to digitally control laser patterns facilitates rapid optimization of electrode geometry. However, the resulting material properties depend strongly on laser wavelength, power, pulse duration, scanning speed, focal position, and precursor composition. Achieving uniform properties over large areas remains an important challenge for industrial implementation.

Electrodeposition is another important strategy for MSC fabrication, particularly for transition-metal oxides, conducting polymers, and metallic current collectors. In electrochemical deposition, the desired material is deposited directly onto a conductive substrate under controlled potential or current conditions [18,45]. This approach offers excellent control over deposition thickness, morphology, and composition while promoting intimate contact between the active material and current collector. Conducting polymers such as polyaniline and polypyrrole can be electrochemically polymerized directly onto patterned electrodes, while metal oxides and hydroxides can be deposited through anodic or cathodic processes. Because deposition occurs selectively on electrically conductive regions, complex architectures can potentially be fabricated without extensive masking. Nevertheless, electrodeposition generally requires conductive substrates and careful control of electrolyte composition, potential, current density, deposition time, and mass loading. Nonuniform deposition can occur in three-dimensional structures if electrical and ionic transport are not properly controlled. Chemical vapor deposition is particularly valuable for producing high-quality graphene, carbon nanotubes, and other advanced nanomaterials directly on substrates. CVD can generate continuous conductive films with excellent electrical properties and relatively low defect densities. It is therefore useful for constructing integrated MSC electrodes where high conductivity and structural uniformity are required. However, conventional CVD processes often involve elevated temperatures that are incompatible with many flexible polymer substrates. Transfer of CVD-grown materials onto flexible substrates can introduce additional interfaces, wrinkles, contamination, and mechanical defects. Consequently, low-temperature CVD and direct growth on flexible substrates remain important research directions. Atomic layer deposition offers an alternative approach for ultrathin conformal coatings with exceptional thickness control. Through sequential self-limiting surface reactions, atomic layer deposition can deposit metal oxides and other functional materials with thicknesses controlled at the atomic scale. This capability is especially valuable for MSC electrodes because nanoscale coatings can maximize active-material utilization while minimizing ion-diffusion distances. ALD can also uniformly coat high-aspect-ratio porous structures, making it attractive for three-dimensional MSC architectures. However, relatively slow deposition rates and high equipment costs limit its suitability for high-throughput manufacturing. Its greatest value may therefore lie in precisely engineered interfaces and ultrathin active layers rather than bulk electrode production. Transfer printing and direct-write techniques have become increasingly important for integrating MSCs onto flexible, stretchable, and unconventional substrates. Transfer processes allow high-performance materials to be fabricated separately under optimized conditions and subsequently transferred onto the target substrate. This approach can overcome the incompatibility between advanced material synthesis and flexible substrate processing. However, transfer interfaces can introduce contact resistance and mechanical weak points. Direct-write approaches, including localized printing, laser writing, and automated deposition, provide greater flexibility by constructing the electrode directly at its final location. The choice between transfer and direct fabrication therefore represents a balance between material quality and integration simplicity.

Three-dimensional manufacturing and additive manufacturing represent particularly promising directions for overcoming the limitations of planar MSC architectures. Conventional planar devices may suffer from limited active-material loading because the available electrode thickness is restricted [24,52]. Additive manufacturing can construct vertically structured electrodes, porous frameworks, micro-pillars, lattices, and multilayer configurations with controlled geometry. Such architectures can increase the amount of active material within a given footprint while maintaining relatively short ion-transport pathways. However, the electrochemical performance of printed 3D electrodes depends strongly on feature resolution, ink composition, drying behavior, structural integrity, and internal porosity. Excessively dense printed structures may impede electrolyte penetration, whereas highly porous structures may have insufficient mechanical strength. Thus, additive manufacturing must be integrated with electrochemical and mechanical design rather than treated purely as a geometrical fabrication technique. The substrate is an equally important consideration in MSC manufacturing. Rigid silicon and glass substrates remain highly suitable for on-chip devices and fundamental studies because of their dimensional stability and compatibility with established microfabrication technologies. However, flexible polymers such as polyimide and polyethylene terephthalate are more attractive for wearable electronics because they can tolerate repeated bending and provide low weight. Elastomeric substrates can additionally support stretchable devices. Paper and textile substrates provide opportunities for low-cost, lightweight, and conformal energy-storage systems. The compatibility between substrate and processing temperature, solvent, pressure, surface energy, and mechanical deformation must be carefully considered. A fabrication technique that produces excellent electrodes on silicon may perform poorly when transferred to a polymer or textile substrate. Manufacturing MSCs at scale also requires careful consideration of device-to-device reproducibility. Laboratory studies often report performance from a small number of devices fabricated under carefully controlled conditions, whereas practical manufacturing requires hundreds, thousands, or millions of devices to exhibit consistent characteristics. Variations in electrode dimensions, active-material loading, electrolyte content, contact resistance, and encapsulation can produce significant performance variation. Consequently, future research should increasingly report statistical distributions rather than only the performance of the best-performing device. Manufacturing yield, process tolerance, defect density, and batch-to-batch reproducibility should become important evaluation parameters alongside electrochemical metrics. For wearable MSCs, encapsulation and packaging must also be integrated into the manufacturing process. Electrolyte evaporation, moisture absorption, mechanical damage, and contamination can substantially alter device performance. Flexible encapsulation layers should provide chemical resistance and environmental protection without excessively increasing device thickness or compromising flexibility. In some applications, biocompatibility may also become important, particularly for skin-contact or implantable systems. Therefore, packaging should be regarded as a functional component of the final device rather than a simple post-fabrication protective layer. Roll-to-roll manufacturing represents a particularly promising pathway toward large-scale production of flexible MSCs [73]. Printing, laser processing, coating, and lamination techniques can potentially be integrated into continuous manufacturing lines, substantially reducing processing time and cost. However, transition from laboratory-scale batch fabrication to continuous manufacturing requires careful control of ink rheology, web tension, drying kinetics, pattern registration, electrode alignment, and electrolyte deposition. The development of environmentally benign inks and low-temperature processing methods will also be important for reducing manufacturing energy consumption and enabling compatibility with sustainable substrates. An important emerging direction is the convergence of microfabrication with digital manufacturing, artificial intelligence, and automated process optimization. The large parameter space associated with printing conditions, laser processing, deposition rates, electrode geometry, and material composition makes conventional trial-and-error optimization inefficient. Machine-learning approaches could potentially correlate fabrication parameters with electrode morphology and device performance, enabling rapid identification of optimized processing windows. Digital manufacturing could also allow MSC geometry to be tailored according to the power and energy requirements of individual wearable systems. Such approaches would facilitate the transition from standardized laboratory devices toward application-specific energy-storage architectures.

Ultimately, no single fabrication technique is universally optimal for MSCs [5,6]. High-resolution lithography is advantageous for on-chip integration, printing techniques are attractive for flexible and scalable manufacturing, laser processing enables rapid maskless patterning, electrodeposition provides intimate electrochemical interfaces, deposition techniques such as CVD and ALD enable high-quality nanoscale materials, and additive manufacturing offers opportunities for complex three-dimensional architectures. The most promising future manufacturing strategies will likely combine several of these approaches within integrated process flows. For example, high-resolution patterning may define the current-collector architecture, printing may deposit the active material, electrodeposition may introduce pseudocapacitive phases, and laser processing may provide final microstructuring. Such hybrid manufacturing strategies can exploit the advantages of individual techniques while minimizing their limitations. In the context of next-generation wearable electronics, the ultimate objective of MSC manufacturing is to achieve a combination of high resolution, high active-material loading, low resistance, mechanical robustness, reproducibility, scalability, low processing cost, and environmental compatibility. The transition from proof-of-concept MSCs to practical wearable energy-storage systems will therefore require a shift from material-centric fabrication toward integrated process engineering. Electrode materials, electrolytes, substrates, current collectors, microstructures, encapsulation, and interconnections must be designed as a unified manufacturing system. Such integration will be essential for producing MSCs that not only demonstrate excellent electrochemical properties under laboratory conditions but also retain those properties across large areas, repeated mechanical deformation, prolonged operation, and realistic manufacturing environments [26,31].

## 7. Microsupercapacitor Device Architectures

The device architecture of a microsupercapacitor (MSC) represents the critical bridge between electrochemically active materials and practical microscale energy-storage systems [6,11]. While electrode chemistry and electrolyte composition establish the intrinsic charge-storage capability of an MSC, the architecture determines how effectively these components can be arranged, electrically connected, ionically accessed, mechanically supported, and integrated with the target electronic platform. This consideration is particularly important because miniaturization fundamentally changes the relative importance of electrode geometry, interelectrode distance, active-material thickness, current-collector configuration, substrate selection, and packaging. Unlike conventional supercapacitors, where stacked electrode configurations dominate, MSCs can exploit planar, vertical, three-dimensional, fiber-shaped, multilayer, and hybrid architectures to satisfy different requirements for power density, energy density, flexibility, stretchability, and integration. Consequently, architecture should be considered a functional electrochemical design parameter rather than simply a geometric arrangement. An optimized MSC architecture must establish short and efficient ionic pathways while simultaneously providing continuous electronic conduction, sufficient active-material loading, low equivalent series resistance, mechanical integrity, and compatibility with scalable microfabrication. The planar in-plane architecture is one of the most widely investigated configurations for MSCs because of its simplicity and compatibility with microfabrication. In a typical planar MSC, the positive and negative electrodes are patterned laterally on a common substrate, most frequently in an interdigitated configuration. The electrode fingers are arranged alternately so that the electrolyte occupies the space between neighboring positive and negative electrodes. Unlike conventional sandwich-type supercapacitors, this arrangement generally does not require a separate porous separator because the electrodes are physically separated within the same plane. The interdigitated structure offers a particularly important advantage at the microscale: the ion-transport distance between the electrodes can be reduced substantially. Since diffusion time scales approximately with the square of characteristic diffusion length, decreasing the interelectrode spacing can significantly improve the rate at which ions redistribute during charging and discharging. This feature contributes to the exceptionally high-power capability frequently associated with planar MSCs.

The electrochemical performance of an interdigitated architecture is strongly dependent on electrode finger width, finger length, number of fingers, thickness, and interelectrode gap [18,66]. Increasing the number and length of electrode fingers can increase the active area within a given footprint, whereas decreasing the finger-to-finger separation can shorten ion-transport pathways. However, these parameters cannot be optimized independently. Very narrow gaps may increase the risk of electrical shorting, leakage currents, and fabrication defects, while excessively large gaps increase ionic resistance. Similarly, increasing electrode thickness can increase active-material loading and areal capacitance but may introduce ion-diffusion limitations. The optimum geometry therefore represents a compromise between active-material utilization, ionic transport, electrical resistance, and manufacturing resolution. Computational modeling and finite-element simulations are increasingly valuable for predicting local current density, ion concentration gradients, and potential distribution within complex interdigitated architectures. The sandwich or stacked architecture represents another important MSC configuration and is conceptually closer to conventional supercapacitors. In this design, the positive and negative electrodes are positioned above and below an electrolyte-containing separator or solid electrolyte. The stacked structure can accommodate relatively high active-material loading and may provide a larger electrode/electrolyte interface within a compact footprint. It can also facilitate the use of electrode materials that are difficult to pattern directly into planar interdigitated geometries. However, the presence of a separator and vertically separated electrodes can increase ionic transport distance and overall device thickness. These characteristics may reduce high-rate performance compared with optimized planar devices, although careful control of electrode thickness and electrolyte properties can mitigate the limitation. Stacked MSCs remain attractive when energy density and compact vertical integration are prioritized over minimum lateral dimensions. Vertical MSC architectures have emerged as an important strategy for overcoming the limitations of conventional planar devices. In a vertical configuration, the electrodes or electroactive structures are arranged along the out-of-plane direction, allowing greater active-material loading within a small surface footprint. Vertically aligned graphene, carbon nanotubes, nanowires, nanosheets, and other one-dimensional or two-dimensional structures can provide direct electron pathways while creating open channels for electrolyte penetration. The resulting architecture can combine high surface area with relatively short ion-diffusion distances. Vertical electrodes are particularly attractive because they provide a route toward increasing areal and volumetric capacitance without simply increasing the lateral device dimensions.

Three-dimensional MSC architectures extend this concept further by introducing hierarchical structures across multiple length scales [3,11]. A three-dimensional electrode can be constructed using porous scaffolds, micro-pillars, nanowire arrays, interconnected carbon networks, foam-like structures, or multilayered architectures. The primary objective is to decouple active-material loading from ion-transport limitations. In an ideal three-dimensional electrode, electrolyte can rapidly penetrate the macroscopic channels, ions can diffuse through mesoporous regions, and charge can accumulate within nanoscale active surfaces. At the same time, a continuous conductive framework maintains efficient electron transport throughout the structure. This hierarchical organization can potentially improve both energy and power densities simultaneously. However, increasing architectural complexity also introduces challenges involving structural uniformity, mechanical stability, fabrication reproducibility, and complete electrolyte infiltration. Therefore, a three-dimensional architecture is advantageous only when the additional active volume remains electrochemically accessible. A particularly promising architecture for wearable electronics is the flexible planar MSC [52,53]. In these devices, the electrode and electrolyte are integrated onto flexible substrates such as polyimide, polyethylene terephthalate, elastomers, paper, or textile materials. Flexible planar architectures preserve the short ion-transport distances of conventional interdigitated MSCs while allowing the complete device to conform to curved surfaces. The mechanical performance is determined not only by the flexibility of the substrate but also by the adhesion and mechanical properties of the electrode, electrolyte, current collector, and encapsulation layers. Brittle active materials may fracture under repeated bending even when deposited on a flexible substrate. Consequently, nanostructuring, porous networks, wrinkled electrodes, elastomeric binders, and mechanically interlocked interfaces are frequently incorporated to accommodate strain. Stretchable MSC architectures require a fundamentally different design philosophy because tensile deformation can be substantially larger than simple bending. Stretchable devices frequently employ serpentine current collectors, wrinkled conductive networks, prestrained elastomers, porous electrodes, and kirigami-inspired structures [72,73]. In these systems, mechanical deformation is accommodated through geometric transformation rather than relying entirely on intrinsic material elasticity. Serpentine structures, for example, can unfold or rotate during stretching while limiting the tensile strain transferred to the electrochemically active layer. Similarly, kirigami and origami architectures introduce strategically positioned cuts or folds that allow substantial deformation while preserving electrical continuity. Such designs are particularly attractive for wearable systems that must follow complex body movements.

Fiber-shaped MSCs represent another important architecture for wearable electronics. Instead of using a conventional planar substrate, the electrochemical components are arranged along one-dimensional fibers or wires. Coaxial, twisted, parallel, and helical configurations have been explored to create energy-storage devices that can be incorporated into textiles and wearable structures. Fiber-shaped MSCs offer excellent flexibility, lightweight characteristics, and compatibility with textile manufacturing. They can also be woven, knitted, or embroidered into fabrics, allowing energy storage to become an integrated component of smart clothing [18]. However, fiber devices face challenges in achieving high active-material loading while maintaining low resistance and uniform electrolyte distribution. The mechanical interface between the fiber core, active material, electrolyte, and encapsulation layer is also critical for long-term operation. Multilayer MSC architectures provide another route to increasing energy density without substantially increasing the lateral footprint. Multiple electrochemical layers can be vertically stacked and electrically connected in series or parallel depending on the desired voltage and capacitance. Series integration increases the total operating voltage and can therefore be used to satisfy the voltage requirements of integrated electronic components. Although the higher voltage can increase the total stored energy of an array, the equivalent capacitance decreases with the number of series-connected cells. Therefore, series connection should not be interpreted as an intrinsic increase in normalized energy density, and the practical benefit should be evaluated by considering the complete array, including its footprint, interconnections, inactive components, and voltage-balancing requirements [45]. Parallel integration, in contrast, increases capacitance and current output while maintaining approximately the same voltage. Multilayer integration therefore provides a powerful method for tailoring MSC output characteristics to the requirements of specific microelectronic systems. However, as the number of layers increases, challenges associated with alignment, interlayer resistance, electrolyte penetration, heat dissipation, and fabrication yield become increasingly important.

Monolithic on-chip MSC architectures are particularly relevant to microelectromechanical systems, integrated circuits, sensors, and autonomous microsystems. In these devices, the MSC is fabricated directly on or within the same substrate as the electronic circuitry. Such integration minimizes the physical distance between the energy-storage component and the load, reducing interconnection resistance and device volume. Silicon-compatible fabrication approaches, thin-film deposition, lithography, electrodeposition, and selective patterning can be used to construct on-chip MSCs. The primary challenge is achieving electrochemical functionality without damaging temperature-sensitive electronic components. Consequently, low-temperature deposition, localized processing, and solid-state electrolytes are particularly valuable for monolithic integration. Hybrid architectures that combine different geometries are increasingly being explored to exploit the advantages of multiple configurations. For example, a planar interdigitated current collector can be combined with a vertically oriented nanostructured electrode, creating a hierarchical electrode in which lateral patterning provides short ion pathways while vertical structures increase active-material loading. Similarly, a three-dimensional porous scaffold can be integrated onto a flexible substrate to produce a high-loading yet mechanically compliant MSC. Hybrid planar-vertical and planar-3D configurations therefore represent an important strategy for overcoming the energy-density limitations of conventional planar devices. Architecture also plays a central role in determining the voltage and capacitance characteristics of integrated MSC arrays. Individual MSCs can be electrically connected in series, parallel, or more complex network configurations. Through appropriate series-parallel arrangement, MSC arrays can be engineered to deliver the voltage and current requirements of specific electronic components. This approach is particularly important for wearable electronics, where the energy-storage system may need to operate sensors, wireless communication modules, displays, or physiological monitoring components. The architecture of the electrolyte is also increasingly important [19]. Conventional liquid electrolytes can be incorporated into planar or stacked structures, but gel, hydrogel, ionogel, and solid-state electrolytes enable greater freedom in designing flexible and three-dimensional devices. In some advanced architectures, the electrolyte itself functions as a structural layer, adhesive interface, or mechanically reinforcing component. Self-healing and stretchable polymer electrolytes can further improve device reliability by maintaining ionic contact after mechanical deformation. Thus, modern MSC architecture should be considered a coupled electrode–electrolyte -substrate system, rather than an electrode geometry surrounded by a separate electrolyte. An important consideration when comparing MSC architectures is the distinction between footprint efficiency and volumetric efficiency. Planar devices can provide excellent integration density and high-power capability but may suffer from limited active-material volume. Three-dimensional and multilayer architectures can substantially improve volumetric and areal energy density but often introduce greater fabrication complexity. Fiber-shaped devices offer exceptional mechanical flexibility and textile compatibility but may have lower volumetric utilization depending on their construction. Consequently, the selection of architecture should be guided by the requirements of the intended application rather than by a universal ranking of device configurations [92]. For next-generation wearable electronics, the most promising MSC architectures are likely to be those that combine short ionic transport pathways, high active-material loading, mechanically adaptive structures, multifunctional electrolytes, and direct integration with electronic systems. Planar interdigitated architectures remain highly attractive for microscale integration and high-power operation, whereas vertical and three-dimensional structures offer important opportunities for increasing energy density. Flexible, stretchable, fiber-shaped, and textile-integrated architectures provide additional pathways toward conformal and body-compatible energy storage. The future development of MSCs will therefore likely involve increasingly hierarchical architectures in which nanoscale active materials, microscale electrode patterns, and macroscale mechanical structures are deliberately integrated. Ultimately, device architecture determines how effectively the intrinsic properties of electrode and electrolyte materials are converted into practical energy-storage performance. The ideal MSC architecture should minimize inactive volume, maximize electrochemically accessible area, shorten ion-transport distances, maintain low electronic resistance, support sufficient active-material loading, and preserve mechanical integrity during operation. At the same time, it must be compatible with reproducible and scalable manufacturing. The continued evolution from simple planar interdigitated devices toward three-dimensional, multilayer, stretchable, fiber-shaped, and monolithically integrated architectures represents a fundamental shift from material-centric MSC development toward system-level energy-storage engineering. This architectural evolution will be essential for realizing MSCs capable of meeting the simultaneous energy, power, flexibility, durability, and integration requirements of next-generation wearable and microscale electronic systems. The selection of MSC architecture involves a balance between ion-transport distance, active-material loading, device thickness, volumetric performance, and fabrication complexity. Planar interdigitated architectures provide short lateral ion-transport pathways and are attractive for in-plane integration, but their accessible electrode volume and areal loading can be limited by the interelectrode geometry. Sandwich architectures can accommodate greater active-material loading and therefore provide higher areal or volumetric storage capacity, but increasing electrode thickness generally increases ion-transport distances and may introduce concentration polarization at high rates. Vertical and three-dimensional architectures can increase the active surface area and improve volumetric utilization, although their fabrication and structural control are generally more demanding. Thus, architecture selection should be based on the requirements of the target application rather than on electrochemical performance alone.

## 8. Flexible, Stretchable and Deformable MSCs

The rapid development of wearable, epidermal, textile, implantable, and deformable electronics has created a fundamental requirement for energy-storage systems that can operate reliably under repeated mechanical deformation [72,73]. Conventional rigid energy-storage devices are poorly suited to such applications because their electrochemical components, current collectors, separators, and packaging systems are generally designed for mechanically static environments. Microsupercapacitors (MSCs), owing to their small dimensions, short ion-transport pathways, and compatibility with microfabrication, have emerged as promising power sources for miniaturized wearable electronics [16,51]. However, achieving high electrochemical performance while simultaneously maintaining flexibility, stretchability, bendability, and structural integrity represents a substantially more complex materials and device-engineering problem than simply miniaturizing a conventional supercapacitor. Flexible MSCs must preserve electrical connectivity and electrochemical activity during bending, stretchable MSCs must accommodate substantial tensile strain without fracture or delamination, and deformable MSCs must tolerate complex combinations of bending, twisting, compression, folding, and stretching. Consequently, the development of mechanically adaptive MSCs requires coordinated engineering of electrode materials, electrolytes, substrates, current collectors, interfaces, device architectures, and encapsulation [72,73]. Flexibility in an MSC generally refers to the ability of the device to undergo bending or folding without significant loss of electrochemical performance. This characteristic is particularly important for wearable electronics because devices integrated onto the skin, clothing, or curved surfaces rarely experience a perfectly planar geometry. A flexible MSC must therefore maintain continuous electron-transport pathways and stable electrode–electrolyte interfaces when subjected to repeated curvature. The mechanical response depends strongly on the thickness and modulus of each component. Thin-film electrodes generally experience lower absolute strain during bending than thick electrodes, while porous and interconnected structures can accommodate deformation more effectively than dense brittle films. Flexible substrates such as polyimide, polyethylene terephthalate, polyethylene naphthalate, paper, and elastomeric polymers have therefore been widely explored as platforms for MSC integration. A key principle governing flexible MSC design is the distinction between material flexibility and structural flexibility. A material may possess a high intrinsic Young’s modulus but still participate in a flexible device if it is configured into an appropriate architecture. Conversely, a nominally flexible material can fail when deposited as a dense, poorly adhered layer on a rigid interface. For example, brittle transition-metal oxides can be integrated into flexible MSCs when deposited as nanosheets, nanowires, nanoparticles, or ultrathin coatings on compliant conductive frameworks [81]. Similarly, graphene, carbon nanotubes, MXenes, and conducting polymers can form interconnected networks that maintain electrical pathways during bending. Thus, flexibility is an emergent property resulting from the interaction among material properties, geometry, thickness, adhesion, and mechanical boundary conditions.

Carbon-based materials have played a particularly important role in flexible MSCs because of their combination of conductivity, low density, chemical stability, and mechanical resilience. Graphene can form highly conductive thin films, while carbon nanotubes can create entangled networks capable of sustaining repeated deformation. Laser-induced graphene is also attractive because porous graphene-like structures can be patterned directly onto flexible substrates. These carbon frameworks can function simultaneously as current collectors and electrochemically active electrodes, reducing the number of interfaces that may otherwise become mechanically vulnerable. However, carbon materials alone often provide moderate capacitance, motivating their integration with pseudocapacitive components such as transition-metal oxides and conducting polymers. MXenes have emerged as another important material family for flexible MSCs because of their high electrical conductivity, hydrophilic surfaces, and layered morphology. Their ability to form thin, solution-processable films facilitates fabrication of flexible and conformal electrodes. However, mechanical stability during repeated bending is strongly influenced by interflake interactions, film thickness, substrate adhesion, and electrolyte compatibility. Excessively dense MXene films may develop cracks or delamination during repeated deformation, whereas porous or hybrid MXene networks can distribute mechanical stress more effectively. Hybridization with carbon nanotubes, graphene, nanofibers, or conducting polymers can further improve mechanical integrity by creating interpenetrating conductive networks. Conducting polymers are particularly attractive for mechanically deformable MSCs because of their intrinsic flexibility and ability to undergo moderate deformation without catastrophic fracture. Polyaniline, polypyrrole, and PEDOT-based materials can be deposited as thin coatings or integrated into porous conductive frameworks. Their major limitation is electrochemical volume change during repeated redox cycling, which can generate internal mechanical stress and gradually degrade the electrode. Nanostructuring the polymer and integrating it with carbon or other mechanically stable scaffolds can reduce this problem by providing space for expansion and contraction while maintaining electrical connectivity. Such hybrid architectures can simultaneously improve capacitance, rate capability, and mechanical durability.

The electrolyte is equally important in determining the mechanical performance of flexible and deformable MSCs [33]. Conventional liquid electrolytes can provide high ionic conductivity but may leak, evaporate, or redistribute when the device is bent or inverted. Solid-state polymer electrolytes, gel electrolytes, hydrogels, and ionogels can address these limitations by immobilizing the ionic phase within a mechanically stable matrix. In wearable MSCs, the electrolyte can therefore serve not only as an ion conductor but also as a flexible structural component. Polymeric gel electrolytes can maintain intimate contact between electrodes under bending, while hydrogels can provide high ionic conductivity and mechanical compliance [72]. The challenge is to achieve sufficient ionic mobility without sacrificing mechanical strength. Increasing polymer crosslink density, for example, may improve mechanical robustness but can restrict ion transport. Stretchable MSCs present a more demanding mechanical challenge because stretching introduces tensile strains that can exceed the fracture limits of conventional electrode materials. Intrinsically stretchable electrodes can be produced from elastomeric conductive composites, conductive polymer networks, carbon nanotube films, graphene networks, MXene-elastomer composites, and other deformable materials. However, intrinsic stretchability is often insufficient to achieve the large strains required by wearable applications. Structural engineering is therefore frequently used to accommodate deformation. Wrinkled films, serpentine interconnects, porous networks, buckled structures, and kirigami or origami-inspired architectures can enable significant macroscopic deformation while limiting the local strain experienced by the electroactive material [52]. Wrinkled and buckled structures are particularly effective because they introduce excess surface area into the electrode before mechanical stretching occurs. When the substrate is stretched, the wrinkles gradually unfold rather than directly stretching the active material. This mechanism can substantially increase the apparent strain tolerance of otherwise brittle or moderately flexible electrodes. Similarly, serpentine conductive pathways can undergo geometric rotation and elongation during stretching, reducing localized stress concentration. Such approaches are especially useful for integrating MSCs with elastomeric substrates intended to follow the deformation of human skin. Kirigami-inspired MSC architectures provide another highly effective strategy for large deformation. By introducing carefully designed cuts into an electrode or substrate, a two-dimensional structure can transform into a three-dimensional configuration under tensile strain. This geometric transformation enables substantial stretching while reducing the intrinsic strain imposed on the electrochemical components. Origami-inspired folding provides a related approach in which controlled folding creates compact but mechanically adaptable structures. These architectures also offer the possibility of increasing the effective electrode area within a small initial footprint, potentially improving the energy-storage capacity of the system. Nevertheless, repeated folding and unfolding can generate localized stress concentrations, requiring careful optimization of cut geometry, material thickness, and interfacial adhesion. Fiber-shaped MSCs are particularly relevant to wearable and textile electronics because their one-dimensional geometry naturally supports bending and twisting. Electrodes can be constructed around flexible fibers using carbon nanotubes, graphene, metal nanowires, conducting polymers, MXenes, or carbon fibers. Two electrodes can be arranged in parallel, twisted together, or separated through a coaxial configuration containing a solid or gel electrolyte [52]. Fiber MSCs can subsequently be woven, knitted, embroidered, or integrated into fabrics, creating distributed energy-storage systems that do not require a rigid planar form factor. However, achieving high capacitance per unit length or volume while maintaining mechanical durability remains challenging. Uniform deposition of electroactive materials around cylindrical substrates is also more difficult than coating planar surfaces.

Textile-integrated MSCs represent an important extension of fiber-shaped devices. Conductive fibers, yarns, fabrics, and textile substrates can function as current collectors or structural supports for active materials. Such systems are particularly attractive for smart clothing because the energy-storage component can become part of the textile itself rather than being attached as a separate rigid module. Nevertheless, textile integration introduces additional mechanical complexity because fabrics undergo repeated bending, compression, stretching, abrasion, washing, and deformation in multiple directions. The adhesion of active materials to textile fibers must therefore be sufficiently strong to prevent material loss during use. Encapsulation strategies must additionally protect the electrolyte while maintaining textile flexibility and breathability where required. Mechanical durability should be evaluated systematically rather than demonstrated through a single bending photograph. A reliable flexible MSC should maintain its electrochemical performance after hundreds, thousands, or more mechanical cycles under controlled deformation. Bending radius, tensile strain, twisting angle, compression, deformation frequency, and number of cycles should be reported alongside electrochemical measurements. Importantly, mechanical testing should ideally be performed simultaneously with electrochemical cycling because mechanical deformation and electrochemical reactions can interact. For example, repeated ion insertion and extraction may weaken an electrode before mechanical deformation is applied, while mechanical strain can alter electrolyte accessibility and accelerate electrochemical degradation. Combined electrochemical-mechanical testing therefore provides a more realistic assessment of device reliability [72,73].

The electrode-substrate and electrode–electrolyte interfaces are among the most vulnerable regions during deformation [93]. Differences in elastic modulus and thermal expansion coefficient between adjacent layers can generate interfacial stress. Poor adhesion may cause delamination even when the individual materials remain intact. Interfacial engineering can address this problem through surface functionalization, chemical bonding, mechanical interlocking, polymer binders, gradient structures, or in situ growth of active materials directly on conductive substrates. A mechanically graded interface, in which the modulus gradually changes between rigid and flexible components, can reduce stress concentration and improve cycling stability. Encapsulation is particularly important for flexible and stretchable MSCs because mechanical deformation can expose the device to environmental contaminants and accelerate electrolyte loss. Conventional rigid encapsulation layers can compromise flexibility, whereas ultrathin polymeric barrier films and elastomeric encapsulants can preserve deformability while providing environmental protection. For wearable devices, encapsulation must also consider moisture transport, skin compatibility, thermal stability, and long-term adhesion. In certain applications, breathable or selectively permeable encapsulation may be desirable, whereas implanted systems require more stringent barriers against body fluids. An additional challenge is maintaining electrochemical performance while reducing device thickness. Thin MSCs are naturally attractive for wearable electronics because they minimize bulk and improve conformability. However, reducing electrode thickness decreases active-material loading and can reduce total stored energy. The solution is not simply to increase material loading but to develop architectures with high volumetric and areal utilization. Vertically aligned nanostructures, porous three-dimensional electrodes, multilayer structures, and high-conductivity current collectors can increase active-material utilization without creating excessively long ion-diffusion pathways. Therefore, future flexible MSC research should emphasize areal and volumetric energy density rather than relying exclusively on gravimetric metrics. Another important issue is mechanical deformation of the electrolyte itself. In hydrogel and polymer electrolytes, stretching can change polymer-chain spacing and ionic mobility. Water loss can further alter the mechanical and electrochemical properties over time. Antifreezing and anti-drying electrolyte formulations are therefore increasingly important for wearable MSCs exposed to variable environmental conditions. Incorporating hygroscopic molecules, ionic liquids, or strongly bound water can improve environmental stability, although these modifications may alter viscosity, conductivity, and electrochemical stability. Consequently, electrolyte optimization must consider mechanical and environmental requirements simultaneously. The integration of flexible MSCs with wearable electronics also requires consideration of electrical interconnections. Even when the MSC itself remains mechanically stable, rigid interconnects can become failure points during bending or stretching. Conductive elastomeric interconnects, serpentine metal traces, stretchable nanocomposite conductors, and textile-based connections can reduce mechanical mismatch between the MSC and the electronic system. Furthermore, multiple MSC units can be connected in series and parallel to provide the voltage and current required by wearable sensors, communication modules, and other low-power electronics. Distributed MSC arrays can also improve mechanical conformability because deformation is shared across multiple smaller units rather than concentrated in one large device. The long-term objective is therefore not simply to produce an MSC that can be bent without immediate failure but to develop mechanically adaptive energy-storage systems capable of maintaining stable electrochemical performance throughout realistic operational conditions. Such systems should tolerate repeated deformation, temperature fluctuations, humidity, mechanical abrasion, and prolonged electrochemical cycling. This requires a transition from isolated demonstrations of flexibility toward standardized mechanical reliability protocols. Reporting capacitance retention after repeated bending or stretching is useful, but additional parameters such as leakage current, equivalent series resistance, voltage retention, and energy/power density under deformation should also be evaluated. The flexible, stretchable, and deformable MSCs represent a critical technological direction for wearable energy storage [82,93]. Progress in this field has been enabled by conductive carbon networks, MXenes, conducting polymers, transition-metal compounds, solid-state electrolytes, hydrogels, elastomeric substrates, and mechanically adaptive architectures. Nevertheless, the central challenge remains the simultaneous optimization of electrochemical performance, mechanical compliance, interfacial adhesion, environmental stability, and scalable manufacturing. Future advances are likely to emerge from hierarchical material architectures in which nanoscale electrochemical functionality is combined with microscale patterning and macroscale mechanical design. The convergence of stretchable materials, self-healing electrolytes, textile integration, three-dimensional architectures, and scalable printing or laser fabrication could ultimately enable MSCs that function as integral components of electronic skins, smart textiles, flexible sensors, wearable health-monitoring systems, and other emerging electronic platforms. Such mechanically robust energy-storage technologies will be essential for moving MSCs from laboratory demonstrations toward reliable, continuously deformable power sources for next-generation wearable electronics. Mechanical deformation can directly influence the electrochemical behavior of flexible and stretchable MSCs. Repeated bending or stretching may generate cracks within brittle active layers, weaken electrode–current-collector adhesion, and promote interfacial delamination. In gel or polymer electrolytes, deformation can also redistribute the electrolyte and alter local ion-accessible regions. These structural changes may increase interfacial and charge-transfer resistance and cause deformation-dependent changes in impedance. Therefore, mechanical reliability should not be evaluated solely from capacitance retention after deformation; monitoring impedance and other electrochemical parameters during repeated deformation can provide additional information about progressive interfacial degradation. In gel and polymer electrolytes, repeated deformation may additionally induce local electrolyte redistribution or microphase separation, which can modify ion transport and interfacial contact and consequently produce deformation-dependent changes in interfacial impedance.

**Figure 6:**
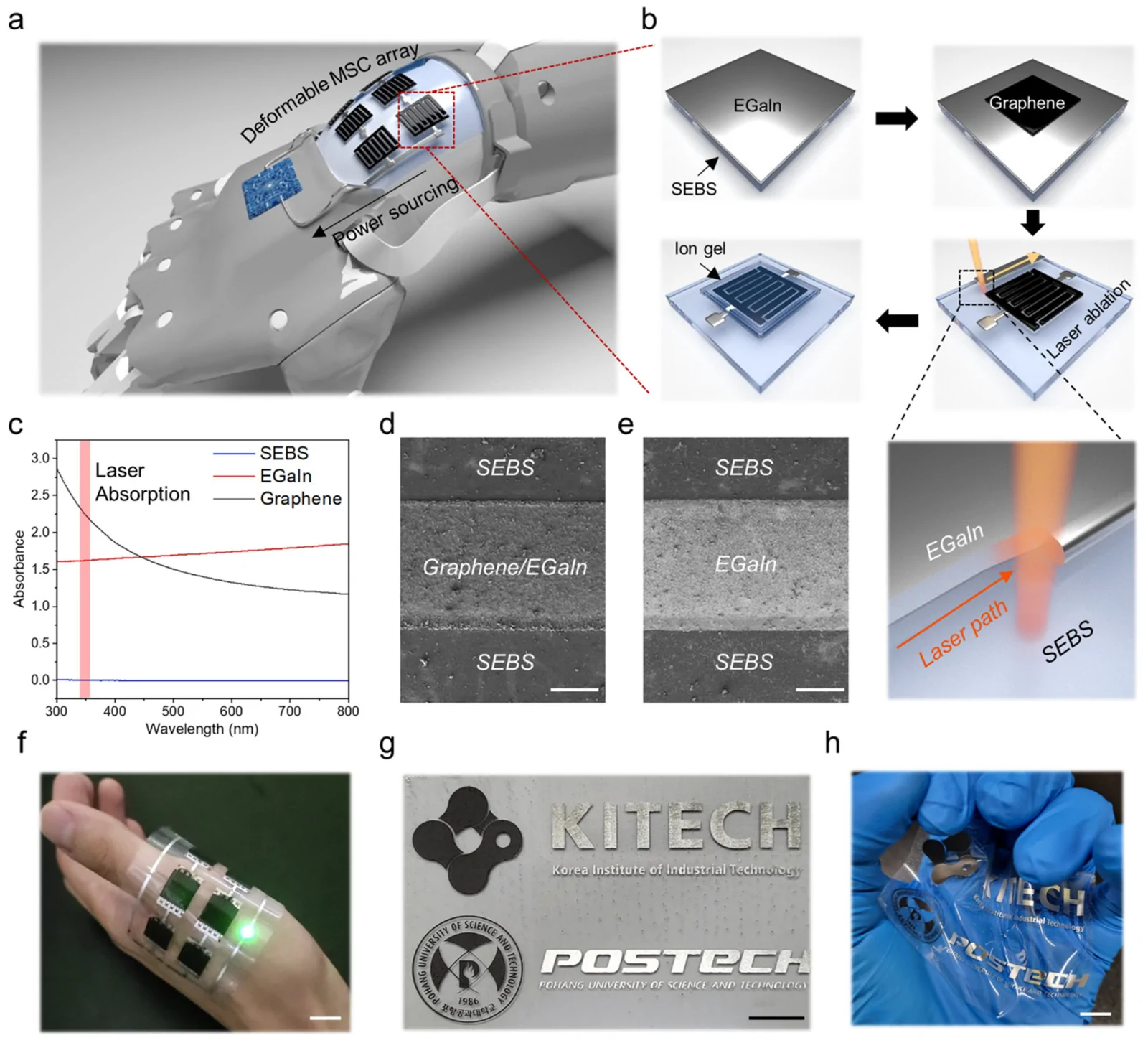
Design, fabrication, and demonstration of a deformable EGaIn-based micro-supercapacitor (MSC): (**a**) schematic illustration of an integrated soft-electronic system incorporating a deformable energy-storage component; (**b**) fabrication process of the EGaIn-based MSC; (**c**) UV-vis spectra of SEBS, EGaIn, and graphene; FE-SEM images of laser-ablated (**d**) graphene/EGaIn and (**e**) EGaIn electrodes (scale bar: 200 μm); and photographs of (**f**) institutional logos, (**g**) deformed logos, and (**h**) an LED connected to the MSC circuit (scale bar: 1 cm). Reproduced with permission from Ref. [71]. Copyright (2024) Nature Publishing Journal.

Kim et al. developed a highly deformable microsupercapacitor (MSC) by integrating graphene with a eutectic gallium–indium (EGaIn) liquid-metal current collector on a stretchable polystyrene-block-poly(ethylene-co-butylene)-block-polystyrene (SEBS) substrate [71]. The key innovation of this work is the replacement of conventional rigid metallic current collectors with EGaIn, whose liquid-state characteristics enable the current-collecting network to accommodate substantial mechanical deformation without catastrophic cracking or electrical failure. Graphene was employed as the electroactive material, while ultraviolet laser ablation was used to directly pattern the graphene/EGaIn layer into well-defined interdigitated MSC electrodes. Because graphene and EGaIn strongly absorb the 355 nm laser wavelength whereas the SEBS substrate exhibits minimal absorption, selective laser ablation could be achieved without substantially compromising the deformability of the polymer substrate. The authors further demonstrated that the electrochemical performance could be optimized through control of the interelectrode gap and graphene mass loading. The optimized device achieved an areal capacitance of 1336 μF cm^−2^ at 2 μA cm^−2^ and maintained reliable rate performance. Importantly, the MSC retained approximately 90% of its initial capacitance after repeated folding and 20% tensile deformation over 1000 cycles, while more than 90% of the initial electrochemical performance was maintained after 10,000 charge–discharge cycles. The device also remained operational under complex mechanical deformations, including stretching, folding, twisting, wrinkling, and clumping. To demonstrate practical functionality, the authors integrated MSC arrays with light-emitting diodes, showing that the deformable energy-storage unit could continuously supply power while undergoing mechanical deformation. This study provides an important design strategy for wearable and soft electronics because it simultaneously addresses the mechanical limitations of conventional planar MSCs and the fabrication challenges associated with patterning liquid-metal conductors (Figure 6a–h). More broadly, the work demonstrates that combining liquid-metal current collectors, flexible polymer substrates, carbon-based electrodes, and direct laser patterning can provide a versatile platform for highly deformable microscale energy-storage devices. The demonstrated compatibility with complex mechanical deformation makes this architecture particularly attractive for electronic skin, soft robotics, smart textiles, and other wearable systems requiring energy-storage components capable of conforming to dynamic surfaces.

**Figure 7:**
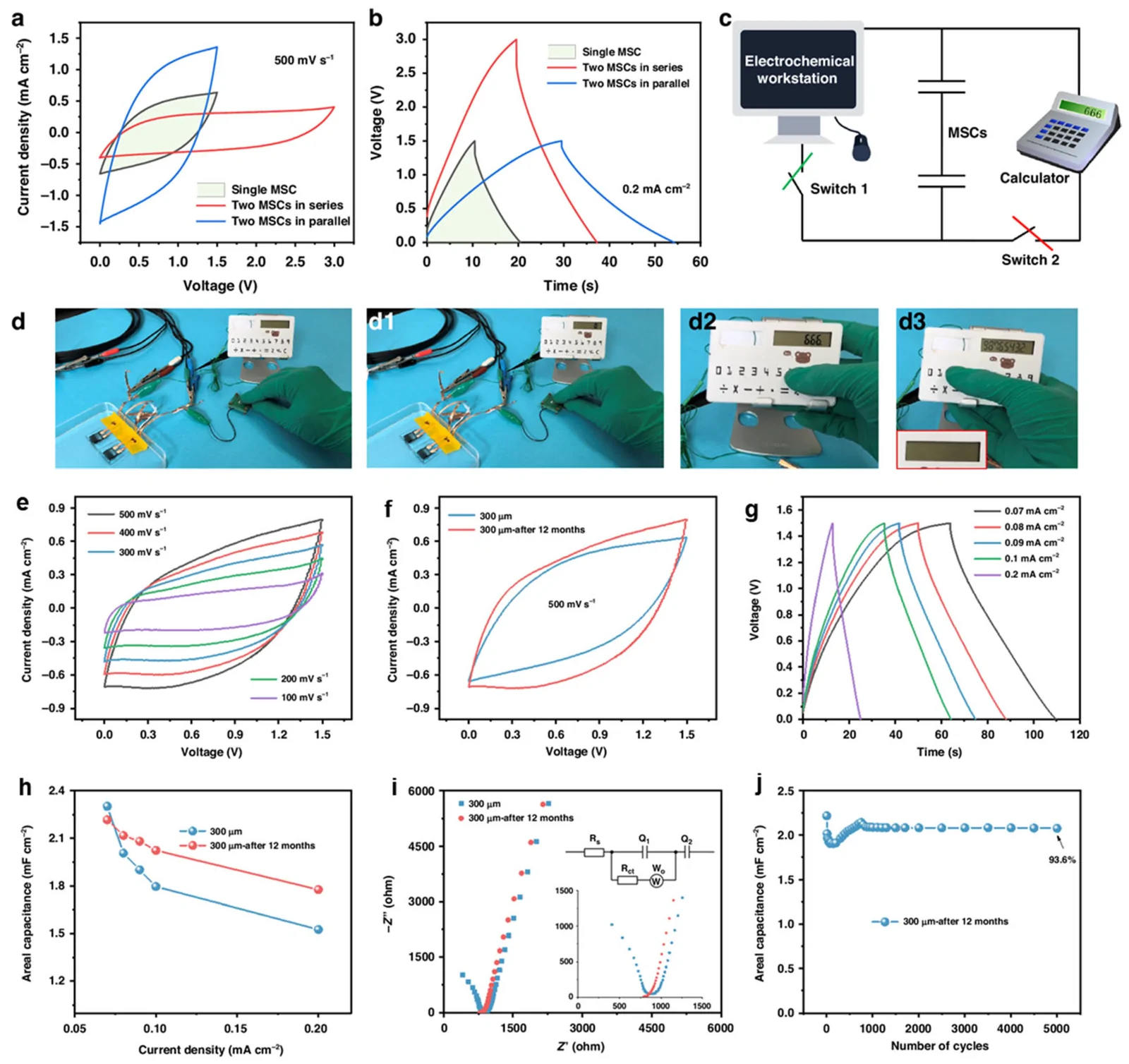
(**a**) CV curves recorded at 500 mV s^−1^ and (**b**) GCD profiles measured at 0.2 mA cm^−2^ for the MSC-300 device; (**c**) schematic representation of the equivalent circuit; (**d**) photographs of the application-testing system; (**e**) CV curves at different scan rates; (**f**) CV curves recorded at 500 mV s^−1^; (**g**) GCD profiles at different current densities; (**h**) areal capacitance; (**i**) electrochemical impedance spectra; and (**j**) cycling stability. Reproduced with permission from Ref. [72]. Copyright (2024) Nature Publishing Journal.

Han et al. developed flexible, stretchable, and attachable symmetric microsupercapacitors (MSCs) by integrating laser-direct-written graphene (LG) electrodes with a phosphoric acid–nonionic surfactant liquid-crystal (PA–NI LC) gel electrolyte [72]. The work addresses an important limitation of conventional flexible MSCs, namely the difficulty of simultaneously achieving mechanical deformability and a sufficiently wide electrochemical operating voltage (Figure 7a–j). The laser-direct-writing strategy enables the formation of interdigital graphene electrodes with a controllable architecture, while the PA–NI LC gel electrolyte provides a mechanically compliant and electrochemically stable medium for ion transport. An important feature of the device is the ability of the liquid-crystal-based electrolyte to support a relatively wide voltage window, which is particularly advantageous because the energy density of a supercapacitor scales with the square of its operating voltage. The authors systematically investigated the electrochemical behavior and mechanical response of the MSCs under different deformation conditions, demonstrating their ability to maintain stable energy-storage characteristics during bending and stretching. The devices also exhibited favorable long-term cycling durability, indicating that the combination of the graphene electrode and gel electrolyte can accommodate repeated mechanical deformation without severe electrochemical degradation. Importantly, the MSCs could be assembled into configurations suitable for attachment to nonplanar surfaces, demonstrating their potential for wearable and conformal electronics. The study therefore highlights the importance of integrating electrode fabrication and electrolyte engineering rather than optimizing these components independently. In particular, laser-direct-written graphene provides a lightweight and conductive carbon framework with a readily programmable microscale geometry, while the PA–NI LC gel electrolyte simultaneously contributes ionic conductivity, flexibility, and an expanded electrochemical stability window. This combination provides a promising strategy for overcoming the conventional trade-off between mechanical compliance and electrochemical performance in planar MSCs. From the perspective of next-generation wearable electronics, the work is significant because practical energy-storage units must remain functional under continuous bending, stretching, and attachment to irregular surfaces while delivering sufficient voltage and stable cycling performance. Overall, this study demonstrates that the rational integration of laser-patterned graphene with a functional liquid-crystal gel electrolyte can produce MSCs with a useful combination of flexibility, stretchability, wide-voltage operation, and durability, providing an important pathway toward conformable energy-storage components for wearable and soft electronic systems.

## 9. Strategies for Achieving High Performance

Achieving high performance in MSCs requires a fundamentally integrated approach in which electrode chemistry, nanoscale architecture, electrolyte properties, device geometry, microfabrication, and mechanical design are optimized simultaneously [26,53]. Although considerable progress has been achieved through the development of high-capacitance electrode materials, improvements in a single parameter rarely translate directly into superior full-device performance. For example, an electrode exhibiting exceptionally high gravimetric capacitance may provide limited practical energy density when its active-material loading is low, while a highly conductive electrode may exhibit inadequate energy storage [55]. Similarly, increasing electrode thickness can improve areal capacitance but may introduce severe ionic diffusion limitations. Consequently, the central objective of high-performance MSC development is to overcome the conventional trade-offs among capacitance, operating voltage, rate capability, mass loading, mechanical durability, and manufacturing compatibility. This requires a multiscale design strategy extending from atomic-level surface chemistry to device-level architecture. One of the most effective approaches for improving MSC performance is the rational engineering of electrode porosity. Because charge storage occurs predominantly at or near the electrode–electrolyte interface, maximizing the electrochemically accessible surface area is essential. However, simply increasing the specific surface area does not guarantee improved device performance. Extremely narrow micropores may be inaccessible to solvated ions, whereas excessively large pores can reduce the surface area available for charge accumulation. Hierarchical porous structures containing interconnected micro-, meso-, and macropores can therefore provide a more favorable balance between charge-storage area and ion transport. Micropores can contribute substantially to electric-double-layer formation, mesopores can facilitate ion diffusion, and macropores can serve as electrolyte reservoirs and transport channels. Such multiscale architectures are particularly valuable for high-rate MSC operation. Increasing the electrical conductivity of the electrode is another critical strategy. High internal resistance limits power delivery, produces voltage losses, and reduces energy efficiency during rapid charge-discharge operation. Conductive carbon frameworks, graphene, carbon nanotubes, MXenes, metallic nanostructures, and conductive polymers can be used to establish continuous electron-transport pathways. In composite electrodes, the conductive phase should ideally form a percolating network throughout the electroactive material. However, excessive incorporation of conductive additives reduces the fraction of electrochemically active material and may decrease overall volumetric performance. Therefore, the objective is to establish an efficient conductive network using the minimum inactive fraction necessary to achieve low resistance.

The incorporation of pseudocapacitive materials provides an effective route for increasing capacitance beyond the limits of conventional carbon-based EDLC electrodes. Transition-metal oxides, hydroxides, sulfides, nitrides, phosphides, and conducting polymers can undergo rapid reversible redox reactions and thereby increase charge-storage capacity [33,56]. Nevertheless, pseudocapacitive materials often suffer from lower conductivity or structural instability during repeated cycling. Their performance can therefore be improved by depositing them as ultrathin coatings, nanoparticles, nanowires, nanosheets, or conformal layers on conductive scaffolds. This architecture reduces ion-diffusion distances and increases the fraction of active material that can participate in rapid electrochemical reactions. The optimal loading must be carefully controlled because excessive deposition can block pores and increase transport resistance. Hybrid and heterostructured electrodes provide another powerful strategy for simultaneously improving multiple performance parameters. Combining an EDLC material such as graphene, carbon nanotubes, or porous carbon with a pseudocapacitive component can create complementary charge-storage mechanisms. The carbon phase provides high conductivity and structural stability, while the redox-active phase contributes additional capacitance. MXene-carbon, MXene-conducting polymer, graphene-metal oxide, and carbon-transition-metal compound systems are representative examples. More advanced heterostructures can introduce electronically coupled interfaces that modify charge-transfer kinetics and surface electronic structures [44,52]. However, improvements should be evaluated at the complete-device level because apparent synergistic effects can sometimes originate simply from increased active-material loading or differences in electrode mass. Electrolyte engineering provides another direct route to improving MSC performance. Because energy density scales with the square of the operating voltage, expanding the electrochemical stability window can provide a disproportionately large increase in stored energy. Aqueous electrolytes generally provide high ionic conductivity but relatively limited voltage windows, whereas organic electrolytes and ionic liquids can support higher operating voltages. Gel and solid-state electrolytes can additionally improve mechanical stability and enable flexible or stretchable configurations. The ideal electrolyte must balance ionic conductivity, electrochemical stability, viscosity, safety, environmental stability, and compatibility with the electrode. More importantly, electrolyte and electrode properties should be designed together because ion size, solvation behavior, and surface chemistry strongly influence the accessible pore structure and interfacial capacitance.

Device architecture is equally important for achieving high performance [44,56]. Interdigitated planar MSCs reduce the lateral distance between positive and negative electrodes and can therefore provide excellent power capability. Increasing the number and length of electrode fingers can increase the active area within a limited footprint, whereas optimized finger spacing can reduce ionic resistance. Vertical and three-dimensional architectures offer complementary advantages by increasing active-material loading while maintaining short ion-transport pathways. Multilayer configurations can further increase areal and volumetric energy density. The most effective architecture therefore depends on the intended application and should be optimized according to footprint, thickness, energy requirement, and mechanical constraints rather than according to capacitance alone. High active-material loading is particularly important for translating laboratory-scale MSC research into practical devices. Many reported MSCs achieve impressive gravimetric capacitance using ultrathin electrodes with very small quantities of active material. Such values can become misleading when translated to practical energy storage because the total amount of stored energy remains small. Increasing areal and volumetric capacitance through thicker electrodes, three-dimensional scaffolds, multilayer structures, and high-density porous architectures is therefore essential. However, increasing loading must be accompanied by efficient electrolyte penetration and electron transport. The objective should be high electrochemically utilized loading, rather than simply high mass loading. Reducing equivalent series resistance is another central strategy for maximizing power performance. Resistance originates from multiple components, including the intrinsic resistance of the electrode, current collector resistance, contact resistance, electrolyte resistance, and interfacial resistance. Optimizing current-collector geometry, improving electrode adhesion, increasing electronic conductivity, reducing interelectrode distance, and selecting electrolytes with high ionic conductivity can collectively minimize these losses. In microscale devices, even small contact resistances can become significant relative to the total device resistance, making interface engineering particularly important. Mechanical integration is essential when MSCs are intended for wearable electronics. Flexible and stretchable architectures should maintain electrochemical performance under bending, twisting, folding, and stretching. Wrinkled electrodes, serpentine interconnects, porous conductive networks, fiber-shaped configurations, and kirigami-inspired structures can accommodate deformation without imposing excessive strain on the electroactive materials. Solid-state, gel, hydrogel, and ionogel electrolytes can reduce leakage and maintain electrode–electrolyte contact. Importantly, mechanical durability should be evaluated simultaneously with electrochemical cycling because repeated deformation can modify electrode morphology, electrolyte distribution, and interfacial contact. Finally, achieving genuinely high-performance MSCs requires standardized and realistic evaluation [94]. Gravimetric capacitance alone should not be used as the principal indicator of performance. Areal capacitance, volumetric capacitance, areal energy density, volumetric energy density, power density, rate capability, equivalent series resistance, coulombic efficiency, self-discharge, and long-term cycling stability should be considered together. For wearable MSCs, mechanical retention under repeated deformation and performance under realistic environmental conditions should also be reported. Comparisons should preferably use full-device values rather than electrode-level measurements and should clearly state the mass, volume, footprint, electrolyte, voltage window, and measurement conditions used for calculation. Overall, the path toward high-performance MSCs is best understood as a multidimensional optimization problem rather than a search for a single exceptional material. Hierarchical porosity can improve ion accessibility, conductive networks can accelerate electron transport, pseudocapacitive phases can increase charge storage, advanced electrolytes can expand the operating voltage, and three-dimensional or multilayer architectures can increase practical active-material loading. When these strategies are integrated through rational electrode–electrolyte and device-level design, MSCs can simultaneously achieve high energy density, high power density, rapid charge-discharge capability, long cycle life, and mechanical adaptability. The next generation of MSC research should therefore move beyond isolated record values toward realistic, reproducible, scalable, and application-relevant performance, particularly for wearable electronics where electrochemical, mechanical, geometric, and manufacturing requirements must all be satisfied simultaneously.

**Figure 8:**
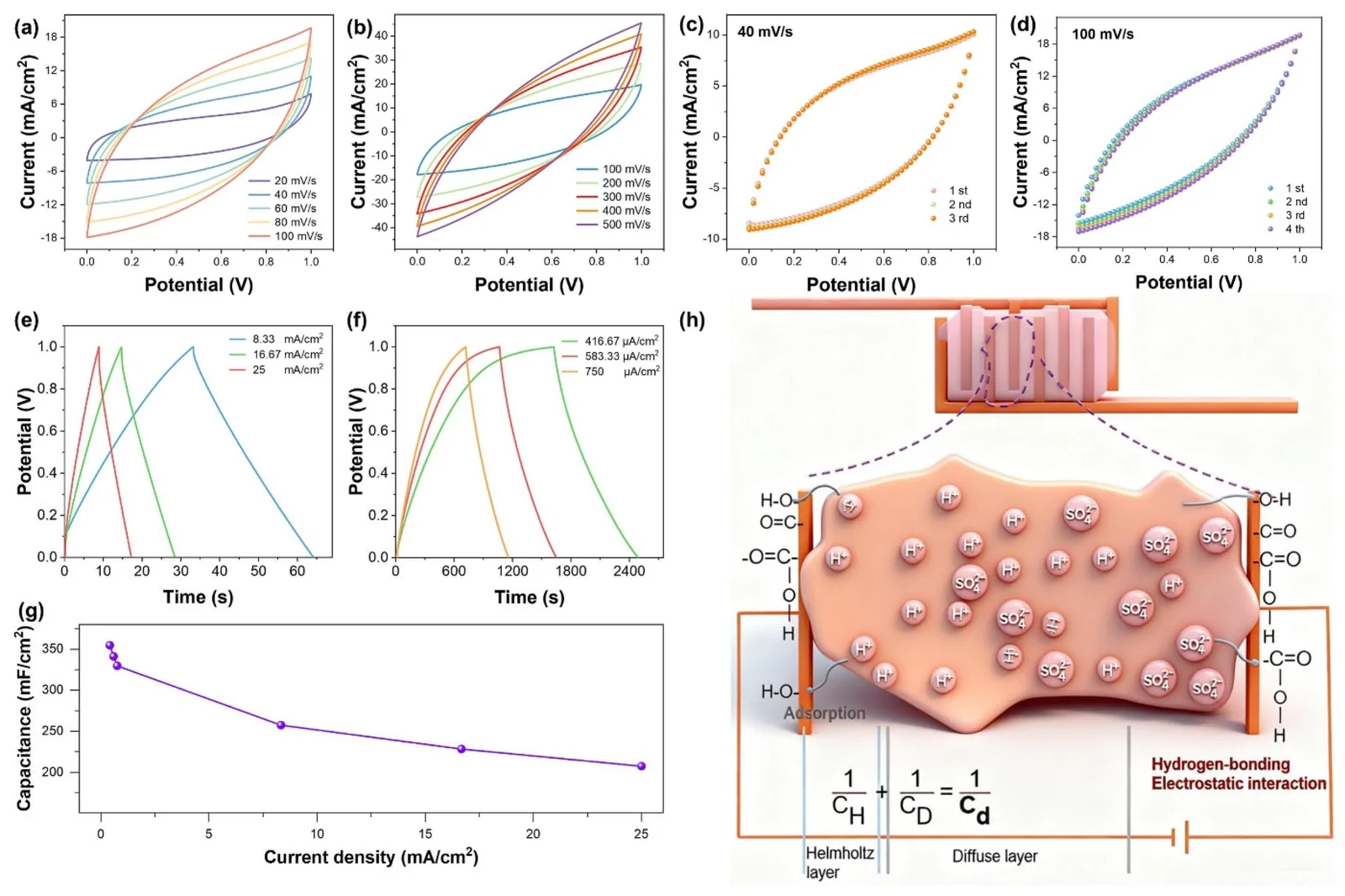
Electrochemical behavior and charge-storage mechanism of the MSC: CV curves at (**a**) low and (**b**) high scan rates, including representative CV profiles at (**c**) 40 and (**d**) 100 mV/s; GCD curves at (**e**) high and (**f**) low current densities; (**g**) variation in specific capacitance with current density; and (**h**) schematic illustration of the MSC charge-storage mechanism. Reproduced with permission from Ref. [92]. Copyright (2026) Elsevier.

Yao et al. developed a high-performance flexible microsupercapacitor (MSC) capable of stable and efficient charge–discharge operation under exceptionally high current densities, addressing a major limitation of conventional MSCs for applications requiring rapid energy delivery and recovery [92]. The device achieved an ultra-high charging current density of 25 mA cm^−2^ while maintaining high areal capacitance and stable electrochemical behavior over a broad current-density range of 0.41–25.00 mA cm^−2^. The high-rate performance was attributed to the synergistic optimization of the electrode structure and electrode–electrolyte interface. Specifically, activated carbon (AC) was integrated with graphite paper (GP) through chemical bonding, combining the high charge-storage capability and porous structure of AC with the excellent in-plane electrical conductivity of GP. This architecture facilitates efficient electron transport while maintaining accessible electrochemical surface area for ion storage. In addition to electrode optimization, the authors introduced interface engineering to promote rapid ion transport from the electrolyte toward electrochemically active sites, thereby reducing ionic transport limitations and interfacial polarization at high current densities. The optimized device demonstrated a high areal capacitance of 207.50 mF cm^−2^ under the reported operating conditions and exhibited efficient constant-current charging and discharging across an unusually wide current-density window. The study is particularly significant because many MSCs demonstrate attractive capacitance at relatively low current densities but experience substantial voltage loss, polarization, and capacitance degradation when subjected to rapid charging. By addressing electron conduction, ion transport, and interfacial stability simultaneously, this work illustrates a system-level strategy for overcoming the rate-performance limitations of flexible MSCs. The ability to operate at 25 mA cm^−2^ is especially relevant to wearable electronics, implantable microsystems, and miniaturized pulse-power sources, where energy may need to be stored or released within very short time intervals (Figure 8a–h). More broadly, the work demonstrates that achieving practical high-power MSCs requires simultaneous optimization of electrode architecture and interfacial chemistry rather than relying solely on increasing the intrinsic capacitance of the active material. This study therefore provides an important design framework for developing flexible MSCs capable of ultrafast charging, high-current operation, and efficient energy recovery in next-generation wearable and miniaturized electronic systems.

## 10. Integration with Next-Generation Wearable Electronics

The rapid evolution of wearable electronics from conventional fitness trackers toward continuous health monitoring, electronic skin, smart textiles, soft robotics, implantable systems, and autonomous sensor platforms has created an increasing demand for miniature energy-storage technologies that can operate reliably under dynamic mechanical and environmental conditions [72,82]. MSCs are particularly attractive for these applications because of their high-power density, rapid charge-discharge capability, long cycle life, compact dimensions, and compatibility with microfabrication and flexible substrates. Nevertheless, the successful integration of MSCs into next-generation wearable electronics requires a transition from the development of isolated high-performance devices toward the design of integrated energy-storage systems in which the MSC, energy harvester, sensor, signal-processing circuit, wireless communication unit, and substrate operate as a coordinated platform. In this context, the role of MSCs is not necessarily to replace batteries in every wearable system, but rather to provide rapid power delivery, buffer intermittent energy, stabilize voltage, and support high-frequency operation of low-power electronic components. Wearable health-monitoring systems represent one of the most important application areas for integrated MSCs. Modern wearable platforms can incorporate sensors for monitoring physiological and biochemical parameters, including temperature, motion, pressure, strain, respiration, and various biomarkers [52]. Many of these sensors require relatively low average power but may generate short-duration power demands during data acquisition, processing, or wireless transmission. MSCs are well suited to such load profiles because they can deliver high current over short periods and can be rapidly recharged. An integrated MSC can therefore function as a local energy buffer positioned close to the sensor, minimizing power-delivery losses and reducing dependence on bulky external batteries [33,51]. Flexible MSCs fabricated directly on polymeric substrates can further conform to the body, improving the mechanical compatibility of the complete wearable platform. Electronic skin represents an even more demanding application because it requires distributed arrays of sensors capable of detecting pressure, strain, temperature, and tactile stimuli over large and deformable surfaces. In such systems, centralized batteries may introduce rigidity and restrict the spatial distribution of electronics. Miniaturized MSC arrays can instead be distributed across the electronic-skin platform, supplying local power to individual sensor nodes or groups of sensors. The small footprint of MSCs enables integration without significantly compromising the mechanical compliance of the electronic skin. Stretchable architectures based on serpentine conductors, porous electrodes, elastomeric substrates, and deformable electrolytes can further enable energy storage under repeated stretching and compression. Smart textiles provide another major opportunity for MSC integration. Textile-based electronics require energy-storage components that can withstand bending, twisting, stretching, folding, and, potentially, washing and abrasion. Fiber-shaped MSCs and textile-integrated MSCs can be incorporated directly into yarns, fabrics, or garments, creating distributed energy-storage networks. Such systems could power wearable sensors, lighting elements, communication modules, or low-power displays. The principal advantage of textile integration is that the energy-storage function can become part of the fabric rather than an externally mounted component. However, practical implementation requires highly durable electrode-fiber interfaces, environmentally stable electrolytes, mechanically robust encapsulation, and manufacturing techniques compatible with textile production. Self-powered wearable systems represent another important direction in which MSCs can play a complementary role. Energy harvesters based on triboelectric, piezoelectric, photovoltaic, thermoelectric, or biochemical mechanisms often generate intermittent or fluctuating electrical power. Directly connecting these harvesters to electronic loads can result in unstable operation because the generated power may not match the instantaneous requirements of the device. An MSC can act as an intermediate energy buffer, storing harvested energy and subsequently delivering it when required. This combination of energy harvesting and microscale energy storage can enable autonomous wearable systems that operate without frequent external charging. The high power capability and rapid response of MSCs are particularly advantageous for smoothing intermittent energy input and supporting transient loads.

The integration of MSCs with flexible and organic electronics also offers significant opportunities [72,93]. Organic transistors, flexible displays, printed sensors, and thin-film circuits can be fabricated on polymeric or paper-based substrates, creating an opportunity for energy storage to be fabricated directly on the same platform. In such systems, compatible processing temperatures and solvents become critical. High-temperature electrode synthesis may damage polymeric electronic components, while aggressive solvents may degrade flexible substrates. Consequently, low-temperature deposition, printing, laser processing, and solution-based fabrication methods are particularly attractive for integrated wearable electronics. Solid-state and gel electrolytes further simplify integration by reducing leakage and eliminating the need for conventional liquid-electrolyte packaging. Wireless and Internet-of-Things-enabled wearable devices provide another important application scenario. A wearable sensor may spend most of its operating time in a low-power state but periodically require a substantially higher current for wireless data transmission [92]. MSCs can supply these short-duration current pulses while allowing the average energy demand to be supplied through an external battery or energy-harvesting source. This battery-MSC hybridization can potentially reduce the high-current stress imposed on batteries, improving system-level power management and potentially extending battery lifetime. In future autonomous wearable networks, distributed MSCs may therefore serve as local power buffers positioned close to individual sensing and communication modules. The integration of MSCs with microelectronic circuits also requires careful consideration of voltage matching. Individual MSC cells may operate at relatively low voltages, particularly when aqueous electrolytes are employed. Connecting multiple MSCs in series can increase the overall voltage, whereas parallel configurations increase capacitance and current capability. Series-parallel MSC arrays can therefore be designed to match the voltage and current requirements of specific electronic components. However, voltage balancing becomes increasingly important as the number of cells increases because variations in capacitance, leakage current, and internal resistance can lead to uneven voltage distribution. Integrated balancing circuits or carefully controlled device fabrication may therefore be required for reliable operation of larger MSC arrays. For wearable applications, physical integration is equally important as electrical integration. An MSC attached to a flexible electronic platform must maintain mechanical compatibility with the surrounding components. Large differences in elastic modulus can produce localized stress during bending or stretching, potentially resulting in delamination or electrical failure. Accordingly, energy-storage components should ideally be fabricated directly on compatible substrates or integrated through mechanically compliant interconnects. Stretchable conductors, serpentine architectures, conductive textiles, and elastomeric encapsulation can help distribute strain throughout the system [52,81]. In addition, the MSC should maintain stable electrochemical characteristics under realistic deformation rather than only under static laboratory conditions.

Thermal management and environmental stability also become increasingly important as wearable systems become more compact and densely integrated. Although MSCs generally exhibit favorable power characteristics and can operate with relatively low heat generation, repeated rapid charging and discharging can produce resistive losses. In densely integrated systems, heat accumulation may affect both the MSC and neighboring electronic components. Similarly, humidity, sweat, temperature fluctuations, and mechanical abrasion can alter electrolyte and electrode properties. Encapsulation strategies must therefore protect the electrochemical components while maintaining flexibility and, where necessary, breathability and skin compatibility. The development of multifunctional wearable platforms will increasingly require MSCs with additional capabilities beyond energy storage. Self-healing electrolytes and electrodes could improve operational lifetime after mechanical damage, while biodegradable or environmentally benign materials could support transient or sustainable wearable electronics. Transparent MSCs may enable integration with displays and optoelectronic devices, while ultrathin MSCs could be incorporated into electronic skins without significantly affecting their tactile response. Fiber-shaped and textile-compatible devices could provide distributed energy storage across garments. These developments suggest that future MSCs will increasingly become multifunctional structural components rather than independent energy-storage units. Despite these opportunities, several challenges remain before MSCs can be widely integrated into commercial wearable electronics. The most important include achieving sufficiently high practical energy density, maintaining performance under repeated mechanical deformation, developing environmentally stable electrolytes, ensuring reliable packaging, reducing fabrication costs, improving manufacturing yield, and establishing standardized methods for evaluating integrated devices. Importantly, laboratory reports frequently emphasize electrode-level capacitance or short-term cycling performance, whereas wearable applications require system-level metrics such as energy delivered to the actual load, operational lifetime, mechanical reliability, charging time, leakage, standby behavior, and environmental durability. Future studies should therefore increasingly evaluate MSCs under realistic device operating conditions. The integration of MSCs with next-generation wearable electronics ultimately represents a shift from stand-alone microscale energy storage toward distributed and multifunctional power-management platforms [72,93]. MSCs can provide rapid power delivery, compensate for intermittent energy harvesting, support wireless communication pulses, and stabilize power supply to sensors and microelectronic circuits. Their small dimensions and compatibility with flexible, stretchable, fiber-shaped, and textile architectures make them particularly attractive for body-conformal electronics. However, realizing their full potential will require simultaneous advances in materials, microfabrication, architecture, packaging, power management, and system integration. The most successful future platforms will likely combine MSCs with energy harvesters, batteries, sensors, and low-power electronics in highly integrated architectures that are mechanically adaptive and environmentally robust. Such integration could enable autonomous electronic skins, smart garments, continuous health-monitoring systems, soft robotic interfaces, and distributed wearable sensor networks, positioning MSCs as an important enabling technology for the emerging generation of intelligent, self-powered, and human-compatible electronics.

## 11. Advanced and Emerging MSC Systems

The rapid evolution of MSCs is shifting the field beyond conventional planar electric-double-layer devices toward multifunctional, adaptive, self-powered, and highly integrated microscale energy-storage systems [36,51]. The emerging generation of MSCs is being developed not simply to maximize capacitance or power density, but to satisfy the increasingly complex requirements of wearable electronics, autonomous sensors, electronic skins, smart textiles, implantable microsystems, and Internet-of-Things platforms. Recent research demonstrates a clear movement toward flexible and stretchable devices, self-healing systems, electrochromic MSCs, high-voltage architectures, fiber-shaped devices, hybrid micro-energy-storage systems, and MSC-powered integrated sensing platforms [23,65]. This transition represents an important conceptual change in which the MSC is increasingly regarded as a multifunctional component of an integrated microsystem rather than an isolated energy-storage unit. One of the most important emerging directions is the development of multifunctional MSCs in which energy storage is combined with another useful function. Conventional MSCs are designed primarily to store and deliver electrical energy, whereas multifunctional systems can simultaneously provide electrochromic, sensing, self-healing, thermal-management, or mechanical functions. Electrochromic MSCs are particularly interesting because their optical state can provide a visual indication of the stored charge, potentially eliminating the need for an independent battery-status indicator [68]. The integration of electrochromic materials with micro-supercapacitor electrodes can therefore produce energy-storage devices that are simultaneously active and visually responsive. Such systems are particularly attractive for smart windows, wearable displays, optical sensors, and low-power human-machine interfaces. Self-healing MSCs represent another important emerging class designed to address mechanical damage in flexible and wearable electronics. Conventional electrodes and electrolytes may develop cracks, delamination, or localized defects during repeated bending and stretching, eventually resulting in irreversible performance degradation. Self-healing systems attempt to restore electrical and ionic pathways through reversible chemical bonding, hydrogen bonding, metal-ligand interactions, host-guest interactions, ionic interactions, or dynamic covalent chemistry. Self-healing polymer electrolytes are especially promising because the electrolyte can simultaneously maintain ionic conductivity and repair mechanical damage. The development of self-healing electrodes, however, remains more challenging because electronic conductivity must be restored while maintaining electrochemical activity. Consequently, future systems may increasingly employ dynamically crosslinked conductive networks in which mechanical recovery and electrochemical functionality are intrinsically coupled. Multifunctional flexible and self-healing MSCs have already emerged as a distinct direction within advanced on-chip energy storage.

MXene-based MSCs represent one of the most rapidly developing material platforms in this area. MXenes combine high electrical conductivity, two-dimensional morphology, hydrophilic surfaces, tunable surface terminations, and redox-active characteristics, making them particularly suitable for high-performance microelectrodes. Their interlayer spacing, surface chemistry, defects, vacancies, and pore structures can be engineered to control ion transport and electrochemical activity. However, nanosheet restacking and environmental oxidation remain significant limitations. Recent strategies therefore focus on interlayer expansion, surface functionalization, incorporation of carbon nanomaterials, conducting polymers and metal compounds, and construction of three-dimensional porous MXene networks. Emerging MXene-based MSCs are also being integrated directly with sensing systems, demonstrating a transition from material-level energy storage toward multifunctional microsystem platforms. The development of high-voltage MSCs is another critical research direction because the energy density of a capacitor increases with the square of its operating voltage [68,75]. Conventional aqueous MSCs offer high ionic conductivity but are limited by water electrolysis, restricting the attainable voltage window. Organic electrolytes, ionic liquids, and solid-state ionic systems can provide wider electrochemical stability windows and consequently enable higher-energy microscale devices. However, increasing the voltage introduces additional challenges related to electrolyte decomposition, electrode oxidation or reduction, interfacial instability, leakage, and safety. The future development of high-voltage MSCs will therefore require coordinated optimization of electrode potentials, electrolyte stability, interfacial chemistry, and device encapsulation rather than simply replacing an aqueous electrolyte with a higher-voltage medium.

Solid-state and quasi-solid-state MSCs are particularly important for next-generation wearable systems because they eliminate many of the limitations associated with liquid electrolytes. Polymer electrolytes, gel electrolytes, hydrogels, ionogels, and ionic-liquid-containing solid matrices can provide ionic conductivity while maintaining a mechanically stable device configuration. These electrolytes also reduce leakage and facilitate fabrication of ultrathin, flexible, stretchable, and fiber-shaped MSCs. More advanced solid-state systems are being designed to provide additional characteristics such as self-healing, antifreezing behavior, adhesion, high-temperature stability, and resistance to dehydration. The integration of multifunctional electrolytes with advanced electrode architectures is therefore likely to become one of the major routes toward mechanically robust wearable MSCs. Recent research on MXene-based solid-state systems, for example, highlights the importance of addressing restacking, oxidation, electrolyte compatibility, and interfacial resistance simultaneously. Three-dimensional and vertically integrated MSCs represent another major emerging technology. Conventional planar interdigitated MSCs provide excellent power capability because of short ion-transport distances, but their limited electrode thickness can constrain practical energy density. Three-dimensional architectures overcome this limitation by increasing the amount of electrochemically active material within a confined footprint. Vertically aligned nanowires, nanosheets, porous carbon frameworks, micro-pillars, nanoporous scaffolds, and hierarchical architectures can provide interconnected electronic pathways while maintaining efficient electrolyte penetration. The most promising structures combine macroporous channels for electrolyte transport with mesoporous and microporous regions for charge storage. Such multiscale architectures provide a pathway toward simultaneously increasing areal, volumetric, and power performance.

Fiber-shaped and textile-integrated MSCs are also becoming increasingly important for wearable electronics. Fiber architectures provide intrinsic flexibility and can be bent, twisted, woven, knitted, or embroidered into textiles [72]. Coaxial and twisted fiber MSCs can be fabricated using carbon fibers, metallic fibers, conductive polymers, graphene, carbon nanotubes, and MXene-based materials. The integration of energy storage directly into fibers enables smart garments in which the energy-storage component becomes part of the textile structure. However, practical textile integration requires more than electrochemical performance; the devices must withstand repeated deformation, abrasion, washing, humidity, and mechanical fatigue. Recent work on flexible fiber-based supercapacitors emphasizes that electrolyte stability, mechanical durability, energy density, and textile integration remain closely coupled challenges. An especially promising direction is the emergence of MSC-energy-harvester hybrid systems. Wearable energy harvesters based on triboelectric, piezoelectric, photovoltaic, thermoelectric, or biochemical mechanisms can generate electrical energy from environmental or physiological sources, but their output is often intermittent and insufficiently stable for direct operation of electronic loads. MSCs can function as rapid-response energy buffers that store harvested energy and subsequently provide short-duration power pulses. This configuration can enable self-charging wearable systems in which the energy-storage device continuously receives energy from the surrounding environment [23]. Such architectures are particularly attractive for autonomous sensors and low-power Internet-of-Things devices, where minimizing or eliminating conventional battery replacement is highly desirable. Flexible MSC research has already demonstrated integration concepts involving energy harvesting, sensing, and self-powered operation.

The practical suitability of an MSC should ultimately be evaluated against the electrical requirements of the target wearable system rather than from energy or power density alone. Wearable sensors may operate at relatively low average power while requiring short-duration power pulses during sensing, signal processing, or wireless transmission. Therefore, the voltage output, instantaneous power capability, energy available per operating cycle, and duty cycle of the MSC should be considered together with the load requirement. In this context, MSCs can function as local power buffers or transient-power sources, while batteries or energy harvesters may provide the average energy demand [22,69].

The integration of MSCs with self-powered sensing systems is arguably one of the most important emerging developments [61,93]. In such systems, the MSC is not treated as an independent power source but as one component within a closed energy pathway consisting of an energy harvester, energy-storage unit, sensor, signal-processing circuit, and communication module. MXene-based MSCs have recently been investigated specifically for integrated sensing systems because their electrochemical properties can be combined with the multifunctionality of MXene-based sensors. Such integration can enable continuous detection of mechanical strain, temperature, chemical species, or physiological signals while using the MSC as a local power source or energy buffer. Another emerging concept is the structural or multifunctional MSC, in which the energy-storage component contributes to the physical structure of the electronic system. Rather than adding an independent battery or capacitor to a wearable device, the electrode, substrate, textile, or structural framework can simultaneously perform mechanical support and energy storage [46,98]. This concept is particularly attractive for flexible robotics, electronic skins, smart textiles, and lightweight autonomous systems. Conductive fibers, porous carbon scaffolds, polymer networks, and MXene-hydrogel composites can potentially provide both mechanical adaptability and electrochemical functionality. Recent developments in MXene-hydrogel systems illustrate the growing interest in combining electrochemical, mechanical, self-healing, and shape-conforming characteristics within a single platform.

Biodegradable, transient, and environmentally sustainable MSCs represent another emerging research direction. Conventional microelectronic devices can generate electronic waste because their substrates, electrolytes, current collectors, and active materials persist after disposal. Biodegradable polymers, cellulose-derived carbon materials, transient metals, aqueous electrolytes, and bio-derived electrode materials offer potential routes toward environmentally responsible microscale energy storage. For temporary biomedical sensors, disposable diagnostic systems, and transient electronics, complete or partial biodegradability could be particularly advantageous. Nevertheless, sustainability should be assessed across the complete life cycle, including material synthesis, processing energy, solvent consumption, device lifetime, and end-of-life behavior, rather than simply based on the use of a bio-derived electrode. Artificial intelligence and computational materials design are also beginning to influence the development of advanced MSCs. The enormous parameter space involving composition, pore structure, surface chemistry, electrolyte formulation, electrode thickness, microelectrode geometry, and fabrication conditions makes conventional experimental optimization inefficient. Machine-learning models can potentially establish relationships between material descriptors, processing parameters, structural characteristics, and electrochemical performance. Recent research on advanced MXene-based solid-state systems has specifically identified machine learning as a promising approach for accelerating materials discovery and optimization. In the longer term, data-driven approaches could enable inverse design, in which the desired MSC energy and power characteristics are specified first and the optimal electrode–electrolyte -architecture combination is predicted computationally. Ultimately, the most important emerging trend is the transition from isolated high-performance MSCs toward intelligent, multifunctional, and system-integrated microscale power sources [18,36]. Advanced MSCs are increasingly expected to combine high energy and power densities with flexibility, stretchability, self-healing, high-voltage operation, environmental stability, sensing capability, optical functionality, and compatibility with energy harvesting. This development is particularly relevant to wearable electronics, where the energy-storage unit must operate under continuous mechanical deformation while interacting with sensors and communication electronics. The field is consequently moving toward a systems-engineering paradigm in which materials, electrolytes, microfabrication, architecture, mechanical design, and electronics are optimized simultaneously. Recent literature increasingly emphasizes this shift from device-centric optimization toward integrated microsystem functionality. For the future, the most transformative MSC platforms will likely combine several emerging concepts within a single architecture-for example, a three-dimensional MXene-carbon electrode coupled with a self-healing solid-state electrolyte, fabricated by scalable printing or laser patterning and integrated with an energy harvester and wearable sensor. Such convergence could enable autonomous, mechanically adaptive, and multifunctional microsystems capable of sensing, processing, communicating, and storing energy within an ultracompact platform. The ultimate challenge is therefore no longer merely to achieve another record capacitance value, but to establish MSCs that deliver reproducible system-level performance under realistic mechanical, thermal, environmental, and operational conditions. This transition from record-oriented materials research to application-driven multifunctional microsystem engineering is likely to define the next major stage of MSC development.

**Figure 9:**
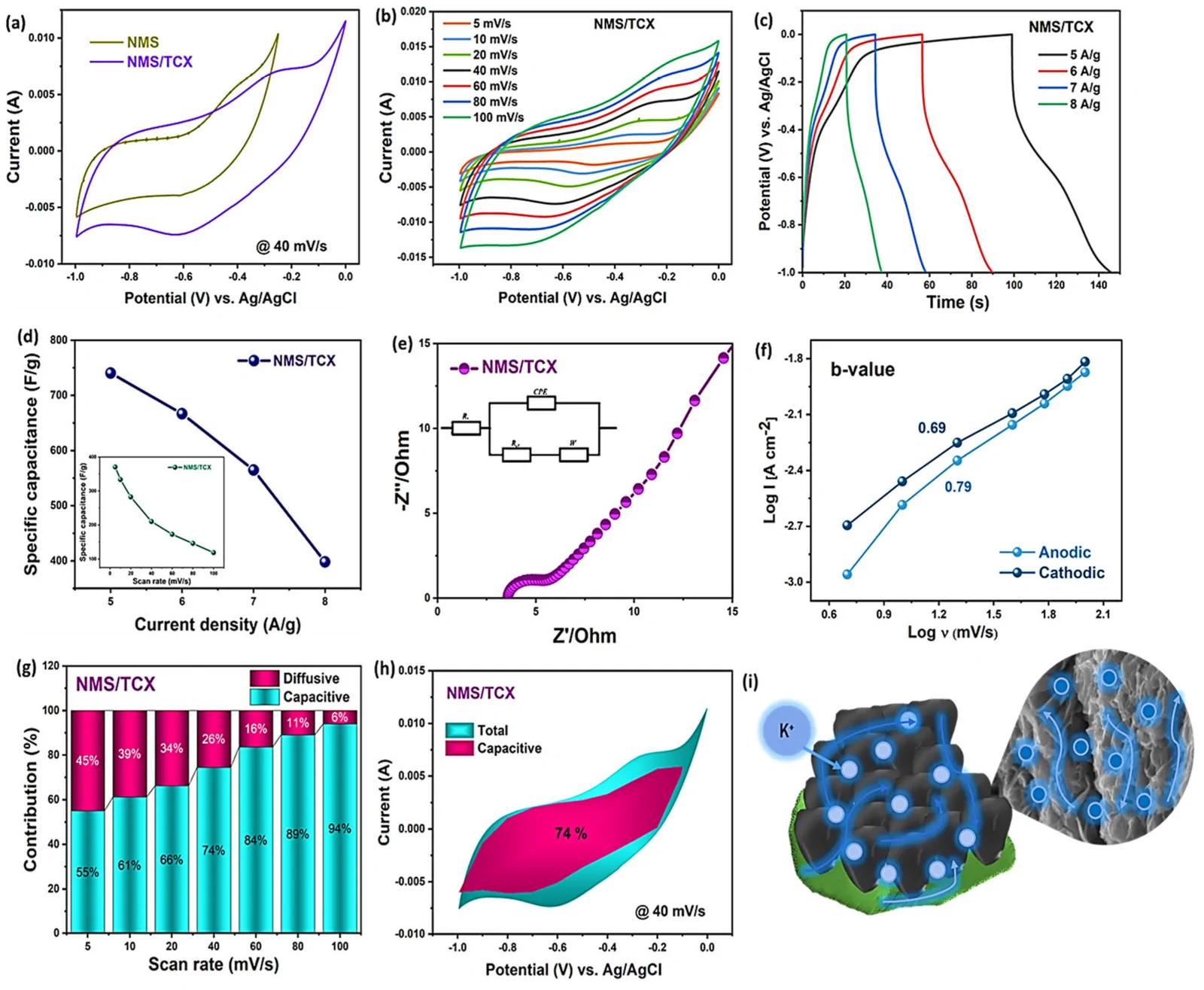
Electrochemical performance and charge-storage kinetics of pristine NMS and NMS/TCX electrodes in 0.5 M K_2_SO_4_: (**a**) comparative electrochemical performance; (**b**) CV curves of the NMS/TCX electrode at various scan rates; (**c**) GCD profiles at different current densities; (**d**) specific capacitance as a function of current density (inset: specific capacitance versus scan rate); (**e**) Nyquist plot with the equivalent-circuit inset; (**f**) log(scan rate) versus log(current) plot; (**g**) capacitive and diffusion-controlled contribution ratios at various scan rates; (**h**) capacitive contribution at 40 mV/s; and (**i**) schematic illustration of the charge-storage mechanism. Reproduced with permission from Ref. [83]. Copyright (2025) The Royal Society of Chemistry.

Pathak et al. reported a simple and scalable strategy for developing high-performance flexible microsupercapacitors (MSCs) by integrating hierarchical Ni–Mo–S nanostructures with Ti_3_C_2_T_x_ MXene on laser-scribed graphene (LSG) microelectrodes [83]. The central design principle of this work is the construction of a hybrid electrode architecture in which the complementary properties of MXene and Ni–Mo–S are utilized to overcome the limitations associated with individual electrode materials. Ti_3_C_2_T_x_ MXene provides high electrical conductivity, hydrophilic surface chemistry, and a two-dimensional framework that facilitates electrolyte interaction, whereas the Ni–Mo–S nanostructures introduce additional redox-active sites and hierarchical interfaces for enhanced pseudocapacitive charge storage. The hybrid Ni–Mo–S/Ti_3_C_2_T_x_ structure was synthesized directly on ultrathin MXene through a one-step hydrothermal process, providing intimate interfacial contact between the two components and promoting efficient electron and ion transport. The resulting hybrid was subsequently integrated with micropatterned laser-scribed graphene to construct a planar solid-state MSC. The ultrathin electrode configuration, interconnected two-dimensional conductive framework, and favorable electrolyte wettability collectively enabled efficient utilization of the electroactive material. The optimized LSG-Ni–Mo–S/Ti_3_C_2_T_x_ MSC delivered an areal capacitance of 208 mF cm^−2^ at 5 mA cm^−2^ and maintained a high rate capability of 94.6% after 10,000 charge–discharge cycles. More importantly, the device achieved an areal energy density of 65.10 μWh cm^−2^ at a power density of 4212 μW cm^−2^, demonstrating a favorable balance between energy-storage capability and high-rate power delivery. The MSC also retained its electrochemical functionality under different bending and twisting configurations, highlighting the mechanical compatibility of the hybrid electrode architecture with flexible electronics. From a materials-design perspective, this study demonstrates the effectiveness of combining a highly conductive two-dimensional MXene scaffold with hierarchical transition-metal sulfide nanostructures to increase electrochemically accessible interfaces while maintaining efficient charge transport (Figure 9a–i). From a device-engineering perspective, the integration with laser-scribed graphene provides a relatively simple route toward micropatterned planar MSC fabrication. Overall, the work illustrates how hierarchical heterostructure design, interfacial coupling, and direct microfabrication can be simultaneously exploited to develop flexible MSCs with high areal energy density, high power capability, excellent cycling stability, and mechanical flexibility, making this architecture highly promising for next-generation wearable and miniaturized electronic systems.

## 12. Reliability, Safety and Sustainability

The transition of MSCs from laboratory-scale demonstrations to practical components of next-generation wearable and microscale electronics requires considerably greater attention to reliability, safety, and sustainability [36]. High capacitance, rapid charge–discharge capability, and long cycling life are important performance indicators, but they do not by themselves establish technological viability. MSCs intended for wearable electronics must maintain their electrochemical, electrical, and mechanical characteristics over prolonged operation while being exposed to repeated bending, stretching, temperature fluctuations, humidity, perspiration, mechanical abrasion, and intermittent charging. At the same time, the materials and fabrication processes should minimize environmental and health risks throughout the device life cycle. These considerations are particularly important because MSCs typically contain multiple chemically and mechanically dissimilar components, including conductive substrates, electroactive materials, electrolytes, current collectors, binders, separators or solid-state matrices, and encapsulation layers. Failure of any individual component or interface can compromise the performance of the complete microsystem. Consequently, reliability, safety, and sustainability should be treated as interconnected design criteria from the earliest stages of MSC development rather than as post-fabrication considerations. The reliability of an MSC is governed by the stability of its electrochemical reactions, electrode structure, electrolyte, interfaces, and packaging [93]. During repeated charge–discharge operation, electrochemical and mechanical stresses can gradually modify the electrode morphology and surface chemistry. In pseudocapacitive materials, repeated oxidation and reduction may induce volume expansion and contraction, structural reconstruction, dissolution of active species, or loss of electrical contact. Conducting polymers can undergo swelling and shrinking during ion insertion and extraction, while layered materials may experience restacking or delamination. Even nominally stable carbon electrodes can undergo surface oxidation or pore blockage under aggressive operating conditions. These processes progressively increase internal resistance and reduce capacitance. Therefore, long-term cycling stability should be evaluated using realistic current densities, voltage windows, electrolyte concentrations, and electrode loadings rather than relying exclusively on highly favorable laboratory conditions. Mechanical reliability becomes particularly important for flexible and wearable MSCs. Repeated bending, twisting, stretching, compression, and folding can generate stress concentrations at the interfaces between the substrate, current collector, electrode, electrolyte, and encapsulation layer. Delamination is one of the most common failure mechanisms because different layers may have substantially different elastic moduli and strain responses. Brittle active materials can crack even when the underlying substrate is highly flexible. Similarly, repeated deformation can alter electrode porosity and electrolyte distribution, thereby increasing ionic resistance. Structural strategies such as porous networks, nanostructuring, serpentine interconnects, wrinkled electrodes, mechanically interlocked interfaces, and elastomeric components can distribute mechanical stress and improve durability. For wearable MSCs, however, mechanical reliability should be assessed simultaneously with electrochemical cycling because mechanical and electrochemical degradation can interact [72]. Mechanical reliability should be evaluated together with the specific deformation conditions used during testing. Bending radius, tensile strain, deformation mode, number of deformation cycles, and the corresponding electrochemical retention should be reported because these parameters strongly influence the measured stability. Consequently, retention values obtained under different mechanical conditions should not be treated as directly equivalent. Standardized reporting of mechanical deformation and electrochemical retention would improve the reliability of comparisons among flexible and stretchable MSCs [52].

Electrolyte stability represents another major determinant of reliability [36]. Liquid electrolytes may evaporate, leak, or migrate during long-term operation, particularly in thin and flexible devices. Gel, hydrogel, ionogel, and solid-state electrolytes can substantially reduce these problems by immobilizing the ionic phase within a supporting matrix. However, solid-state electrolytes introduce their own challenges, including reduced ionic conductivity, dehydration, freezing, polymer degradation, and interfacial resistance. For wearable systems, electrolyte formulations must additionally tolerate exposure to humidity, body temperature, sweat, and repeated mechanical deformation. The development of antifreezing, anti-drying, self-healing, and highly adhesive electrolytes therefore represents an important strategy for improving operational reliability under realistic environmental conditions. The electrode–electrolyte interface is particularly important because many degradation processes originate at this boundary. Poor wetting can leave electrochemically inactive regions, while chemical incompatibility may result in corrosion, surface reconstruction, or parasitic reactions. High operating voltages can accelerate electrolyte decomposition and interfacial side reactions. In pseudocapacitive systems, irreversible reactions may progressively alter the oxidation state or morphology of the active material. Interfacial engineering through surface functionalization, conformal coatings, heterostructure construction, and chemically compatible electrolytes can suppress these processes. More importantly, evaluating the interface before and after long-term cycling using techniques such as X-ray photoelectron spectroscopy, electron microscopy, spectroscopy, and impedance analysis can provide valuable information about the underlying degradation mechanisms.

Thermal reliability must also be considered, particularly in densely integrated MSC arrays. Although MSCs can generally deliver high power with rapid charge–discharge, internal resistance produces Joule heating during high-current operation. At the microscale, heat dissipation can become significant because the device volume is small and neighboring electronic components may be located within a few micrometers or millimeters. Excessive temperature can accelerate electrolyte decomposition, electrode degradation, polymer aging, and substrate deformation. Consequently, reducing equivalent series resistance and incorporating thermally stable materials and packaging are important for reliable operation. Thermal characterization under realistic high-rate cycling conditions should therefore accompany conventional electrochemical testing. Safety is equally important for wearable MSCs because the energy-storage device may operate directly on or near the human body. Electrolyte leakage, chemical irritation, thermal instability, electrical short circuits, and degradation products must be carefully considered. Aqueous electrolytes generally offer favorable safety characteristics compared with many flammable organic systems, although strongly acidic or alkaline formulations can present chemical hazards. Organic electrolytes may provide wider voltage windows but can introduce flammability and toxicity concerns. Ionic liquids can reduce volatility but are not automatically environmentally benign or biologically harmless. Therefore, electrolyte selection should consider chemical composition, toxicity, volatility, electrochemical stability, and interaction with encapsulation materials. Solid-state and quasi-solid-state electrolytes can improve safety by minimizing leakage and maintaining structural integrity. Electrical safety is also closely connected with device architecture. In densely packed MSC arrays, fabrication defects or insufficient interelectrode separation can produce short circuits. Variations in capacitance and leakage current among cells connected in series can result in uneven voltage distribution, potentially driving individual cells beyond their stable operating window. Careful microfabrication, electrical isolation, voltage balancing, and quality control are therefore necessary for reliable MSC arrays [33,65]. Encapsulation can provide an additional barrier against moisture and contamination while reducing the probability of mechanical damage and electrolyte leakage. For wearable systems, however, the encapsulation layer must remain flexible and durable without introducing excessive thickness or compromising conformability.

Sustainability represents a broader challenge extending across the entire MSC life cycle. The environmental impact of an MSC is determined not only by its final composition but also by raw-material extraction, electrode synthesis, solvent use, energy-intensive processing, fabrication, operational lifetime, and end-of-life disposal. Many high-performance electrode materials require complex synthesis routes, elevated temperatures, hazardous solvents, or scarce elements [12,23]. Similarly, microfabrication processes can involve significant chemical consumption and energy demand. Consequently, improving sustainability requires consideration of the complete manufacturing chain rather than simply replacing one component with a bio-derived material. Carbonaceous materials derived from renewable resources offer an attractive pathway toward more sustainable MSCs. Cellulose, lignin, chitosan, alginate, biomass-derived carbon, and other renewable precursors can serve as carbon sources, binders, substrates, or electrolyte components. Their abundance and potential for low-cost processing make them attractive for environmentally responsible energy-storage systems. However, the use of biomass-derived materials does not automatically guarantee sustainability. High-temperature carbonization, activation using corrosive chemicals, extensive washing, and solvent-intensive purification can significantly increase the environmental footprint. Sustainable MSC design should therefore optimize both material origin and processing requirements. The replacement of critical or potentially hazardous elements is another important strategy. Earth-abundant transition metals and carbon-based materials may provide alternatives to expensive noble metals in certain applications. Water-based processing, low-temperature fabrication, solvent recovery, and additive manufacturing can further reduce energy and material consumption. Printing and laser-based manufacturing are particularly promising because they can reduce material waste through localized deposition or direct patterning. Nevertheless, the environmental advantage of these approaches should be quantified using appropriate life-cycle analyses rather than assumed solely from reduced material waste. Long device lifetime is itself a sustainability advantage. A highly durable MSC that operates for many years can potentially have a lower environmental impact per unit of delivered energy than a device requiring frequent replacement. Therefore, cycle life, mechanical durability, environmental stability, and repairability should be considered alongside material selection. Self-healing materials may further improve sustainability by extending device lifetime and reducing replacement frequency. Modular MSC architectures could also facilitate repair or replacement of individual components rather than disposal of the entire electronic system [93,96]. End-of-life management is an emerging issue for microscale energy-storage technologies. The increasing deployment of wearable electronics could generate substantial quantities of electronic waste if MSCs are incorporated into disposable sensors and smart textiles. Designing MSCs for disassembly, material recovery, recycling, or controlled degradation could reduce this burden. Biodegradable and transient MSCs may be particularly valuable for temporary medical sensors and short-term wearable devices. However, biodegradability must be evaluated under realistic environmental conditions, and degradation products should be assessed for toxicity. A sustainable MSC should ideally combine environmentally responsible materials, low-impact processing, long operational lifetime, and appropriate end-of-life management.

A major limitation in the current MSC literature is the lack of standardized reliability, safety, and sustainability metrics. Cycling stability is frequently reported as percentage capacitance retention after a specified number of cycles, but testing conditions vary substantially among studies. Similarly, mechanical stability is often demonstrated using a limited number of bending cycles without standardized deformation conditions. Future studies should report detailed mechanical strain, bending radius, deformation frequency, temperature, humidity, electrolyte composition, current density, voltage window, active-material loading, and device-level energy and power metrics. Sustainability assessments should additionally consider material toxicity, resource criticality, solvent consumption, synthesis temperature, energy demand, manufacturing yield, and end-of-life pathways. Ultimately, the future commercialization of MSCs will depend on achieving a balance between electrochemical performance, mechanical reliability, operational safety, environmental compatibility, and manufacturing sustainability [5,11]. The highest-performing MSC is not necessarily the most technologically valuable if it relies on expensive materials, hazardous electrolytes, energy-intensive synthesis, or fabrication processes that cannot be scaled safely. Conversely, a sustainable device with insufficient energy density or poor durability will have limited practical impact. The most promising approach is therefore integrated design in which materials, electrolyte chemistry, device architecture, fabrication, packaging, and end-of-life strategy are optimized simultaneously. For next-generation wearable electronics, this philosophy will be essential for developing MSCs that are not only high-performing but also safe for prolonged human interaction, mechanically reliable under realistic conditions, economically manufacturable, and environmentally responsible throughout their life cycle.

## 13. Critical Challenges and Future Perspectives

Despite substantial progress in materials chemistry, microfabrication, device engineering, and wearable electronics, microsupercapacitors (MSCs) remain largely at the stage of laboratory-scale demonstration, with several challenges still limiting their transition toward practical microsystems [16,51]. The major limitations are no longer restricted to achieving high capacitance; instead, practical MSC development requires simultaneous consideration of energy and power density, ion and electron transport, mechanical reliability, manufacturing reproducibility, safety, sustainability, and system integration. Accordingly, the remaining challenges can be considered at three interconnected levels: fundamental science, manufacturing and device engineering, and system-level and industrial translation. Progress at each level is closely dependent on the others, and isolated optimization of a single material or device parameter is unlikely to provide a complete solution.

### 13.1. Fundamental Scientific Challenges

A fundamental challenge in MSC research is the limited energy density relative to batteries, despite the high-power capability and long cycle life of supercapacitors [32,56]. This limitation becomes more pronounced at the microscale because the amount of active material that can be incorporated within a restricted footprint and thickness is inherently limited. Extremely high gravimetric capacitance values can therefore be obtained from ultrathin electrodes with very low active-material loading without necessarily providing correspondingly high device-level energy. For practical MSCs, greater emphasis should consequently be placed on areal and volumetric energy density, together with the associated loading and device dimensions. Increasing active-material loading also introduces a fundamental energy-power trade-off. Thicker electrodes can increase the quantity of stored charge but generally increase ion-transport distances and internal resistance, whereas ultrathin electrodes provide rapid ion transport but contain less active material. Hierarchical porous structures, vertically aligned architectures, and three-dimensional electrodes provide potential routes for balancing these competing requirements. Macropores can facilitate electrolyte transport, mesopores can provide accessible ion pathways, and micropores can increase charge-storage interfaces. However, optimizing pore structure requires consideration of ion size, solvation, electrolyte composition, and surface chemistry rather than simply maximizing surface area [26]. The electrode–electrolyte interface represents another important fundamental challenge. Charge storage in MSCs is strongly influenced by ion size, solvation structure, pore dimensions, surface functional groups, electrolyte concentration, and interfacial charge distribution. Under confined conditions, ion correlations, desolvation, solvent reorganization, and confinement effects can modify conventional descriptions of electric-double-layer formation. These effects become particularly relevant when electrode pores and interelectrode dimensions approach characteristic ionic length scales. Future studies should therefore combine electrochemical measurements with spectroscopy, advanced microscopy, molecular simulations, and theoretical modeling to establish more reliable relationships between electrolyte chemistry, pore architecture, and charge-storage behavior. The stability of pseudocapacitive materials is another fundamental limitation. Transition-metal oxides, sulfides, nitrides, phosphides, and conducting polymers can provide high charge-storage capability but may undergo structural deformation, dissolution, phase transformation, or loss of electrical contact during repeated redox cycling [13]. These problems can become more pronounced at high active-material loading. Rather than maximizing the quantity of redox-active material, future designs should focus on achieving high electrochemical utilization while maintaining structural integrity through ultrathin conformal coatings, heterostructures, conductive scaffolds, defect engineering, and stabilized interfaces [17]. Electrolyte selection further couple’s energy density with stability and safety. Increasing the operating voltage can substantially increase stored energy, but high-voltage organic, ionic-liquid, deep-eutectic, and solid-state electrolytes may introduce concerns related to decomposition, corrosion, interfacial instability, self-discharge, and safety. Therefore, future electrolyte development should focus not only on extending the voltage window but also on achieving stable electrode–electrolyte interfaces and long-term operation under realistic conditions.

### 13.2. Manufacturing and Device-Engineering Challenges

The translation of laboratory MSC designs into reproducible devices requires simultaneous control of electrode geometry, active-material loading, interfaces, packaging, and fabrication processes. Conventional lithographic approaches provide high pattern resolution but may involve multiple processing steps, specialized equipment, and relatively high fabrication costs. Printing, laser patterning, electrodeposition, and additive manufacturing offer attractive alternatives for patterned and potentially large-area fabrication, but their practical scalability depends on more than the fabrication principle itself. Pattern fidelity, ink stability, thickness uniformity, registration accuracy, manufacturing yield, device-to-device variation, and batch-to-batch reproducibility must also be controlled [18,23]. A major requirement for future manufacturing studies is therefore a shift from reporting the performance of the best individual device toward reporting statistical performance across multiple devices and batches. Manufacturing yield, variation in capacitance and resistance, dimensional uniformity, and long-term stability should become routine reporting parameters. Roll-to-roll processing, digitally controlled deposition, low-temperature fabrication, and hybrid manufacturing strategies may help address these requirements, but their suitability must be demonstrated experimentally rather than assumed from process compatibility alone. Device architecture also involves important compromises. Planar interdigitated MSCs provide short ion-transport pathways and are well suited to on-chip integration, but their electrode spacing and geometry can limit active-material loading. Sandwich-type architectures can accommodate greater electrode thickness and active-material loading, but longer ion-transport pathways may increase internal resistance and reduce high-rate utilization. Vertical and three-dimensional structures can increase the accessible active surface and volumetric utilization, although they generally require more complex fabrication and structural control. Thus, architecture selection should be guided by the balance among ion-diffusion distance, areal loading, volumetric performance, device thickness, mechanical requirements, and fabrication complexity rather than by a single electrochemical metric. Mechanical reliability presents an additional device-engineering challenge for flexible and stretchable MSCs. Repeated bending, stretching, twisting, and compression can produce cracking in active layers, delamination at electrode–current-collector interfaces, changes in electrode porosity, and redistribution of gel or polymer electrolytes. These structural changes can subsequently alter interfacial resistance and charge-transfer behavior. Consequently, mechanical stability should be evaluated together with electrochemical performance, preferably through simultaneous mechanical and electrochemical testing rather than only by measuring capacitance after deformation. Mechanical testing should also report deformation mode, bending radius or strain, number of cycles, and the corresponding electrochemical retention to enable meaningful comparison among devices. Standardized performance evaluation remains closely connected to device engineering. Reported MSC performance is difficult to compare because studies use different electrode masses, voltage windows, current densities, substrates, electrolytes, device dimensions, and calculation procedures. Electrode-level properties are also sometimes presented without clearly distinguishing them from complete-device performance. Future studies should therefore report areal and volumetric metrics together with the normalization basis, active-material loading, device dimensions, voltage window, cycling conditions, mechanical testing conditions, leakage, self-discharge, and environmental stability. Such standardized reporting would improve the reliability of comparisons and help identify whether an apparent performance improvement originates from materials design, device architecture, or testing conditions.

### 13.3. System-Level and Industrial Translation Challenges

The final challenge is to demonstrate that MSCs can provide useful functions within complete electronic systems rather than only exhibiting high electrochemical performance under laboratory conditions. Wearable electronics may contain sensors, processors, transistors, wireless communication units, and energy harvesters, each with different voltage, current, and duty-cycle requirements. A sensor may require relatively low average power but experience short-duration current demand during signal processing or wireless transmission [93]. MSCs are therefore particularly attractive as local power buffers or transient-power sources, while batteries and energy harvesters can provide the average energy requirement. Future demonstrations should evaluate MSCs under realistic electronic load profiles and investigate MSC–battery hybrids, MSC–energy-harvester systems, and distributed MSC arrays. System integration also requires attention to voltage matching, power-management circuitry, packaging, environmental stability, and long-term reliability. Increasing the number of MSCs in series can provide a higher operating voltage, but the resulting reduction in equivalent capacitance, additional interconnections, footprint, and balancing requirements must be considered when evaluating complete arrays. Similarly, integration with flexible substrates and wearable systems requires reliable electrical contacts and encapsulation without compromising mechanical compliance. Safety and sustainability are also important for industrial translation. The use of aqueous or solid-state electrolytes, abundant carbon precursors, cellulose, lignin, chitosan, alginate, recyclable substrates, and low-temperature processing can potentially reduce environmental impact. However, sustainability cannot be assessed from the material source alone because biomass-derived materials may require energy-intensive carbonization, activation, or purification. Future studies should therefore consider life-cycle assessment, toxicity, resource availability, solvent consumption, manufacturing energy, recyclability, and end-of-life behavior. Designing MSCs for repair, recycling, controlled degradation, or biodegradation may become increasingly important for disposable wearable and biomedical electronics. Another emerging direction is multifunctional MSC integration. Future devices may combine energy storage with sensing, self-healing, electrochromism, thermal management, structural support, and energy harvesting [103,104,105]. Self-healing electrodes and electrolytes may improve operational durability, while integrated sensors could provide information about strain, temperature, chemical exposure, and device health. Computational modeling and machine-learning approaches may further assist materials selection, electrode-geometry optimization, degradation prediction, and process–structure–performance analysis. However, these approaches should complement experimental validation rather than replace it.

### 13.4. Outlook

The future development of MSCs will depend on the convergence of materials science, interfacial engineering, device architecture, manufacturing, mechanical design, and system integration. At the fundamental level, greater understanding of confined ion transport, electrode–electrolyte interactions, and degradation mechanisms is required. At the manufacturing level, reproducible processing, statistical quality control, pattern fidelity, and scalable fabrication must be established. At the system level, MSCs must demonstrate reliable operation under realistic electrical, mechanical, environmental, and application-specific conditions. Therefore, future research should move beyond isolated record values and focus on balanced combinations of areal and volumetric energy density, power capability, cycling stability, mechanical durability, safety, sustainability, and reproducibility. Three-dimensional architectures, advanced electrolytes, carbon- and MXene-based heterostructures, solid-state systems, self-healing materials, textile-integrated devices, and energy-harvesting-MSC hybrids provide promising directions. Ultimately, the technological relevance of an MSC should be determined by its ability to meet the requirements of a specific microsystem rather than by the highest capacitance reported under a particular laboratory condition. This systems-level perspective will be essential for moving MSCs from proof-of-concept demonstrations toward reliable wearable, flexible, autonomous, and microscale electronic platforms.

## 14. Conclusions

The review article collectively demonstrates the rapid evolution of microsupercapacitors (MSCs) from conventional planar energy-storage devices toward high-performance, flexible, stretchable, self-healing, and wearable power sources. Recent studies on graphene, MXene, MoS_2_/rGO, Ni-Mo-S/MXene, and other hybrid electrode architectures demonstrate that rational heterostructure design and improved electrode–electrolyte interfaces can substantially enhance charge-storage capability, rate performance, and cycling stability. In parallel, advances in laser patterning, inkjet printing, and three-dimensional fabrication provide promising routes toward high-resolution and scalable MSC manufacturing. Flexible and highly deformable architectures, including liquid-metal-based, kirigami-inspired, and 3D-printed MSCs, further demonstrate the possibility of maintaining electrochemical performance under severe mechanical deformation. Emerging systems integrating MSCs with triboelectric nanogenerators, wireless charging units, wearable sensors, and self-healing electrolytes highlight their potential as multifunctional power sources for autonomous electronics. In this context, this review provides a critical and comprehensive assessment of the fundamental charge-storage mechanisms, electrode and electrolyte materials, microfabrication strategies, device architectures, mechanical adaptability, performance-enhancement approaches, wearable integration, advanced MSC systems, reliability, safety, and sustainability. Beyond summarizing recent developments, the review identifies the persistent trade-offs between energy density, power density, mechanical robustness, scalability, and environmental compatibility and highlights future research directions toward practically deployable MSCs. Collectively, these developments establish MSCs as important enabling technologies for the continued miniaturization and autonomous operation of next-generation wearable and flexible electronic applications.

## Data Availability

No new data were created or analyzed in this study.
